# A hnRNPA2B1 agonist effectively inhibits HBV and SARS-CoV-2 omicron *in vivo*

**DOI:** 10.1093/procel/pwac027

**Published:** 2022-07-15

**Authors:** Daming Zuo, Yu Chen, Jian-piao Cai, Hao-Yang Yuan, Jun-Qi Wu, Yue Yin, Jing-Wen Xie, Jing-Min Lin, Jia Luo, Yang Feng, Long-Jiao Ge, Jia Zhou, Ronald J Quinn, San-Jun Zhao, Xing Tong, Dong-Yan Jin, Shuofeng Yuan, Shao-Xing Dai, Min Xu

**Affiliations:** Department of Medical Laboratory, School of Laboratory Medicine and Biotechnology, Southern Medical University, Guangzhou 510515, China; Microbiome Medicine Center, Department of Laboratory medicine, Zhujiang Hospital, Southern Medical University, Guangzhou 510515, China; Department of Medical Laboratory, School of Laboratory Medicine and Biotechnology, Southern Medical University, Guangzhou 510515, China; Department of Microbiology, Li Ka Shing Faculty of Medicine, The University of Hong Kong, Hong Kong 999077, China; Center for Pharmaceutical Sciences, Faculty of Life Science and Technology, Kunming University of Science and Technology, Kunming 650500, China; Center for Pharmaceutical Sciences, Faculty of Life Science and Technology, Kunming University of Science and Technology, Kunming 650500, China; Department of Medical Laboratory, School of Laboratory Medicine and Biotechnology, Southern Medical University, Guangzhou 510515, China; Department of Medical Laboratory, School of Laboratory Medicine and Biotechnology, Southern Medical University, Guangzhou 510515, China; Department of Medical Laboratory, School of Laboratory Medicine and Biotechnology, Southern Medical University, Guangzhou 510515, China; Center for Pharmaceutical Sciences, Faculty of Life Science and Technology, Kunming University of Science and Technology, Kunming 650500, China; Center for Pharmaceutical Sciences, Faculty of Life Science and Technology, Kunming University of Science and Technology, Kunming 650500, China; State Key Laboratory of Primate Biomedical Research; Institute of Primate Translational Medicine, Kunming University of Science and Technology, Kunming 650500, China; Department of Immunology, School of Basic Medical Sciences, Southern Medical University, Guangzhou 510515, China; Griffith Institute for Drug Discovery, Griffith University, Brisbane 4111, Australia; School of Life Sciences, Yunnan Normal University, Kunming 650500, China; State Key Laboratory of Primate Biomedical Research; Institute of Primate Translational Medicine, Kunming University of Science and Technology, Kunming 650500, China; School of Biomedical Sciences, Li Ka Shing Faculty of Medicine, The University of Hong Kong, Hong Kong 999077, China; Department of Microbiology, Li Ka Shing Faculty of Medicine, The University of Hong Kong, Hong Kong 999077, China; State Key Laboratory of Primate Biomedical Research; Institute of Primate Translational Medicine, Kunming University of Science and Technology, Kunming 650500, China; Center for Pharmaceutical Sciences, Faculty of Life Science and Technology, Kunming University of Science and Technology, Kunming 650500, China

**Keywords:** hnRNPA2B1, PAC5, HBV, SARS-CoV-2 omicron, TBK1-IRF3 pathway, type I IFNs

## Abstract

The twenty-first century has already recorded more than ten major epidemics or pandemics of viral disease, including the devastating COVID-19. Novel effective antivirals with broad-spectrum coverage are urgently needed. Herein, we reported a novel broad-spectrum antiviral compound PAC5. Oral administration of PAC5 eliminated HBV cccDNA and reduced the large antigen load in distinct mouse models of HBV infection. Strikingly, oral administration of PAC5 in a hamster model of SARS-CoV-2 omicron (BA.1) infection significantly decreases viral loads and attenuates lung inflammation. Mechanistically, PAC5 binds to a pocket near Asp49 in the RNA recognition motif of hnRNPA2B1. PAC5-bound hnRNPA2B1 is extensively activated and translocated to the cytoplasm where it initiates the TBK1-IRF3 pathway, leading to the production of type I IFNs with antiviral activity. Our results indicate that PAC5 is a novel small-molecule agonist of hnRNPA2B1, which may have a role in dealing with emerging infectious diseases now and in the future.

## Introduction

Emerging and reemerging viral diseases are a critical public health concern. Historically, hepatitis B virus (HBV) have cost millions of human lives. Currently, the COVID-19 pandemic is causing enormous economic losses and millions of deaths, closing of uncountable numbers of businesses (Sleigh 2005; Ledford 2022). The major challenge of HBV, a chronic viral disease, is to eliminate HBV covalently closed circular DNA (cccDNA) and to reduce the large antigen load, especially the hepatitis B surface antigen (HBsAg) (Fanning Gregory et al. 2019). While the major challenge of SARS-CoV-2, the causative agent of COVID-19 (Zhou et al. 2020), is the emerging novel genetic variants of SARS-CoV-2 with immune evasion capacity, particularly Delta (B.1.617.2) and Omicron (B.1.1.529) variants (Tao et al. 2021; Planas et al. 2022). Vaccine products are an effective means to prevent viral infection but cannot be used in clinical treatment, thus the discovery of novel classes of compounds with broad-spectrum antiviral activity is critical for developing therapeutic strategies to combat chronic and acute viral diseases. Currently, antiviral drugs approved for clinical use against HBV and SARS-CoV-2 belong to direct-acting antivirals (DAAs). For example, nucleoside/nucleotide analogues act as competitive inhibitors of viral DNA polymerase to treat chronic HBV infections, but its success is limited to eliminate HBV cccDNA and reduce the HBsAg (Fanning Gregory et al. 2019). Remdesivir and molnupiravir target the viral RNA-dependent RNA polymerase (Beigel et al. 2020; Owen et al. 2021; Wahl et al. 2021), while paxlovid targets the coronavirus (CoV)’s main protease (M^pro^) (Owen et al. 2021; Wahl et al. 2021) to treat COVID-19. However, drug resistance and side effects are often a critical problem. On the other hand, host-directed antivirals (HDAs) are currently under investigation, which is exampled by the transmembrane serine protease 2 inhibitor (N-0385) for COVID-19, and the liver-targeted toll-like receptor 8 agonist (GS-9688) for HBV disease, respectively (Amin et al. 2021; Daffis et al. 2021; Zhang et al. 2022).

In this study, we report a novel compound PAC5 that effectively prevented HBV and SARS-CoV-2 omicron infection *in vivo*. We demonstrated that PAC5 functions as an agonist of heterogeneous nuclear ribonucleoprotein A2B1 (hnRNPA2B1). Of note, hnRNPA2B1 functions as a downstream effector of the *N*^6^-methyladenosine (m^6^A) mark and participates in RNA biology and the regulation of transcriptional responses (Villarroya-Beltri et al. 2013; Alarcon et al. 2015; Wu et al. 2018; Liu and Shi 2021). hnRNPA2B1 plays a vital role in the antiviral innate immune response (Liu and Shi 2021). As an RNA-binding protein, hnRNPA2B1 affects the polyribosomal distribution of HIV-1 RNA by modulating nuclear and cytoplasmic trafficking events (Gordon et al. 2013). hnRNPA2B1 functions as a nuclear sensor of both self- and pathogen-derived DNA, too (Wang et al. 2019). However, draggability of hnRNPA2B1 has been rarely explored.

We found that PAC5 inhibits HBV in HepG2.2.15, HepG2 (transfected with PBSK-rtM204I), HepAD38 cells and in the primary human hepatocytes with HBV infection. PAC5 was also efficacious in two distinct mice models of HBV infection, as indicated by significantly eliminating HBV cccDNA level and reducing HBV-related antigen expression. Strikingly, oral administration of PAC5 dramatically ameliorated the lung damage induced by SARS-CoV-2 omicron (BA.1) infection in a hamster model and significantly suppressed the viral infection. Our study indicates that PAC5 is a highly selective and potent agonist of hnRNPA2B1 that is new potential drug target for antiviral immunotherapy. PAC5 will be developed as a host-directed antivirals (HDAs).

## Results

### PAC5 inhibited HBV infection *in vitro* and *in vivo* with negligible toxicity

**Figure 1.**
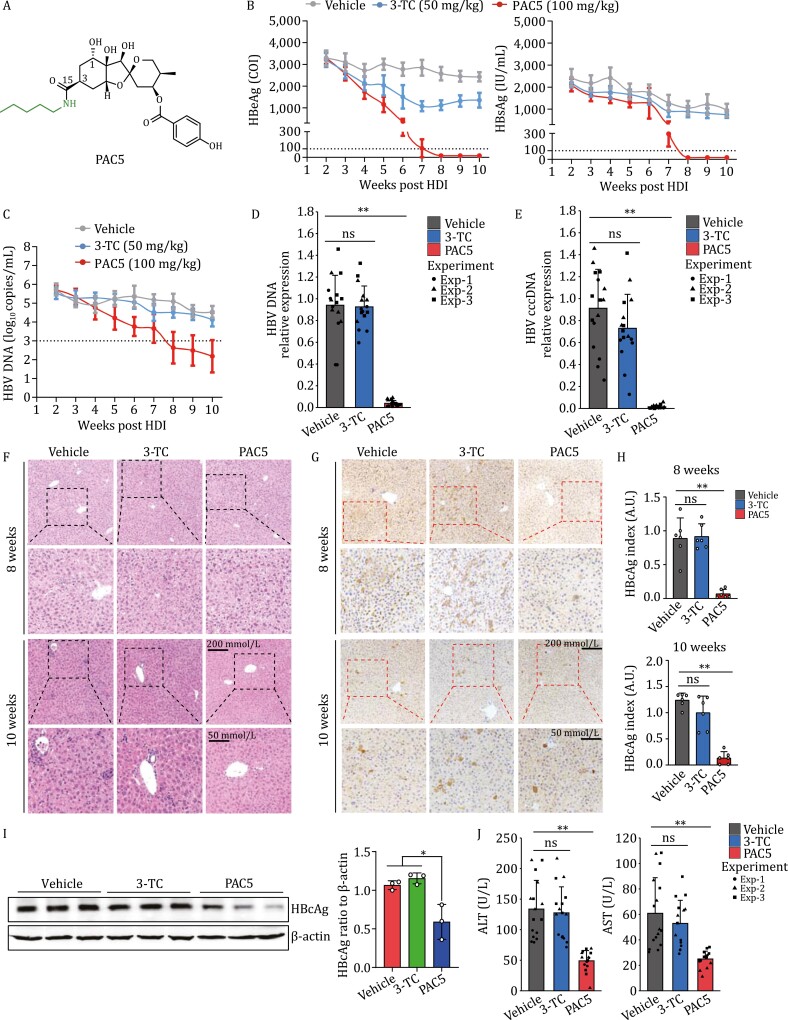
PAC5 inhibited HBV infection *in vitro* and *in vivo*. (A) The chemical structure of PAC5. (B–J) C57BL/6 mice were hydrodynamically injected with 10 μg of PBSK-rtM2041 plasmid through the tail vein. Two weeks later, the mice were assigned to three groups (*n* = 6 per group) and gavaged daily with PBS, 3-TC (50 mg/kg), or PAC5 (100 mg/kg). The treatment was stopped at week 8, and the mice were sacrificed at week 10. (B) The levels of HBsAg and HBeAg in sera were measured by chemiluminescence microparticle immunoassay (CMIA) at indicated time points. (C) HBV DNA was measured by real-time PCR analysis. (D and E) The mRNA expression of HBV DNA (D) and HBV cccDNA (E) in the liver tissues were detected by quantitative PCR analysis. (F) The histologic analysis of livers was performed using H&E staining. (G) The expression of HBcAg in liver tissues was determined by immunochemistry staining. (H) Statistical graph of positive expression rate of HBcAg in the liver tissues. (I) The protein levels of HBcAg in the liver tissues were evaluated by immunoblotting. (J) The sera ALT and AST activities were detected at week 10. Three independent experiments were performed. All data were presented as means ± SD. *P < 0.05, **P < 0.01, ns, not significant, calculated by one-way ANOVA with Bonferroni post hoc test (D, E, H–J).

We profiled our library of natural products encompassing approximately 1000 small molecules to identify some anti-HBV natural sesquiterpenoid phyllanthacidoid A (PA) derivatives. Then we structurally modified PA and synthesized 38 compounds in an iterative structure–activity relationship guided manner. Among them, PAC5 showed the most potent anti-HBV activity in cell-based assays. PAC5 (Fig. 1A) is a small synthetic molecule obtained from PA (Fig. S1A) by replacing the C-15 ester side chain with an *N*-pentyl amide group (Fig. S1B). The *N*-pentyl amide group was found critical to the anti-HBV activity in the structure–activity relationship study of PAC5 (Fig. S1A–C and Table S1). PAC5 exhibited inhibitory activity against HBV in HepG2.2.15, HepG2 (transfected PBSK-rtM204I), and HepAD38 cells (Fig. S2A and S2B). Most importantly, PAC5 significantly reduced the HBV DNA levels in the primary human hepatocytes with HBV infection (Fig. S2C). Notably, PAC5 did not show cytotoxicity in the HBV-infected HepG2 hepatocyte cell lines (SI >1,000) (Table S1).

Next, we examined the inhibitory effect of PAC5 on HBV infection *in vivo*. Mice were pre-injected with rAAV8-1.3HBV into the tail vein to generate a mouse model of *in vivo* HBV infection. Oral administration of PAC5 significantly reduced HBsAg, hepatitis B e antigen (HBeAg), and HBV DNA levels in serum, as well as eliminated HBcAg, HBV DNA, and cccDNA levels in liver tissues of mice infected with HBV (Fig. S2D–G). Besides, the liver function of viral-infected mice was significantly improved by PAC5 (Fig. S2H). Furthermore, mice were injected with the PBSK-rtM204I plasmid via the tail vein to establish a mouse model of *in vivo* infection with drug-resistant HBV. The infected mice were treated with PAC5 (at a dose of 100 mg/kg), 3-TC (at a dose of 50 mg/kg), and physiological saline once per day for 8 weeks. As shown in Fig. 1B, at 8th week, PAC5 totally eliminated HBsAg and HBeAg secretion in serum. PAC5 reduced HBV titers by 3.3 log_10_ copies/mL versus those of 3-TC and vehicle-treated mice (Fig. 1C). PAC5 significantly limited the levels of HBV DNA and cccDNA in the liver tissues of virus-infected mice (Fig. 1D and 1E). PAC5-treated mice exhibited few necrosis of hepatocytes with limited infiltration of inflammatory cells versus the vehicle- and 3-TC-treated group (Fig. 1F). The infiltration of lymphocytes was also alleviated in the PAC5-treated mice indicated by CD3 staining (Fig. S2I). PAC5 extensively inhibited the hepatic expression of HBcAg (Fig. 1G–I). In addition, key liver function indexes were significantly improved in the PAC5-treated mice versus the 3-TC and vehicle-treated controls (Fig. 1J).

Moreover, PAC5 was evaluated for its toxicity in KM mice. In an acute toxicity experiment, no mice died after oral administration of PAC5 (2,000 mg/kg) (Fig. S3). In a repeated-dose toxicity study, administration of PAC5 by 2,000 mg/kg twice daily for 14 consecutive days did not result in toxicity (Table S2).

### PAC5 targets hnRNPA2B1 and resides in RRM1 domain of hnRNPA2B1 via contacting Asp49

**Figure 2.**
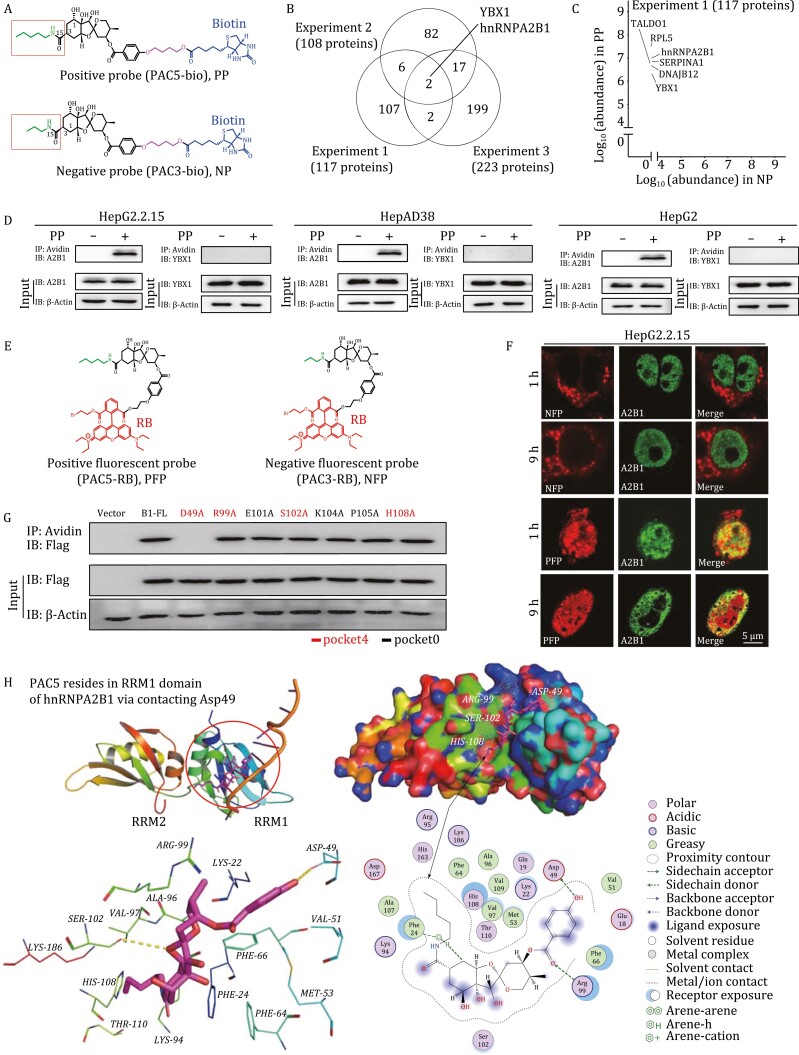
PAC5 targets hnRNPA2B1 and resides in RRM1 domain of hnRNPA2B1. (A) The chemical structures of positive probe (PAC5-bio, PP) and negative probe (PAC3-bio, NP). (B) Immobilized biotinylated probes were incubated with HepG2.2.15 cell lysates. Then the PAC5-associated protein from three independent experiments was analysed by mass spectrometry. The number of specific proteins in the positive probe pull-downs was shown by a Venn diagram. (C) The scatterplot showing the 117 specific proteins identified in sample of positive probe (PAC5-bio). (D) The interaction of PAC5 with the candidate proteins was validated by co-precipitation assay in three cell lines. (E) The chemical structure of rhodamine B-labeled PAC5 and PAC3 (PFP and NFP, respectively). (F) The colocalization of PFP (red) and hnRNPA2B1 (green) was determined by confocal assay in HepG2.2.15 cells for indicated time. (G) HEK293T cells were transfected with hnRNPA2B1, hnRNPA2B1-D49A, hnRNPA2B1-R99A, hnRNPA2B1-E101A, hnRNPA2B1-S102A, hnRNPA2B1-K104A, hnRNPA2B1-P105A and hnRNPA2B1-H108A, respectively. The cell lysates were incubated with biotinylated PAC5, then immunoprecipitated with streptavidin and examined by anti-Flag antibodies by immunoblot analysis. (H) The predicted binding mode of PAC5 to hnRNPA2B1. (Top panel) The overview of PAC5 binding to the pocket 4 in RRM1 domain of hnRNPA2B1 using cartoon and surface representation. (Bottom panel) Close-up view of the binding of PAC5 to the pocket 4 of hnRNPA2B1. Pocket 4 was highlighted with a red circle. The groove embedded by *N*-pentylamine of PAC5 is marked with an arrow. The compound PAC5 and side chains of the residues involved in binding are shown as sticks. Secondary structural elements were colored from blue (N-terminus) to red (C-terminus). Three independent experiments were performed. One representative experiment was showed.

As shown in Fig. S4A, the strategy of activity-based protein profilings (ABPPs) was used to explore the functional target(s) of PAC5. We synthesized both a positive probe (PAC5-bio, PP) and a negative probe (PAC3-bio, NP) from PAC5 and PAC3 (an inactive analogue of PAC5), respectively, with biotin and an ethylene glycol linker (Figs. 2A and S1D). Though PAC5 and PAC3 share the same skeleton, they differ in the length of the C-15 side chain. PAC5 has a 5-carbon amide side chain at C-15, while PAC3 with a 3-carbon amide group (Fig. 2A). Biotin-tagged positive probe (PAC5-bio, PP) retained anti-HBV activity (Fig. S4B and Table S1), while the biotin-tagged negative probe (PAC3-bio, NP) did not show antiviral activity (Fig. S4C).

Then, we performed three independent pull-down experiments using positive and negative probes, respectively. The pull-downs of negative probe were used to remove false positive proteins that exist the positive probe pull-downs. Three independent mass spectrometry analysis identified a total of 117, 108, and 223 proteins that only exist in the positive probe pull-downs and are absent the negative probe pull-downs, respectively (Fig. 2B). Among them, hnRNPA2B1 and YBX1 are common proteins in the three independent experiments (Fig. 2B). Moreover, hnRNPA2B1, YBX1, RPL5, SERPINA1, DNAJB12, and TALDO1 were the top proteins with the greatest abundance (Figs. 2C and S4D). We further used immunoprecipitation (IP) assays to monitor the candidate proteins in HepG2.2.15, HepAD38, and HepG2 cells. We found that only hnRNPA2B1 was specifically pulled down by the positive probe (Figs. 2D and S4E).

To further confirm whether PAC5 directly binds hnRNPA2B1, we synthesized positive and negative fluorescent probes with a rhodamine B (RB) tag attached (Figs. 2E and S1E), respectively. The positive fluorescent probe (PAC5-RB, PFP) retained anti-HBV activity, while the negative fluorescent probe (PAC3-RB, NFP) did not show efficient antiviral activity (Fig. S5A). The positive fluorescent probe (PAC5-RB, PFP) exhibited significant colocalization with hnRNPA2B1 in HepG2.2.15, HepAD38, and HepG2 cells (Figs. 2F, S5B and S5C), respectively.

The binding mode of PAC5 was studied through pocket identification, molecular docking, and point mutation experiments. Two potential binding pockets for PAC5 (pocket 0 and pocket 4) were found in the hnRNPA2B1 protein (see Methods and Fig. S6A and S6B). Pocket 0 and pocket 4 locate in the RRM2 and RRM1 regions, respectively. Based on the docking results, Glu101, Lys104, and Pro105 in pocket 0, or Asp49, Arg99, Ser102, and His108 in pocket 4 are potential key residues for PAC5 (Fig. S6C and S6D). Point mutation experiments demonstrated that the mutation of Asp49 in pocket 4, but not other mutations, significantly diminished the binding of PAC5 to hnRNPA2B1 (Fig. 2G). Taken together, these findings suggested that Asp49-contained pocket 4 in RRM1 domain is most likely the PAC5-binding site in hnRNPA2B1 (Fig. 2H).

### The binding of PAC5 to hnRNPA2B1 activates TBK1-IRF3 signaling

**Figure 3.**
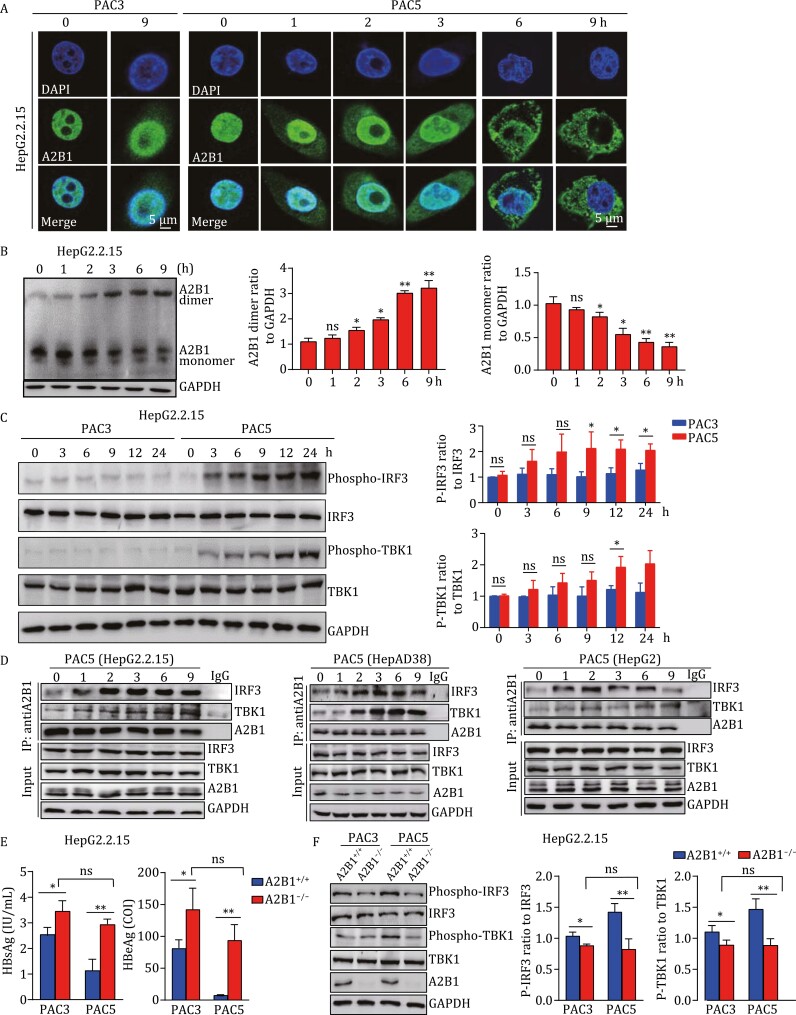
TBK1-IRF3 signaling activation attributes to PAC5-bound hnRNPA2B1 being dimerized. (A) HepG2.2.15 cells were treated with PAC3 (2 μmol/L) or PAC5 (2 μmol/L) for indicated time points. The intracellular localization of hnRNPA2B1 (green) was determined by confocal microscopy. The nuclei are stained with DAPI (blue). (B) HepG2.2.15 cells were treated with PAC5 (10 μmol/L) for the indicated time and cell lysates were prepared for native PAGE and hnRNPA2B1-dimerization assay. (C) HepG2.2.15 cells were treated with PAC5 (2 μmol/L) or PAC3 (2 μmol/L) for the indicated time. Phosphorylations of TBK1 and IRF3 were detected by immunoblotting assay. (D) HepG2.2.15, HepAD38, and HepG2 cells were cultured with PAC5 (2 μmol/L) as indicated time, and the cell lysates were immunoprecipitated with anti-hnRNPA2B1 or IgG. The components (IRF3, TBK1, hnRNPA2B1) in the complex were examined by western blotting. (E) hnRNPA2B1^+/+^ and hnRNPA2B1^−/−^ HepG2.2.15 cells were treatment with PAC5 (2 μmol/L) or PAC3 (2 μmol/L) for 24 h. The productions of HBsAg and HBeAg were detected in the cell culture supernatants via CMIA. (F) hnRNPA2B1^+/+^ and hnRNPA2B1^−/−^ HepG2.2.15 cells were treated with PAC5 (2 μmol/L) or PAC3 (2 μmol/L) for 9 h, the phosphorylations of TBK1 and IRF3 were determined by western blotting analysis. Three independent experiments were performed. All data were presented as means ± SD. *P < 0.05, **P < 0.01, ns, not significant, calculated by one-way ANOVA with Bonferroni post hoc test (B, C) and two-way ANOVA with Bonferroni post hoc test (E, F), respectively.

TANK-binding kinase 1 (TBK1) is a critical serine/threonine protein kinase that mediates the phosphorylation and nuclear translocation of IFN regulatory factor 3 (IRF3). The TBK1-IRF3 pathway can be activated by hnRNPA2B1 dimers in the cytoplasm (Wang et al. 2019). Herein, immunofluorescence analysis indicated that PAC5 significantly promoted the translocation of hnRNPA2B1 from the nucleus into the cytoplasm, while PAC3 did not affect the translocation of hnRNPA2B1 in HepG2.2.15, HepAD38, and HepG2 cells (Figs. 3A and S7A). Accordingly, the native gel electrophoresis assay confirmed that PAC5 promoted hnRNPA2B1 dimerization (Fig. 3B). Furthermore, we found that PAC5 markedly enhanced the phosphorylation of TBK1 and IRF3 (Figs. 3C and S7B) in HepG2.2.15 and HepAD38. PAC5 also enhanced the phosphorylation of TBK1 and IRF3 in the non-HBV-infected HepG2 cell (Fig. S7C). Noteworthy, PAC5 promoted the phosphorylation of TBK1 and IRF3 in primary human hepatocytes with or without HBV infection (Fig. S7D). These results suggested that the PAC5-hnRNPA2B1 association facilitates TBK1 activation and the effect is not viral infection-dependent. Interestingly, PAC5 enhanced the association of hnRNPA2B1 with TBK1 and IRF3 both in HBV-transfected cells and non-HBV-infected cells (Fig. 3D). Thus, PAC5 functions as an activator of hnRNPA2B1 that causes the initiation of the TBK1-IRF3 pathway.

To further confirm that PAC5-hnRNPA2B1 association activates TBK1 and suppresses HBV replication, we employed the CRISPR/Cas9 system to establish hnRNPA2B1-deficient HepG2, HepG2.2.15, and HepAD38 cells. The results showed that hnRNPA2B1 deficiency diminished the ability of PAC5 to limit HBsAg and HBeAg secretion from HepG2.2.15 and HepAD38 cells (Figs. 3E and S8A). Moreover, hnRNPA2B1 deficiency significantly impaired the activation of IRF3 and TBK1 upon PAC5 stimulation (Figs. 3F, S8B and S8C). Notably, the phosphorylation of IRF3 and TBK1 was similar in hnRNPA2B1-deficient cells cultured with or without PAC5. Together, these data indicate that PAC5 binds hnRNPA2B1 to activate the TBK1-IRF3 pathway, which accounts for PAC5-induced anti-HBV innate responses.

### hnRNPA2B1 is responsible for PAC5-initiating type I IFN-mediated activation of STAT1/2

**Figure 4.**
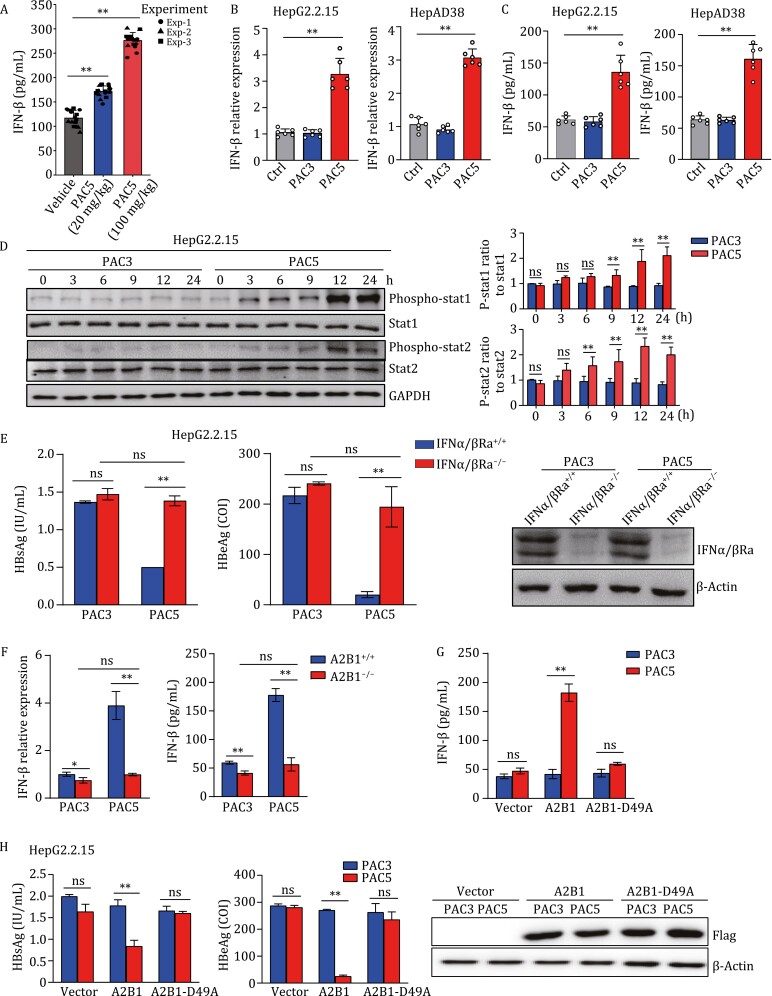
hnRNPA2B1 is responsible for PAC5-initiating type I IFN-mediated activation of STAT1/2. (A) After 2 weeks of intravenous injection of rAAV8-1.3HBV ayw (1 × 10^11^ vg/mouse), C57BL/6 mice were assigned to 3 groups (*n* = 6 per group) and intragastric administration daily with PBS, PAC5 (20 mg/kg), or PAC5 (100 mg/kg). The mice were stopped intragastric administration at week 8 and sacrificed at week 10. The serum IFN-β levels were evaluated by ELISA analysis at the end of the experiment. (B) HepG2.2.15 and HepAD38 cells were treated with or without PAC3 (2 μmol/L) and PAC5 (2 μmol/L) for 9 h. mRNA levels of the IFN-β in the HBV-infected cells were detected by quantitative RT-PCR analysis. (C) HepG2.2.15 and HepAD38 cells were treated with or without PAC3 (2 μmol/L) and PAC5 (2 μmol/L) for 24 h. IFN-β concentrations in the culture supernatant were assayed by ELISA. (D) HepG2.2.15 cells were treated with PAC5 (2 μmol/L) or PAC3 (2 μmol/L) for the indicated time. Phosphorylated and total stat1 or stat2 proteins were detected by immunoblotting. (E) IFN-α/βRα^+/+^ and IFN-α/βRα^−/−^ HepG2.2.15 cells were treated with PAC3 (2 μmol/L) and PAC5 (2 μmol/L) for 24 h, the levels of HBsAg and HBeAg in the cell culture supernatants were analysed via CMIA. (F) hnRNPA2B1^+/+^ and hnRNPA2B1^−/−^ HepG2.2.15 cells were treated with PAC3 (2 μmol/L) or PAC5 (2 μmol/L) for 9 h, the mRNA level of IFN-β was detected by quantitative RT-PCR analysis. The culture supernatants were collected at 24 h after PAC5 stimulation and IFN-β concentrations were assayed by ELISA. (G and H) hnRNPA2B1^−/−^ HepG2.2.15 cells were transfected with vector, hnRNPA2B1 or hnRNPA2B1-D49A, then treated with PAC3 (2 μmol/L) or PAC5 (2 μmol/L) for another 24 h. The amount of IFN-β protein in the cell culture supernatants was evaluated via ELISA (G) and the productions of HBsAg and HBeAg were detected by CMIA (H). Three independent experiments were performed. One representative experiment was showed. All data were presented as means ± SD. *P < 0.05, **P < 0.01, ns, not significant, calculated by one-way ANOVA with Bonferroni post hoc test (A–C) and two-way ANOVA with Bonferroni post hoc test (D–H), respectively.

We demonstrated that PAC5 activates TBK1-IRF3 signaling. Activation of TBK1-IRF3 was reported to initiate IFN-I production (McNab et al. 2015). Therefore, we examined the effect of PAC5 on type I IFN response by transcriptomics. The results showed a significant difference between samples treated with PAC3 and PAC5 (Fig. S9A and S9B). In the PAC5-treated samples, a total of 962 and 568 genes were upregulated and downregulated, respectively (Fig. S9C). Gene set enrichment analysis (GSEA) found that PAC5 activated the RNA splicing, RNA processing, and RNA translation but inhibited cell adhesion and proliferation (Fig. S9D). Notably, PAC5 also activated the type I IFN signaling pathway (Fig. S9E). Furthermore, ELISA revealed that PAC5 treatment significantly increased the production of IFN-β in HBV-infected mice (Fig. 4A). *In vitro* analysis showed that PAC5 enhanced the mRNA levels of IFN-β in HepG2.2.15 and HepAD38 cells (Fig. 4B). Subsequently, we measured the protein level of IFN-β secreted into the supernatant from PAC5-stimulated HepG2.2.15 and HepAD38 cells. The results showed that PAC5 significantly upregulated IFN-β secretion (Fig. 4C). PAC5 also promoted the expression of IFN-β in HBV-infected primary human hepatocytes (Fig. S10A). In addition, PAC5 treatment promoted the production of IFN-α in HBV-infected cells (Fig. S10B). Moreover, PAC5 sharply increased the mRNA production of IFN-stimulated genes (ISGs) in both HepG2.2.15 and HepAD38 cells (Fig. S10C). Type I IFN-activated JAK-STAT signaling is essential for inhibiting viral infection in HBV-infected cells (Gonzalez-Navajas et al. 2012). Indeed, PAC5 treatment significantly promoted the phosphorylation of STAT1 and STAT2 in HBV-infected cells (Figs. 4D and S10D).

To further determine whether PAC5-mediated inhibition of HBV replication is type I IFN signaling-dependent, we employed the CRISPR/Cas9 system to knock out the IFN-α/β receptor (IFNα/βR) in HepG2.2.15 and HepAD38 cells. IFNα/βR deficiency diminished the suppressive effect of PCA5 on HBsAg and HBeAg secretion (Figs. 4E and S10E). Moreover, IFN-β was significantly downregulated in PAC5-stimulated hnRNPA2B1^−/−^ cells at both the transcriptional and translational levels (Figs. 4F and S10F). The impaired IFN-β production in hnRNPA2B1^−/−^ cells could be rescued by overexpressing hnRNPA2B1 but not hnRNPA2B1-D49A (Figs. 4G and S10G). Consistently, PAC5 did not influence HBV-associated antigen secretion in hnRNPA2B1^−/−^ cells overexpressing hnRNPA2B1-D49A (Figs. 4H and S10H). In summary, we revealed that the binding of PAC5 to hnRNPA2B1 activates TBK1-IRF3 signaling, initiates type I IFN production, and subsequently promotes the phosphorylation of STAT1/2 in HBV-infected cells.

### PAC5 inhibits SARS-CoV-2 omicron lung infection presumably through hnRNPA2B1 based on experimental evidence of MHV-A59 infection in L929 cells

**Figure 5.**
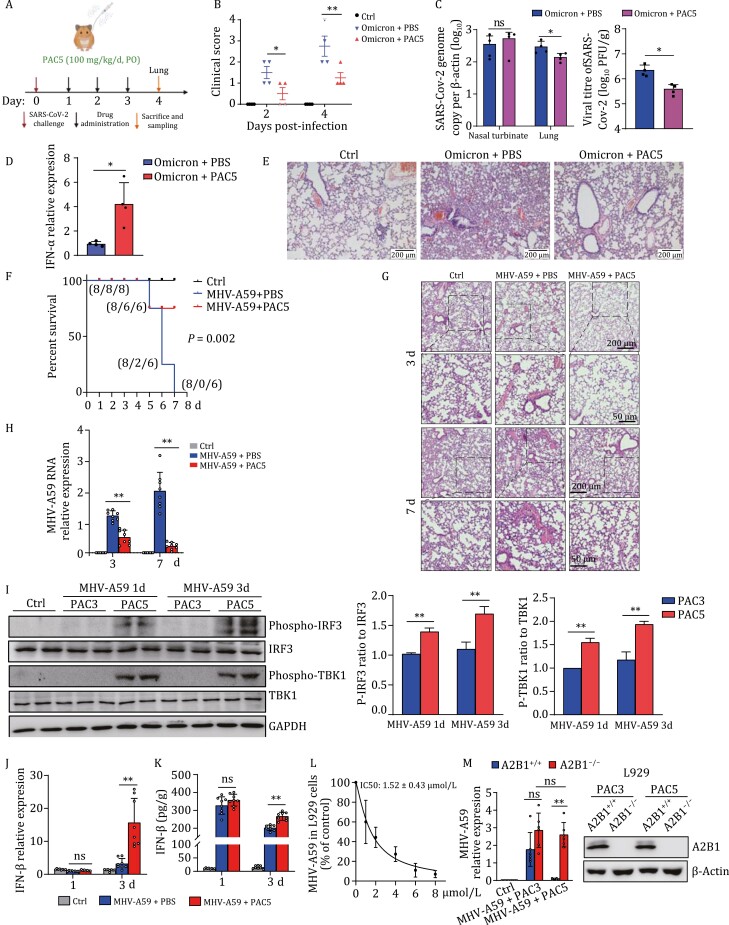
PAC5 inhibits coronavirus infection in animal lungs. (A–E) 6–10 weeks old Syrian hamsters were assigned to three groups (*n* = 4 per group). Omicron-infected hamsters were intranasally inoculated with the SARS-CoV-2 omicron virus of 10^5^ PFU. The uninfected animals (Ctrl) and Omicron+PBS group hamsters were intragastric administration daily with PBS. The Omicron+PAC5 group hamsters were daily orally administered with PAC5 (100 mg/kg). (A) Experimental scheme of SARS-CoV-2 challenge and drug treatment in hamsters. (B) Clinical scores. A score of 1 was given to each of the following clinical signs: lethargy, ruffled fur, hunchback posture, and rapid breathing. (C) Viral load (measured by qRT-PCR analysis) and infectious titer (measured by plaque assay) after PAC5 treatment. (D) mRNA expression of hamster IFN-α in the lung tissue. (E) Representative images of lung pathogenicity. (F–M) Six-week-old C57BL/6 mice were assigned to 3 groups (*n* = 8 per group). MHV-A59-infected mice were intranasally inoculated with the MHV-A59 virus of 1.5 × 10^6^ PFU. The uninfected mice (Ctrl) and MHV-A59+PBS group mice were intragastric administration daily with PBS. The MHV-A59+PAC5 group mice were intragastric administration with PAC5 (100 mg/kg). (F) Survival evaluation. (G) Representative H&E staining images of the lungs from mice at indicated time in the different groups. (H) The levels of MHV-A59 in the mouse lung at the indicated time were measured by quantitative PCR analysis. (I) Phosphorylated and total IRF3 or TBK1 protein in the mouse lung tissue were detected by immunoblotting assay. (J) mRNA levels of IFN-β in the mouse lung at the indicated time were measured by quantitative RT-PCR analysis. (K) The amount of IFN-β protein in the lung tissue at 3 d was evaluated via ELISA. (L) L929 cells were infected with MHV-A59 (MOI = 0.5). After 2 h, cells were treated with PAC5 at the indicated concentration for 24 h. Viral loads of MHV-A59 in the cells were measured by quantitative PCR analysis. (M) hnRNPA2B1^+/+^ and hnRNPA2B1^−/−^ L929 cells were infected with MHV-A59 (MOI, 0.5). After 2 h, cells were treated with PAC5 (4 μmol/L) or PAC3 (4 μmol/L) for another 24 h. The levels of MHV-A59 in the cells were measured by quantitative PCR analysis. All data were presented as means ± SD. *P < 0.05, **P < 0.01, ns, not significant, calculated by unpaired Student’s *t*-test (C, D, L), the log-rank (Mantel-Cox) test (F), one-way ANOVA with Bonferroni post hoc test (B, H, J, K) and two-way ANOVA with Bonferroni post hoc test (M), respectively.

Despite population-wide vaccination campaigns in many countries, severe acute respiratory syndrome coronavirus 2 (SARS-CoV-2) continues to disseminate globally more than 2 years since the pandemic started due to the high prevalence of re-infection and vaccine-breakthrough infections among individuals with waning neutralizing antibody titers (Abedi et al. 2021; Gardner and Kilpatrick 2021). The recently emerged omicron variant of SARS-CoV-2 has rapidly replaced the Delta variant as the predominant circulating variant with higher transmissibility and immune evasiveness, which considerably compromise the authorized monoclonal antibodies in the market. In this case, novel antiviral agents with broad-spectrum coverage are urgently needed for the control of current and future CoV diseases. Herein, we used the established golden Syrian hamster models to determine the *in vivo* antiviral potential of PAC5 against SARS-CoV-2 omicron infection (Yuan et al. 2021). Hamsters were once daily oral administrated with PAC5 (at a dose of 100 mg/kg) or vehicle control (PBS) after being challenged with 10^5^ PFU/hamster of SARS-CoV-2 omicron virus (Fig. 5A). An improvement in clinical score was achieved in the PAC5 treated hamsters after virus infection, compared with the groups treated with PBS only (Fig. 5B). Notably, PAC5 treatment led to a significant reduction of virus titer in the lung tissue from infected hamsters (Fig. 5C). While PAC5 has no significant effect on the virus titer in Nasal turbinate. The result is similar to those previously reported (Zhou et al. 2021; Chiba et al. 2022). Treatment with systemic neutralizing antibodies or small molecule drugs (Favipiravir and Remdesivir) can significantly reduce infection in the lung but not in the nasal turbinate. Considering the intragastric administration with PAC5, the possible reason is that there exist differences in the absorption, distribution and metabolism of PAC5 in different tissues, especially between nasal turbinate and lung. In line with the previous observation in HBV infection, PAC5 promoted the type I IFN (IFN-α) production in the virus-infected hamsters (Fig. 5D). The data for IFN-β in hamster lung is not shown as qPCR Ct value > 35. Importantly, histological examination of lung tissue obtained from SARS-CoV-2 omicron-infected hamsters revealed attenuated lung pathological changes in PAC5-treated mice compared to the control animals (Fig. 5E). Therefore, PAC5 holds the potential to reduce the COVID-19 diseases *in vivo*.

The mouse hepatitis virus (MHV)-A59 is a murine CoV endemic in wild mice, which could induce an acute, self-resolving respiratory infection that recapitulates acute pneumonia experienced by most SARS-CoV-2-infected individuals (Guo et al. 2021). To evaluate the cross protection of PAC5 for both human and murine CoV, we assessed the potential mechanism of PAC5 on CoV lung infection in mice using the MHV-A59 virus. We first intranasally (i.n.) infected C57BL/6 mice with MHV-A59 virus. Then, PAC5 was orally administrated. Excitingly, PAC5 significantly improved the survival of virus-infected mice (Fig. 5F). Histopathological analysis showed that pulmonary interstitial damage was alleviated in the PAC5-treated group (Fig. 5G). Further, the viral titer of MHV-A59 in the PAC5-treated group was significantly reduced (Fig. 5H). Increased phosphorylated TBK1 and IRF3 were observed in the lung tissues of virus-infected mice upon PAC5 administration compared to negative controls (Fig. 5I). Additionally, PAC5 treatment significantly enhanced the production of IFN-β in the mouse lung tissues at the early phase of MHV-A59 infection, as determined at both mRNA and protein levels (Fig. 5J and 5K). The data for IFN-α in mouse lung is not shown as qPCR Ct value > 35. These results suggested that PAC5 protected the mice from MHV-A59 lung infection.

To evaluate the target and mechanism of PAC5, we tested the antiviral activity of PAC5 in L929 cells (mouse lung fibroblasts) infected with MHV-A59. PAC5 treatment significantly reduced the amount of virus (Fig. 5I). Most importantly, hnRNPA2B1 deficiency abrogated the anti-CoV ability of PAC5 in L929 cells (Fig. 5M). These data suggested that the PAC5-mediated anti-CoV effect is also hnRNPA2B1-dependent.

## Discussion

hnRNPA2B1, an RNA-binding protein and member of the hnRNP family, plays an essential role in RNA biology and various diseases (Liu and Shi 2021). Recently, hnRNPA2B1 has been suggested as an attractive regulatory protein for viral infection (Wang et al. 2019). The activation of hnRNPA2B1 enhances the phosphorylation of TBK1 (Wang et al. 2019). TBK1, an activator of innate immunity, functions as a signaling intermediate upstream of IFN-I and pro-inflammatory cytokines production (Taft et al. 2021). Activation of the TBK1-IRF3 signaling pathway promotes host antiviral innate immune responses (Wang et al. 2019). However, no small molecule targeting hnRNPA2B1 has been reported thus far. Herein, we show that PAC5 is the first small-molecule agonist of hnRNPA2B1 for the inhibition of viral infection. In mice infected with wild-type and drug-resistant HBV, oral administration of PAC5 significantly reduced the synthesis of HBV DNA, cccDNA, and viral proteins. Besides, PAC5 exhibited a capacity for improving liver function in virus-infected mice. Notably, PAC5 showed no toxicity in cell-based assays and an excellent safety profile in mice. Furthermore, our ABPPs study indicated that the principal target of PAC5 is hnRNPA2B1, a previously unknown drug target for viral infection. Moreover, our data indicated that PAC5 promotes formation of the hnRNPA2B1 dimer and its translocation to the cytoplasm, where it activates the TBK1-IRF3 pathway, leading to IFN-β production and STAT1/2 phosphorylation.

hnRNPs represent a large family of RNA-binding proteins involved in the processing, transport, and translation of mRNA (Alarcon et al. 2015). The *HNRNPA2B1* gene encodes two major protein isoforms, hnRNP A2 and hnRNP B1, through alternative splicing. hnRNP B1 contains an additional 12 amino acids at its N-terminus (Wu et al. 2018). hnRNPA2B1 contains two tandem RNA/DNA-recognition motifs (RRM1 and RRM2) at its N-terminus and a Gly-rich low-complexity region at its C-terminus. In this study, we firstly identified five pockets in the structure of hnRNPA2B1 using Fpocket software. The molecular docking results suggested that pockets 0 and 4 are relatively favorable for PAC5. Further point mutation experiments found that residue Asp49 of pocket 4 is essential for the binding of PAC5 with hnRNPA2B1. Therefore, we inferred that the anti-HBV activity of PAC5 is achieved by its binding to pocket 4. We observed that the *N*-pentyl side chain of PAC5 is deeply embedded in the groove of pocket 4. This may explain why the length of the *N*-alkyl side chain of the sesquiterpenoid series has a critical influence on anti-HBV activity. Pocket 4 locates in the RRM1 domain and overlaps with the RNA-binding pocket. The RRM domains are required for hnRNPA2B1 dimer formation (Wang et al. 2019). Taken together, through structural bioinformatics and experimental validation, our results suggested that targeting RRM1 by PAC5 might be an effective strategy to modulate the hnRNPA2B1 function. PAC5-bound hnRNPA2B1 is extensively activated and initiates the TBK1-IRF3 pathway, leading to the production of type I IFNs.

IFN systems are essential for antiviral defense and are functionally non-redundant (Mueller et al. 1994; Sadler and Williams 2008). During infection with viruses, Type I IFNs have diverse effects on innate and adaptive immune cells (McNab et al. 2015). Indeed, induction of IFN and the innate antiviral response in chronic hepatitis B patients could be expected to reduce HBV DNA levels and the production of HBsAg and HBeAg. A recent study identified hnRNPA2B1 as a nuclear receptor for viral DNA critical for host antiviral responses (Wang et al. 2019). Our data revealed that PAC5, a highly selective and potent agonist of hnRNPA2B1, initiates the TBK1-IRF3 pathway leading to upregulation of type I IFNs, phosphorylation of STAT1/2, and inhibition of viral infection *in vivo* and *in vitro*.

Notably, PAC5 also suppressed lung infection of SARS-CoV-2 omicron in mice. Growing evidence indicated that COVID-19 patients have a robust type I IFN response in the early infection phase (Lee and Shin 2020). However, SARS-CoV-2 evades type I IFN by disrupting the TBK1-IKKε-IRF3 signalosome and degrading IRF3 and IKKε (Wu et al. 2020; Xia et al. 2020b; Schmidt et al. 2021). Herein, we show that PAC5 treatment markedly enhanced TBK1 activation, which in turn led to the induction of IFN-I in different mice models of CoVs lung infection. Meanwhile, our study confirmed that hnRNPA2B1 is responsible for PAC5-induced TBK1 activation in CoVs-infected cells. In summary, our study suggested hnRNPA2B1 a druggable target with broad-relevance to virus infection. It is known that HBV is a DNA virus and SARS-CoV-2 is an RNA virus, which activate cGAS-STING and RLR pathways, respectively. As TBK-IRF3 signaling is the downstream of cGAS-STING and RLR, it is unclear whether the inhibition of these pathways can affect the activation of TBK-IRF3 by PAC5. It is worth conducting these experiments in our future work.

Bioactive natural products offer opportunities to discover novel targets and mechanisms for treating human diseases (Ying et al. 2007; Newman and Cragg 2020). Herein, we discovered a novel compound PAC5 from natural product. Moreover, we have demonstrated that PAC5 binds hnRNPA2B1 being a new drug target for HBV and SARS-CoV-2 omicron infections. The binding of PAC5 with hnRNPA2B1 activates TBK1-IRF3 signaling, initiates type I IFN production, and promotes the phosphorylation of STAT1/2. It is worthy to mention that PAC5 is currently under preclinical development. Our work showcases the potential of discovering first-in-class drugs based on natural products. Importantly, PAC5 is cheap, non-toxic, and orally available, which holds the promise to be developed as a convenient option for outpatient.

## Materials and Methods

### Mice and reagents

C57BL/6J mice were purchased from the Animal Institute of Southern Medical University (Guangzhou, China). KM mice were purchased from the Animal Institute of Kunming Medical University (Kunming, China). All mice were kept in a pathogen-free environment with the temperature maintained at 21 °C–23 °C and relative humidity at 50%–60% under a 12-h:12-h light: dark cycle. All animal experiments in this study were approved by the Welfare and Ethical Committee for Experimental Animal Care of Southern Medical University and Kunming University of Science and Technology.

3-TC (Lamivudine, PHR1365) and G 418 disulfate salt (A1720) were purchased from Sigma-Aldrich. Anti-hnRNPA2B1 antibody (sc-32316, diluted 1:500) was purchased from Santa Cruz (Santa Cruz, CA, USA). Anti-GAPDH antibody (60004-1-Ig, diluted 1:30,000) was purchased from Proteintech (Chicago, IL, USA). TBK1/NAK rabbit mAb (38066, diluted 1:1000), phospho-TBK1/NAK (Ser172) rabbit mAb (5483, diluted 1:1000), IRF-3 rabbit mAb (4302, diluted 1:1000), and phospho-IRF-3 (Ser396) rabbit mAb (29047, diluted 1:1000) were purchased from Cell Signaling Technology (Danvers, MA, USA). Anti-HBV core antigen/HBcAg antibody (ab8637, diluted 1:500) was purchased from Abcam (Cambridge, UK).

### Cell culture

HepG2.2.15 and HepAD38 cells were purchased from American Type Culture Collection (ATCC) (Rockville, MD, USA) and cultured in high-glucose DMEM (Gibco, Life Technology, Carlsbad, CA, USA) with 10% (v/v) fetal bovine serum (FBS) containing 400 μg/mL G418 in a 5% CO_2_ incubator at 37 °C. HEK293T and HepG2 cells were purchased from ATCC and cultured in high-glucose DMEM supplemented with 10% FBS. Primary human hepatocytes isolated from normal liver tissue and cultured with Power Primary HEP medium (Takara, San Francisco, CA) in the plates coated with 50 μg/mL of rat tail collagen type I (Sigma-Aldrich, St. Louis, MO). The cells was infected with HBV obtained from HepG2.2.15 cells, and the genome copy numbers of intracellular HBV DNA were quantified by a real-time PCR method.

### Preparation and characterization of PAC5, PAC3, and probes

PAC5, PAC3, PAC5- and PAC3-biotin probes, PAC5- and PAC3-RB probes were synthesized by ourselves. Their purity was more than 98.5% by HPLC analysis. Their structures were determined by 1D and 2D NMR, and MS. Synthetic method, NMR data, 1D and 2D NMR, and MS spectra for them are available from the Lead Contact upon request.

### Animal treatments

The method used to establish the HBV-infected mouse model was described previously (Yang et al. 2014). Six- to eight-week-old male C57BL/6 mice were injected with rAAV8-1.3HBV ayw (1 × 10^11^ vg/mouse) dissolved in 200 μL of PBS via the tail vein. rAAV8-1.3HBV ayw was purchased from the Beijing Five plus Molecular Medicine Institute (Beijing, China). The infected mice were assigned to three groups (*n* = 6 per group) and intragastric administration PBS, PAC5 (20 mg/kg), or PAC5 (100 mg/kg) daily, respectively. The experiment was repeated three times independently.

The lamivudine-resistant mouse model of viral hepatitis B was established as previously described (Xia et al. 2020a). Each male C57BL/6J mouse (6–8 weeks old) was hydrodynamically injected with 10 μg of PBSK-rtM204I plasmid (dissolved in sterile PBS equivalent to 8% of the mouse body weight) via the tail vein in 6–8 s. The mice were divided into three groups (*n* = 6 per group) and intra-gastrically administered PBS, 3-TC (50 mg/kg), or PAC5 (100 mg/kg) daily, respectively. The experiment was repeated three times independently.

The treatment was stopped for intra-gastric administration of these drugs in week 8, and the mice were sacrificed in week 10. Serum HBV DNA, HBsAg, and HBeAg levels and hepatic HBcAg expression and hepatic HBV DNA, cccDNA level in the virus-infected mice were detected at the indicated time points. Unless otherwise specified, six mice are normally used in each experimental group.

The golden Syrian hamster model of SARS-CoV-2 infection was established as previous described (Yuan et al. 2021). All experimental protocols were approved by the Animal Ethics Committee in the HKU (CULATR) and were performed according to the standard operating procedures of the biosafety level 3 animal facilities (reference code: CULATR 5370-20). SARS-CoV-2 Omicron (BA.1) were isolated from respiratory tract specimens of laboratory-confirmed COVID-19 patients in Hong Kong. (hCoV-19/Hong Kong/HKU-691/2021; EPI_ISL_7138045). Experimentally, each hamster was intranasally inoculated with 10^5^ PFU of SARS-CoV-2 omicron (BA.1) virus in 100 μL PBS under intraperitoneal ketamine (200 mg/kg) and xylazine (10 mg/kg) anesthesia. Oral administration of PAC5 (100 mg/kg) was performed on 1, 2 and 3 days after infection with the first dosage given at 24 h after inoculation. The hamsters were sacrificed at 4 days after infection for virological and histopathological analyses. Viral yield in the lung tissue homogenates was detected by plaque assay and/or quantitative PCR analysis. Nasal washes were collected to examine virus shedding from the respiratory tract. The expression of IFN-α in the hamster lung tissue was examined by RT-qPCR analysis.

Surrogate mouse model of CoVs lung infection was established using MHV-A59 virus. Six-week-old male C57BL/6 mice were randomly divided into three groups (*n* = 8 per group): uninfected mice (named Ctrl) or MHV-A59-infected mice (named MHV-A59 or MHV-A59+PAC5). MHV-A59-infected mice were intranasally (i.n.) inoculated with the MHV-A59 virus at 1.5 × 10^6^ plaque forming units (PFU) after being given sodium pentobarbital and chloral hydrate for abdominal anesthesia in advance. Meanwhile, the same amount of PBS was intranasally (i.n.) inoculated into Ctrl mice. Mice in the Ctrl and MHV-A59 groups were orally administered PBS daily, and mice in the MHV-A59+PAC5 group were orally administered PAC5 (100 mg/kg) daily after infection. The experiment was repeated three times independently. The health of the mice was monitored daily.

### Determination of HBsAg, HBeAg, HBV DNA, and HBV cccDNA levels

The levels of HBsAg and HBeAg in culture supernatants or mouse serum were measured by chemiluminescence microparticle immunoassay (CMIA) on an Architect system (Abbott Laboratories, North Chicago, IL, USA). A volume of 60 μL of mouse serum was used to extract HBV DNA following the manufacturer’s instructions (Qiagen, Hilden, Germany). Then, real-time PCR was performed with a Roche Cobas Amplicor PCR assay (Roche Molecular Systems, Branchburg, NJ, USA) using SYBR Green Master Mix (commercially available assay kit, TOYOBO, Osaka, Japan).

Intracellular viral nucleocapsid-associated DNA in liver tissues was isolated according to standard genomic DNA isolation methods. Briefly, 20 mg of liver tissues were lysed at 65 °C for 4 h in lysis buffer (50 mmol/L Tris–HCl, pH 8.0, 50 mmol/L EDTA, 100 mmol/L NaCl, 1% SDS) supplemented with proteinase K (200 μg/mL). HBV DNA was extracted by EasyPure Micro Genomic DNA Kit (TransGene Biotech, Beijing, China). The HBV DNA was quantified using specific primers (forward 5ʹ-ACCAATCGCCAGTCAGGAAG-3ʹ and reverse 5ʹ-ACCAGCAGGGAAATACAGGC-3ʹ), by real-time PCR. The digested HBV DNA was inactivated with DNase at 70 °C for 30 min. Primers for cccDNA detection (forward 5ʹ-GTCTGTGCCTTCTCATCTGCC-3ʹ and reverse 5ʹ-ACAGCTTGGAGGCTTGAACAG-3ʹ) were used for real-time PCR.

### Histologic and immunohistochemical staining

Paraffin-embedded liver tissue blocks were cut into 5 μm slices and mounted onto poly-lysine-charged glass slides. Tissue damage was evaluated by H&E staining. Antigen retrieval was performed in 100 °C citrate buffer (pH 6.0) for 10 min, followed by exposure to 3% H_2_O_2_ for 10 min to inhibit endogenous peroxidase activity. The sections were then incubated with primary antibodies at 4 °C overnight. Immuno-reactivity was detected using the corresponding HRP-conjugated secondary antibody and visualized using a diaminobenzidine kit (Beyotime Biotechnology, Beijing, China).

### Measurement of serum ALT, AST and inflammatory cytokines

Serum alanine aminotransferase (ALT) and aspartate transaminase (AST) were measured with commercial kits (Jiancheng Biotech, Nanjing, China) according to the manufacturer’s instructions. The levels of IFN-β in the culture medium and sera were assessed by commercial ELISA kits purchased from Cloud-Clone Corp. (Wuhan, Hubei, China).

### Isolation of RNA and quantitative real-time PCR

Mouse liver total RNA and cellular RNA were extracted using TRIzol reagent (TransGene Biotech, Beijing, China) and then transcribed into cDNA using TranScript All-in-One First-Strand cDNA Synthesis SuperMix (TransGene Biotech) as instructed by the manufacturer. Real-time PCR was performed with a 7900HT Fast Real-time PCR System (Applied Biosystems, San Francisco, CA, USA) using TransStart Tip Green qPCR SuperMix (TransGene Biotech). Expression was normalized to the expression of GAPDH. The primer sequences used in the experiment were as follows: human IFN-β (forward 5ʹ-AGTGTCAGAAGCTCCTGTGGCAA-3ʹ and 5ʹ-ATGCGGCGTCCTCCTTCTGGA-3ʹ), human hnRNPA2B1 (forward 5ʹ-ATTGATGGGAGAGTAGTTGAGCC-3ʹ and reverse 5ʹ-AATTCCGCCAACAAACAGCTT-3ʹ), human GAPDH (forward 5ʹ-GTCTCCTCTGACTTCAACAGCG-3ʹ and reverse 5ʹ-ACCACCCTGTTGCTGTAGCCAA-3ʹ), mouse IFN-β (forward 5ʹ-AGCTCCAAGAAAGGACGAACA-3ʹ and reverse 5ʹ-GCCCTGTAGGTGAGGTTGAT-3ʹ), and mouse GAPDH (forward 5ʹ-CATCACTGCCACCCAGAAGACTG-3ʹ and reverse 5ʹ-ATGCCAGTGAGCTTCCCGTTCAG-3ʹ).

### Immunofluorescence staining

To analyse the colocalization of PAC5 with hnRNPA2B1, cells were plated on a glass coverslip in a six-well plate and then incubated with rhodamine-labeled PAC5. To analyse the subcellular location of hnRNPA2B1, HepG2.2.15, HepG2, and HepAD38 cells were treated with PAC5 or its control analogue for the indicated duration. Cells were fixed with 4% (wt/vol) paraformaldehyde for 15 min and permeabilized with 0.5% (vol/vol) Triton X-100 for another 15 min at room temperature. Subsequently, the cells were blocked in 5% normal goat serum for 1 h at room temperature. The sections were incubated with anti-hnRNPA2B1 (Santa Cruz Biotechnology, diluted 1:100) antibody overnight at 4 °C, followed by incubation with Alexa Fluor 488-labeled secondary antibody for 1 h at room temperature and counterstaining with 4ʹ,6-diamidino-2-phenylindole DAPI (Sigma-Aldrich) for 5 min. Sections were mounted with Mowiol-based antifading medium (Beyotime Biotechnology) and analysed with an LSM 880 confocal microscope (Carl Zeiss, Oberkochen, Germany).

### Plasmids and transient transfection

Plasmids containing Flag-tagged hnRNPA2 (Z4409), Flag-tagged hnRNPB1 (F0171), Myc-tagged IRF3 (Z8111) and HA-tagged TBK1 (S0424) were purchased from GeneCopoeia (Rockville, MD, USA). hnRNPB1-D49A, hnRNPB1-R99A, hnRNPB1-E101A, hnRNPB1-S102A, hnRNPB1-K104A, hnRNPB1-P105A, and hnRNPB1-H108A plasmids were generated from the HNRNPB1 plasmid with D49A, R99A, E101A, S102A, K104A, P105A and H108A point mutations, respectively. HEK293T cells were seeded in 10-cm dishes and co-transfected with 10 μg of plasmids encoding hnRNPA2 and hnRNPB1 mutants with PEI (Polysciences, Warrington, PA, USA) according to the manufacturer’s instructions. HepG2.2.15 cells were plated in six-well plates and transfected with 3 μg of plasmid with PEI according to the manufacturer’s instructions.

### Generation of knockout cells with CRISPR/Cas9

Cells were transfected with 2 μg of IFN-α/βRα (Sc-401662) or 3 μg of hnRNPA2B1 (sc-400635-KO-2) CRISPR/Cas9 KO plasmid (Santa Cruz Biotechnology) using 10 μL of UltraCruz transfection reagent (Santa Cruz Biotechnology) according to the manufacturer’s instructions. 72 h after transfection, the expression levels of hnRNPA2B1 in the cells were assessed by immunoblotting and RT-PCR analysis.

### Protein isolation

A protein extraction kit (Beyotime Biotechnology) was used to extract nuclear and cytoplasmic proteins from 5 × 10^6^ cells in accordance with the manufacturer’s instructions. Briefly, cells were dounce homogenized in a solution containing 10 mmol/L HEPES, 50 mmol/L NaCl, 0.5 mol/L sucrose, 0.1 mmol/L EDTA, and 0.5% Triton X-100. This suspension was spun at 1,000 rpm at 4 °C to pellet the nuclei. The cytoplasmic supernatant was re-spun, and the resulting supernatant was collected. The nuclei were washed in a solution containing 10 mmol/L HEPES, 10 mmol/L KCl, 0.1 mmol/L EDTA, and 0.1 mmol/L EGTA, and the pellets were then suspended in a solution of 10 mmol/L HEPES, 500 mmol/L NaCl, 0.1 mmol/L EDTA, 0.1 mmol/L EGTA, and 0.1% NP-40. This suspension was vortexed and spun to yield a supernatant containing nuclear proteins. The BCA method was used for protein quantification, and then the total protein was boiled for 5 min and stored for preparation.

### Immunoprecipitation and immunoblotting

Cells were lysed in RIPA buffer (50 mmol/L Tris, 150 mmol/L NaCl, and 1% Nonidet P-40, pH 7.4) containing 1 mmol/L phenylmethylsulfonyl fluoride (PMSF) for 30 min. The lysates were ultracentrifuged at 12,000 ×*g* for 15 min at 4 °C. The clear supernatants were added to 2× SDS loading buffer (containing 25 mmol/L Tris-Cl/SDS, pH 6.8, 4.0 g of SDS, 20 mL of glycerol, 1.0 g of bromophenol blue, and 3.1 g of DTT, with H_2_O added to 100 mL) and boiled at 100 °C for 5 min. Protein samples extracted from cells were separated by SDS-PAGE or native PAGE and then transferred onto polyvinylidene fluoride (PVDF) membranes (Millipore, Billerica, MA, USA). After blocking with TBS containing 0.05% Tween-20 and 5% BSA for 1 h at room temperature, the membranes were incubated with primary antibodies at 4 °C overnight or 2 h at room temperature. Subsequently, the membranes were incubated for 1 h at room temperature with horseradish peroxidase (HRP)-conjugated corresponding secondary antibody. For IP, cell lysates were incubated with antibodies (1–2 μg) or immobilized biotinylated PAC5 (30 μmol/L) at 4 °C overnight. Then, 20 µL of protein A/G agarose (Santa Cruz Biotechnology) or 40 µL of avidin agarose beads (Thermo Fisher) was added for 4–6 h at 4 °C. Immunoprecipitates were resuspended in 40 µL of electrophoresis sample buffer, and electrophoresis and immunoblotting were carried out as described above. Finally, the protein of interest was measured with enhanced chemiluminescence (Thermo Fisher) according to the manufacturer’s protocol.

### Pull-down experiments and mass spectrometry analysis

Cells (1 × 10^8^) were lysed in cell lysis buffer (Beyotime Biotechnology) containing 1 mmol/L PMSF. Then, the cell lysates were incubated with immobilized biotinylated PAC5 (30 μmol/L) or NC at 4 °C overnight, followed by incubation with 40 µL of avidin agarose beads (Thermo Fisher) for another 4–6 h. After extensively washing, the immunoprecipitates were eluted by heating to 95 °C in 1% SDS, and the eluted proteins were then subjected to mass spectrometry for protein identification on an AB Sciex TripleTOF 5600+ system (Novogene, China). All MS/MS spectra were searched against the NCBI database using MASCOT (version 2.3).

### RNA extraction and sequencing, quantification of gene expression, and functional enrichment

The HepG2.2.15 cell line was purchased from Kunming Cell Bank (CAS). The cells were cultured in Dulbecco’s modified Eagle’s medium (DMEM), supplemented with 10% fetal calf serum, 100 units/mL penicillin, and 100 mg/mL streptomycin at 37 °C in an atmosphere of 5% CO_2_. The cells were divided into three groups: the blank control group was added with the final concentration of 0.1% DMSO, PAC5 was added with the final concentration of 10 µmol/L as the experimental group, and PAC3 was added with the final concentration of 10 µmol/L as the negative control group. After 48 h co-incubation with cells, RNA of each group was extracted with TRIzol Reagent (Thermo Fisher Scientific). All experiments were repeated three times. The sample purity, concentration and integrity of RNA samples were analysed by NanoPhotometer spectrophotometer (IMPLEN, CA, USA), Qubit3.0 Flurometer (Life Technologies, CA, USA.) and Agilent 2100 RNA Nano 6000 Assay Kit (Agilent Technologies, CA, USA.). Quality libraries are sequenced using the Illumina platform. The sequencing strategy is PE150. Raw reads were subjected to adaptor trimming and filtering of low-quality reads by fastp (v0.21.0) (Chen et al. 2018). Qualified reads were mapped to the human reference genome (GRCh38) using Hisat2 (v2.1.0) (Kim et al. 2019). Gene expression was inferred from the refined BAM files using feature Counts (v1.6.4) (Liao et al. 2014) and are reported as transcripts per million (TPM). Differentially expressed genes (DEGs) were analysed using the R package “limma” (v3.44.3) (Ritchie et al. 2015). Functional enrichment analysis was performed using the function “GSEA” in the R package clusterProfiler (Yu et al. 2012).

### Pocket identification and molecular docking

The protein structure of hnRNPA2B1 (ID: 5HO4) was obtained from the PDB database. The potential binding pockets of hnRNPA2B1 were identified by Fpocket (v3) with the default settings (Le Guilloux et al. 2009). Then, the interactions between PAC5 and these pockets in hnRNPA2B1 were predicted by AutoDock Vina (v1.1.2) (Trott and Olson 2010). The structures of hnRNPA2B1 (ID: 5HO4) and PAC5 were prepared with AutoDock tools v1.5.6 as suggested in the user guide. The pocket center was set as the docking center. To allow free rotation of the compounds, the search space was set to 30 × 30 × 30 Å in each axis. The molecular input was in pdbqt format. All other docking parameters were set to the default values. Each docking experiment was performed with a command that contained space size and the three-dimensional coordinates of the docking center. A lower energy score indicated a stronger binding affinity between the ligand and receptor. The binding pose with the lowest energy was selected as the best model for each docking experiment. The interaction between PAC5 and hnRNPA2B1 was displayed using the PyMOL (v1.7) program.

### Statistical analysis

The experimental data are presented as the mean ± SD. One-way ANOVA with Bonferroni post hoc test was used for comparisons among multiple groups. Two-way ANOVA with Bonferroni post hoc test was used for comparisons among multiple groups with two categorical independent variables. Experimental differences between two groups were analysed by Student’s *t*-test. The data were considered statistically significant when the *P*-value was < 0.05.

## Supplementary Material

pwac027_suppl_Supplementary_Materials

## Abbreviations

ABPPs: activity-based protein profilings
ALT: serum alanine aminotransferase
AST: aspartate transaminase
cccDNA: covalently closed circular DNA
CMIA: chemiluminescence microparticle immunoassay
CoV: coronavirus
DAAs: direct-acting antivirals
DEGs: differentially expressed genes
GSEA: Gene set enrichment analysis
HBeAg: hepatitis B e antigen
HBsAg: hepatitis B surface antigen
HBV: hepatitis B virus
HDAs: host-directed antivirals
hnRNPA2B1: heterogeneous nuclear ribonucleoprotein A2B1
IFNα/βR: IFN-α/β receptor
IP: immunoprecipitation
IRF3: IFN regulatory factor 3
ISGs: IFN-stimulated genes
m6A: N6-methyladenosine
MHV: mouse hepatitis virus
Mpro: main protease
PA: phyllanthacidoid A
PFU: plaque forming units
RB: rhodamine B
RRM: RNA/DNA-recognition motifs
SARS-CoV-2: severe acute respiratory syndrome coronavirus 2
TBK1: TANK-binding kinase 1
TMPRSS2: transmembrane serine protease 2
TPM: transcripts per million

## Declarations

This work was supported by the National Natural Science Foundation of China (Grant Nos. 31960093, 81973210, 81873872, 82071781, 32160153), the Natural Science Foundation of Yunnan Province (Grant Nos. 202001BC070001, 202102AA100053, 202105AD160008, and 202207AA110003), and the Innovation Team of Chronic Kidney Disease with Integrated Traditional Chinese and Western Medicine (No. 2019KCXTD014). We also thank the Program for Innovative Research Team (in Science and Technology) at the University of Yunnan Province and Ronald J Quinn AM Academician Workstation (No. 2019IC003). This study was partly supported by funding to The University of Hong Kong: the Health and Medical Research Fund (No. 20190732) and Hong Kong General Research Fund (No. HKU171260/17M). The funding sources had no role in the study design, data collection, analysis, interpretation or writing of the report.

All institutional and national guidelines for the care and use of laboratory animals were followed. All experimental protocols related to SARS-CoV-2 were approved by the Animal Ethics Committee in the HKU (CULATR) and were performed according to the standard operating procedures of the biosafety level 3 animal facilities (reference code: CULATR 5370-20). There was no human subject participated in this project.

The sequencing data of transcriptomics in this paper have been deposited in the Genome Sequence Archive in National Genomics Data Center, Chinese Academy of Sciences, under accession number HRA001695 that are publicly accessible at the NGDC database. The MS/MS spectra data of proteomic is available in the OMIX, China National Center for Bioinformation/ Beijing Institute of Genomics, Chinese Academy of Sciences (NGDC database: accession no. OMIX811). Any additional information required to reanalyze the data reported in this work paper is available from the Lead Contact upon request.

Biological experiments and collected the data: DMZ, YC, JPC, YF, LG, SFY; Chemical experiments: HYY, JQW, JL, MX; Design of the bioinformatics data: SXD, XT; Funding acquisition: MX, DMZ, SXD, SFY; Project administration: MX; Supervision: MX, DMZ, SXD, SFY; Writing-original draft: MX, DMZ, SXD, SFY; Writing-review and editing: MX, DMZ, SXD, SF, RJQ, SJZ.
