# Supplementary material for: A hnRNPA2B1 agonist effectively inhibits HBV and SARS-CoV-2 omicron in vivo
Source: Protein Cell. 2022 Jul 15;14(1):37–50. doi: 10.1093/procel/pwac027 (PMC9871967; doi:10.1093/procel/pwac027)
Supplement: pwac027_suppl_Supplementary_Materials [file pwac027_suppl_supplementary_materials.docx]

**Supplemental materials for**

**A hnRNPA2B1 agonist effectively inhibits HBV and SARS-CoV-2 Omicron *in vivo***

Daming Zuo^2,9,10*^, Yu Chen^2,10^, Jian-piao Cai^4,10^, Hao-Yang Yuan1, Jun-Qi Wu1, Yue Yin^2^, Jing-Wen Xie^2^, Jing-Min Lin^2^, Jia Luo1, Yang Feng^1^, Long-Jiao Ge^3^, Jia Zhou^5^, Ronald J. Quinn^6^, San-Jun Zhao^7^, Xing Tong^3^, Dong-Yan Jin^8^, Shuofeng Yuan^4,*^, Shao-Xing Dai^3,*^, Min Xu1^,*^

^1^ Center for Pharmaceutical Sciences, Faculty of Life Science and Technology, Kunming University of Science and Technology, Chenggong Campus, Kunming 650500, China

^2^ Department of Medical Laboratory, School of Laboratory Medicine and Biotechnology, Southern Medical University, Guangdong 510515, China

^3^ State Key Laboratory of Primate Biomedical Research; Institute of Primate Translational Medicine, Kunming University of Science and Technology, Kunming 650500, China

^4^ Department of Microbiology, Li Ka Shing Faculty of Medicine, The University of Hong Kong, Pokfulam, Hong Kong SAR, China.

^5^ Department of Immunology, School of Basic Medical Sciences, Southern Medical University, Guangdong 510515, China

^6^ Griffith Institute for Drug Discovery, Griffith University, Brisbane, QLD 4111, Australia

^7^ School of Life Sciences, Yunnan Normal University, Kunming 650500, China

^8^ School of Biomedical Sciences, Li Ka Shing Faculty of Medicine, The University of Hong Kong, Pokfulam, Hong Kong SAR, China.

^9^ Microbiome Medicine Center, Department of Laboratory medicine, Zhujiang Hospital, Southern Medical University, Guangdong 510515, China

^10^ These authors contributed equally to this work.

* Corresponding author. e-mail: zdaming@smu.edu.cn, [yuansf@hku.hk](mailto:yuansf@hku.hk), daishaoxing@kust.edu.cn, xumin@kust.edu.cn

Supplementary Table 1. Physicochemical parameter and anti-HBV activity of PAC5 derivatives

Physicochemical parameter estimated by ChemBioOffice 2014. PAC5 derivatives inhibited levels of HBsAg and HBeAg in the supernatant of HepG2.2.15 cells, HepG2 cells (transfected PBSK-rtM204I), and HepG2 cells (without infection).

| **Compounds** | | **Physicochemical parameter^a^** | | | | | **IC_50_ (*µ*M)^c^** | | | **CC_50_ (*µ*M)^d^** | | **SI^e^** |
| --- | --- | --- | --- | --- | --- | --- | --- | --- | --- | --- | --- | --- |
|  | | Mw | | ClogP | tPSA | | HBsAg | | HBeAg |  | |  |
| **HepG2.2.15** | | | | | | | | | | | | |
| **PA** | XM-1 | 746.71 | | -1.57 | 312.05 | | 14.96±0.5 | | 95.99±27.2 | 19.2 | | 1.5 |
| **1a** | XM-2 | 438.43 | | 0.54 | 162.98 | | NA^f^ | | NA | NT^g^ | | **-** |
| **1b** | XM-3 | 318.32 | | -2.21 | 136.68 | | NA | | NA | NT | | **-** |
| **1c** | HY-9 | 776.47 | | 6.52 | 140.98 | | NA | | NA | NT | | **-** |
| **1d** | HY-2 | 508.56 | | 3.09 | 107.98 | | NA | | NA | NT | | **-** |
| **1e** | HY-3 | 388.46 | | 0.75 | 81.68 | | NA | | NA | NT | | **-** |
| **2a** | - | 452.46 | | 0.99 | 151.98 | | NA | | NA | NT | | **-** |
| **2b** | HY-31 | 480.51 | | 2.05 | 151.98 | | 106.89±28.3 | | 131.28±29.5 | NT | | **-** |
| **2c** | HY-32 | 494.54 | | 2.57 | 151.98 | | 0.03±0.01 | | 83.06±6.28 | > 30 | | >1000 |
| **2d** | HY-34 | 508.56 | | 3.10 | 151.98 | | 0.1±0.01 | | 106.45±36.9 | > 30 | | >300 |
| **2e** | HY-22 | 558.57 | | 2.25 | 140.98 | | 139.8±3.7 | | NA | > 30 | | >0.21 |
| **2f** | HY-36 | 586.63 | | 3.01 | 140.98 | | 141±3.6 | | 51.22±9.2 | > 30 | | >0.21 |
| **2g** | HY-23 | 614.68 | | 4.07 | 140.98 | | NA | | NA | NT | | - |
| **3a** | HY-25 | 465.5 | | 0.51 | 154.78 | | 155.52±1.1 | | NA | NT | | **-** |
| **3b (PAC3)** | HY-26 | 479.53 | | 1.04 | 154.78 | | 170.74±0.5 | | NA | NT | | - |
| **3c** | HY-30 | 493.55 | | 1.57 | 154.78 | | 44.46±3.1 | | 175.9±9.86 | NT | | **-** |
| **3d（PAC5）** | **HY-16** | **507.58** | | **2.10** | **154.78** | | **0.81±0.9** | | **5.88±0.8** | **> 900** | | **>1100** |
| **3e** | HY-4 | 521.61 | | 2.62 | 154.78 | | 15.97±21.77 | | 65.47±10.21 | > 30 | | >1.88 |
| **3f** | HY-15 | 535.63 | | 3.15 | 154.78 | | 8.42±0.9 | | 33.09±0.4 | > 30 | | >3.56 |
| **3g** | HY-7 | 549.66 | | 3.68 | 154.78 | | 16.91±9.05 | | 24.68±0.4 | > 30 | | >1.77 |
| **3h** | HY-5 | 523.58 | | 1.06 | 164.01 | | 31.47±7.07 | | 277.2±4.68 | NT | | **-** |
| **3i** | HY-17 | 541.6 | | 2.08 | 154.78 | | 13.91±0.9 | | 82.19±5.7 | > 30 | | >2.15 |
| **3j** | HY-10 | 601.65 | | 1.73 | 173.24 | | NA | | NA | NT | | **-** |
| **4a** | HY-1 | 565.62 | | 1.05 | 181.08 | | NA | | NA | NT | | **-** |
| **4b** | HY-14 | 593.67 | | 1.89 | 181.08 | | 49.21±6.6 | | NA | > 30 | | >0.61 |
| **4c** | HY-13 | 607.7 | | 2.64 | 181.08 | | 70.13±14.7 | | NA | > 30 | | >0.43 |
| **4d** | HY-39 | 621.72 | | 2.95 | 181.08 | | 3.83±0.5 | | 51.99±5.1 | > 30 | |  |
| **4e** | HY-40 | 635.75 | | 3.35 | 181.08 | | 70.42±3.8 | | 102.6±7.07 | NT | | **-** |
| **4f** | HY-27 | 593.67 | | 2.11 | 181.08 | | 169.79±1 | | NA | > 30 | | >0.18 |
| **4g** | HY-24 | 621.72 | | 3.17 | 181.08 | | NA | | NA | NT | | **-** |
| **4h** | HY-33 | 593.67 | | 2.11 | 181.08 | | 23.11±2.5 | | NA | NT | | **-** |
| **4i** | HY-21 | 607.7 | | 2.64 | 181.08 | | 133.52±2.6 | | NA | NT | | **-** |
| **4j** | HY-28 | 621.72 | | 2.95 | 181.08 | | 134.58±0.6 | | NA | NT | | **-** |
| **4k** | HY-29 | 635.75 | | 3.70 | 181.08 | | NA | | NA | NT | | **-** |
| **4l** | HY-37 | 635.75 | | 3.48 | 181.08 | | 95.37±10.6 | | 97.26±19.6 | NT | | **-** |
| **4m** | HY-38 | 649.78 | | 3.88 | 181.08 | | 81.88±3.7 | | 65.68±5.6 | NT | | **-** |
| **4n** | HY-12 | 678.78 | | 1.85 | 210.18 | | 118.508±18.4 | | NA | NT | | **-** |
| **4o** | HY-18 | 664.75 | | 1.32 | 210.18 | | NA | | NA | NT | | **-** |
| **4p** | HY-19 | 740.85 | | 2.87 | 210.18 | | NA | | NA | NT | | **-** |
| **3AT** inhibition (%) | - | - | | - | - | | 44.6±2.1 (100 *µ*M) | | 26.0±9.6 (100 *µ*M) | NT | | **-** |
| **Probe of PAC5** | LJ1-26-1 | - | | - | - | | 0.55±0.1 | | 0.97 ±0.01 | NT | | **-** |
| **Probe of PAC3** | LJ1-66-1 | - | | - | - | | 164.6±3.47 | | NA | NT | | **-** |
| **Blank Probe** | LJ1-76-1 | - | | - | - | | 101.33±16.8 | | NA | NT | | **-** |
| **HepG2 cells (transfected PBSK-rtM204I)** | | | | | | | | | | | | |
| **3d（PAC5）** | **HY-16** | **507.58** | | **2.10** | **154.78** | | **2.11±0.01** | | **2.64±3.8** | **NT** | | **-** |
| **Probe of PAC5** | LJ1-26-1 | - | | - | - | | 0.37±0.01 | | 21.65±4.41 | NT | | **-** |
| **Probe of PAC3** | LJ1-66-1 | - | | - | - | | >100 | | NA | NT | | **-** |
| **Blank Probe** | LJ1-76-1 | - | | - | - | | >100 | | NA | NT | | **-** |
| **HepG2 cells (without infection)** | | | | | | | | | | | | |
| **3d（PAC5）** | **HY-16** | **507.58** | | **2.10** | **154.78** | | **-** | | **-** | **>500** | | **-** |
| ^a^ In silico calculations performed using Chemdraw 14.0 software. Molecule weight (Da), ClogP, %PSA = % polar surface area; | | | | | | | | | | |  |  |
| ^b.^ Lamivuding (3AT) was tested as the positive control of anti-HBV; | | | | | | |  | |  |  | |  |
| ^c.^ Compound concentration inhibiting HBsAg or HBeAg; | | | | | |  |  | |  |  | |  |
| ^d.^ Compound concentration reducing the viability of HepG2. cells culture by 50%; | | | | | | | |  |  |  | |  |
| ^e.^ Selective index, SI = CC_50_/IC_50_ | | |  |  |  | |  | |  |  | |  |
| ^f.^ Not active below 200 µM. | |  | |  |  | |  | |  |  | |  |
| ^g.^ No tested. |  |  | |  |  | |  | |  |  | |  |

Supplementary Table 2. *In vivo* toxicity study of PAC5 in KM mouse.

KM mice (6-8 weeks) were weighted and assigned to 2 groups and o.p. administration twice daily with PBS and PAC5 (2 g/kg), respectively. Subsequently, body weight was recorded once a day until the 14th day, as well as skin, eyes, breathing, movement, mental state, mucous membranes, excrement of mice.

|  | Administration | Dosage  (mg/Kg) | Frequency | Weight of mouse (g) | | | | | | | | | | | | | | |
| --- | --- | --- | --- | --- | --- | --- | --- | --- | --- | --- | --- | --- | --- | --- | --- | --- | --- | --- |
|  |  |  |  | Day 0 | Day 1 | Day 2 | Day 3 | Day 4 | Day 5 | Day 6 | Day 7 | Day 8 | Day 9 | Day 10 | Day 11 | Day 12 | Day 13 | Day 14 |
|  | o.p. | 0 | Twice daily | 25 | 26.5 | 28.5 | 27.5 | 28.5 | 28 | 29.5 | 29.5 | 29.5 | 29 | 30 | 31 | 29.5 | 30.5 | 31 |
|  | o.p. | 0 | Twice daily | 29.5 | 29.5 | 30 | 29 | 30 | 30 | 30 | 30 | 30 | 30.5 | 31 | 30.5 | 30.5 | 30 | 30 |
| Ave ± SD |  |  |  | 27.25±3.18 | 28±2.12 | 29.25±1.06 | 28.25±1.06 | 29.25±1.06 | 29±1.41 | 29.75±0.35 | 29.75±0.35 | 29.75±0.35 | 29.75±1.06 | 30.5±0.71 | 30.75±0.35 | 30±0.71 | 30.25±0.35 | 30.5±0.71 |
|  | o.p. | 2000 | Twice daily | 24.5 | 26 | 26.5 | 26 | 24.5 | 26 | 27 | 27.5 | 27 | 27.5 | 28.5 | 29 | 28.5 | 28 | 28.5 |
|  | o.p. | 2000 | Twice daily | 31 | 31 | 32 | 33 | 33 | 33 | 33.5 | 33 | 32.5 | 33 | 34 | 34 | 33.5 | 34 | 33.5 |
|  | o.p. | 2000 | Twice daily | 27.5 | 28.5 | 28.5 | 29 | 28.5 | 28.5 | 28.5 | 28.5 | 28 | 27.5 | 29.5 | 28.5 | 28.5 | 29 | 29 |
|  | o.p. | 2000 | Twice daily | 27 | 25 | 24.5 | 24.5 | 26 | 26 | 27 | 27.5 | 26.5 | 27 | 27.5 | 27.5 | 28.5 | 28.5 | 27.5 |
|  | o.p. | 2000 | Twice daily | 31 | 32 | 33 | 32.5 | 32.5 | 33 | 33.5 | 34 | 33.5 | 34 | 35 | 35.5 | 34 | 34 | 35.5 |
| Ave ± SD |  |  |  | 28.2±2.80 | 28.5±3.04 | 28.9±3.60 | 29±3.79 | 28.9±3.79 | 29.3±3.53 | 29.9±3.34 | 30.1±3.15 | 29.5±3.26 | 29.8±3.40 | 30.9±3.38 | 30.9±3.59 | 30.6±2.88 | 30.7±3.03 | 30.8±3.49 |


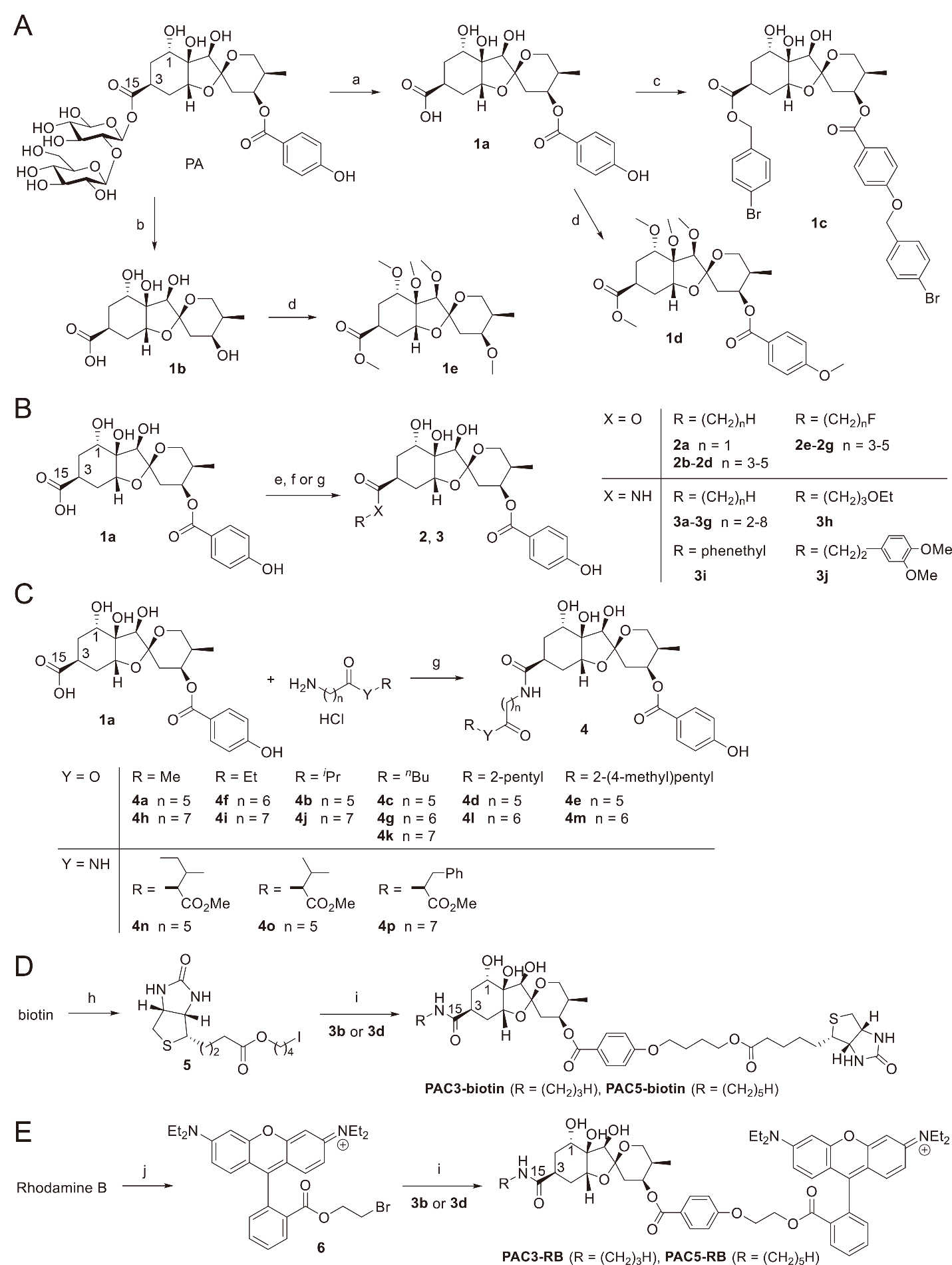
Supplementary figure 1. Synthesis of PA derivatives

(**A**) Synthesis of compounds **1A**-**1E**; (**B**) Synthesis of esters **2** and amides **3**, PAC5 is **3b** and PAC3 is **3d**; (**C**) Synthesis of amides **4**; (**D**) Synthesis of biotin probes; (**E**) Synthesis of rhodamine B probes. Reagents and conditions: (A) aq. K_2_CO_3_, 60 ^o^C, 2 h; (B) aq. NaOH, 70 ^o^C, 2 h; (C) (4-BrPh)CH_2_Br, K_2_CO_3_, DMF, rt, overnight; (D) CH_3_I, NaH, DMF, rt, overnight; (E) alcohol, EDCI, DMAP, DMF, 12h; (F) Br(CH_2_)_n_F, K_2_CO_3_, DMF, rt, overnight; (G) amine, EDCI, HOSu, DMAP, DMF, rt, overnight; (H) I(CH_2_)_4_I, K_2_CO_3_, DMF, rt, 12 h; (I) **3b** or **3d**, K_2_CO_3_, DMF, rt, 12 h; (J) BrCH_2_CH_2_OH, EDCI, DMAP, DMF, 45 ^o^C, 5 h.


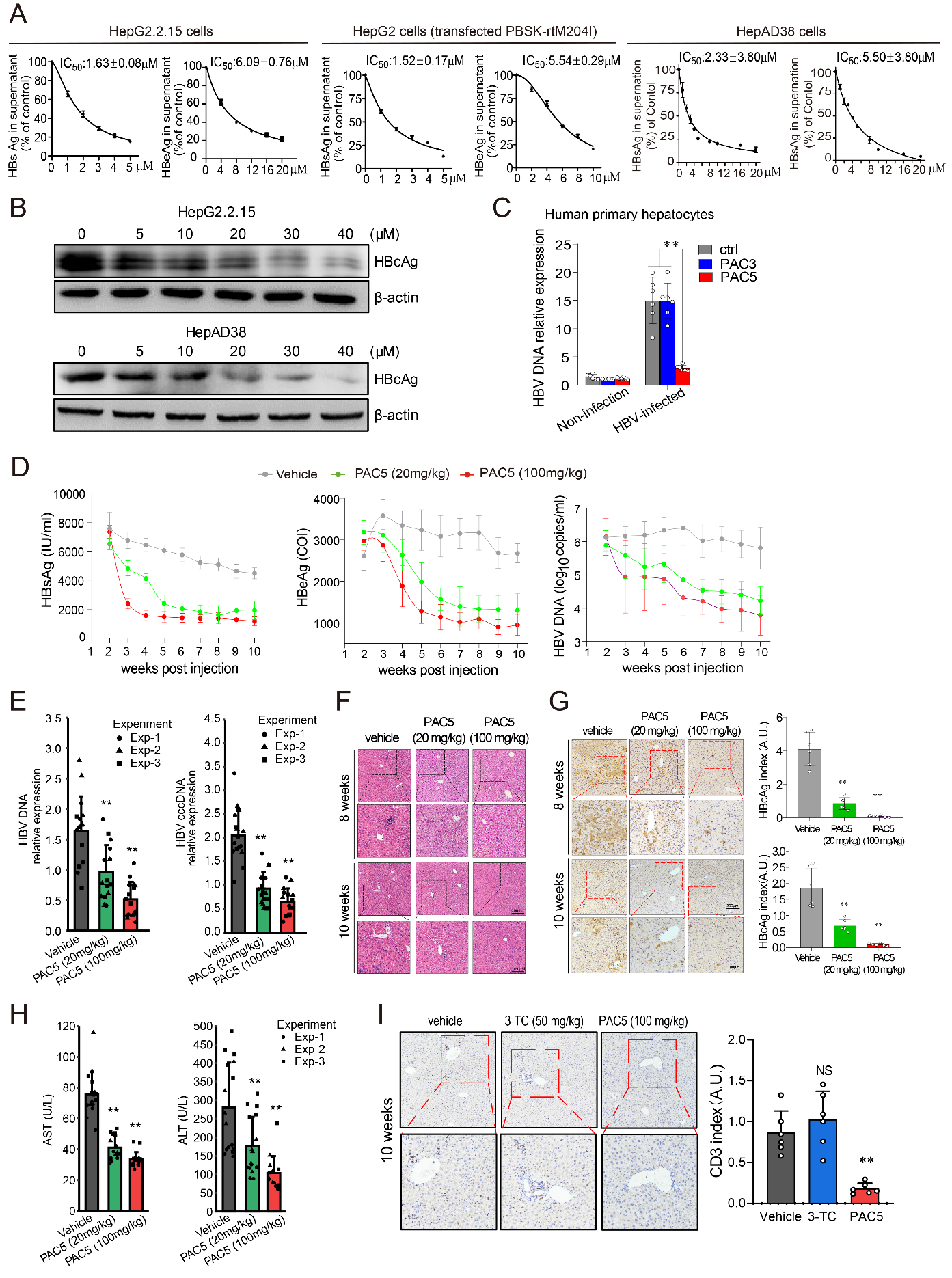


Supplementary figure 2. PAC5 inhibits HBV replication *in vitro* and *vivo*.

**(A)** PAC5 inhibited levels of HBsAg and HBeAg in the supernatant of HepG2.2.2.15 cells, HepG2 cells transfected PBSk-rtM2041, and HepAD38 cells. **(B)** The protein levels of HBcAg in HepG2.2.2.15 and HepAD38 cells were evaluated by immunoblotting. **(C)** Primary human hepatocytes were infected with HBV virus in the presence of 2 μM PAC5 for 24 hours, and the copy numbers of intracellular HBV DNA were quantified by a real-time PCR method. (**D**-**I)**, After 2 weeks of intravenous injection of rAAV8-1.3HBV ayw (1×10^11^ Vg of each/mouse), C57BL/6 mice were assigned to 3 groups (n = 6 per group) and intragastric administration daily with PBS, PAC5 (20 mg/kg), and PAC5 (100 mg/kg), respectively. The treatment was stopped at week 8, and all the mice were sacrificed at week 10. **(D)** The serum levels of HBeAg and HBsAg were monitored by CMIA, and the HBV DNA level was detected by real-time PCR analysis. **(E)** The expression of HBV DNA and HBV cccDNA in the liver tissues was detected by quantitative RT-PCR analysis. **(F)** The histologic analysis of livers was performed using H&E staining. **(G)** HBcAg expression in the liver tissues was determined by immunohistochemical staining. **(H)** The sera ALT and AST activities were detected at week 10. **(I)** The hepatic infiltration of T lymphocytes was determined by immunohistochemical staining with anti-CD3 antibody. Three independent experiments were performed. All data were presented as means ± SD. *p< 0.05, **p<0.01, calculated by one-way ANOVA with Bonferroni post hoc test (E, G, H, I) and two-way ANOVA with Bonferroni post hoc test (C), respectively. One representative experiment was showed.


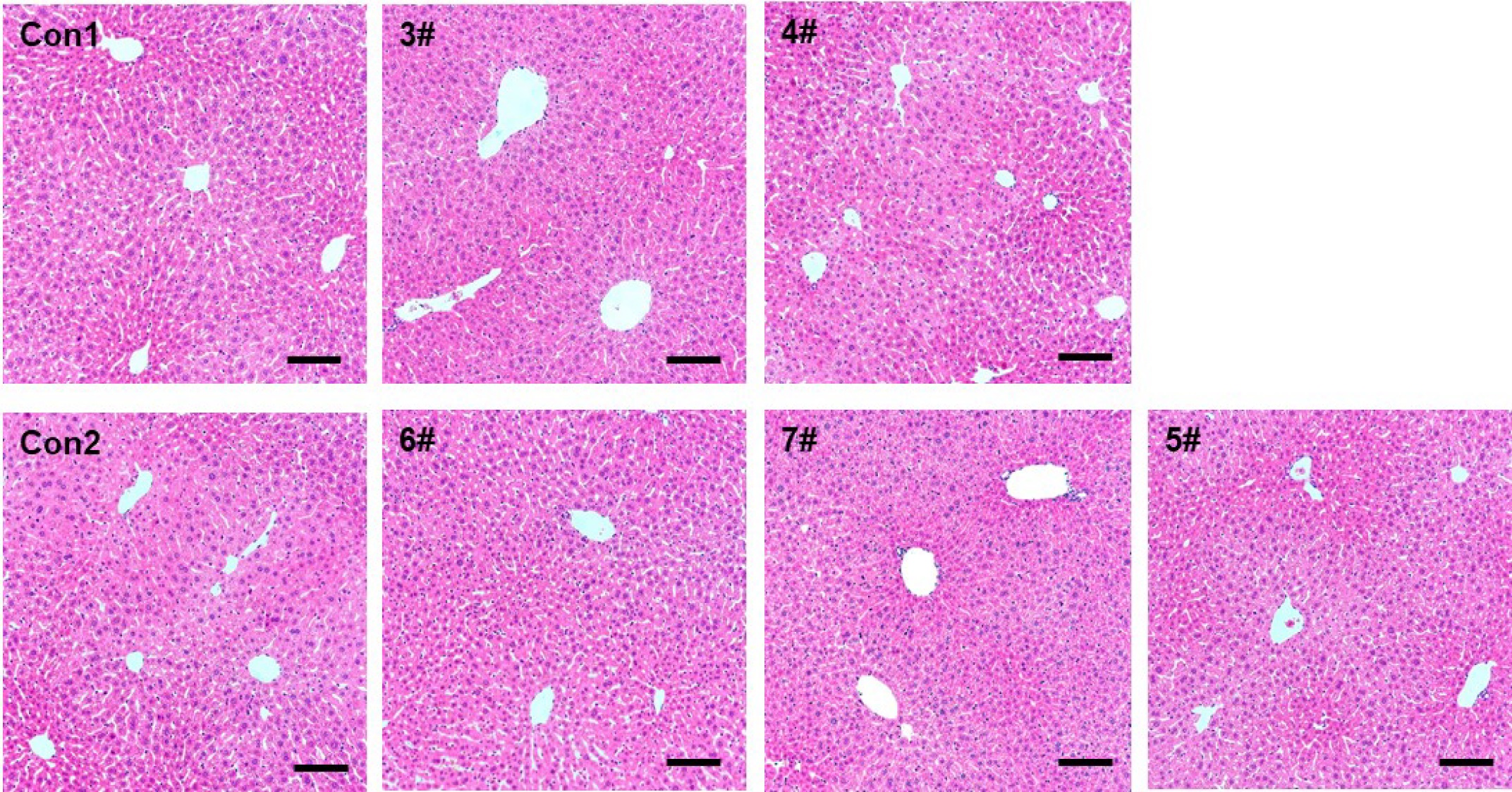


Supplementary figure 3. Evaluation of *in vivo* safety of PAC5 in liver of KM mice without infection. Two KM mice (Con1 and Con2) and five KM mice (#3-7) were intragastric administrated with PBS, and PAC5 (2000 mg/kg dose), respectively. After 24 hours of intragastric administration, all the mice were sacrificed for the histologic analysis of livers using H&E staining. The scale bar is 50 μm.


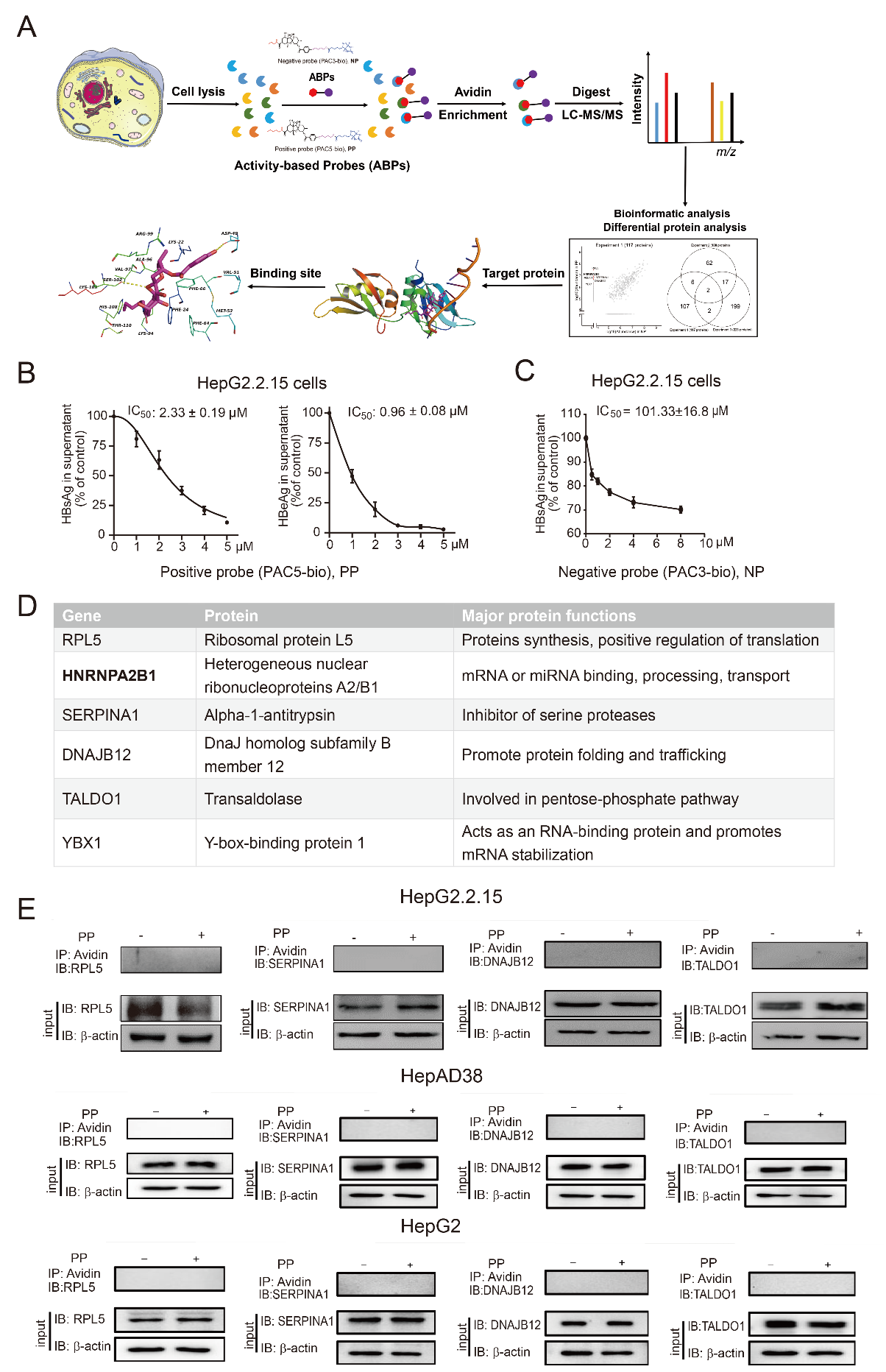


**Supplementary figure 4. Anti-HBV activity of probes and other validated by co-precipitation assay**

(A) The diagrammatic sketch of activity-based protein profilings (ABPPs). (B) The levels of HBsAg and HBeAg in the supernatant of HepG2.2.15 cells cultured with positive probe (PP, PAC5-bio) were measured by CMIA. (C) The levels of HBsAg in the supernatant of HepG2.2.15 cells with negative probe (NP, PAC3-bio) were measured by CMIA at indicated time points. (D) The top five differential expressed proteins and common protein (YBX1) with their functions were listed. (E) The interaction of PAC5 with the candidate proteins was validated by co-precipitation assay in HepG2.2.15, HepG2 and HepAD38 cells. Three independent experiments were performed. All data were presented as means ± SD. One representative experiment was showed.


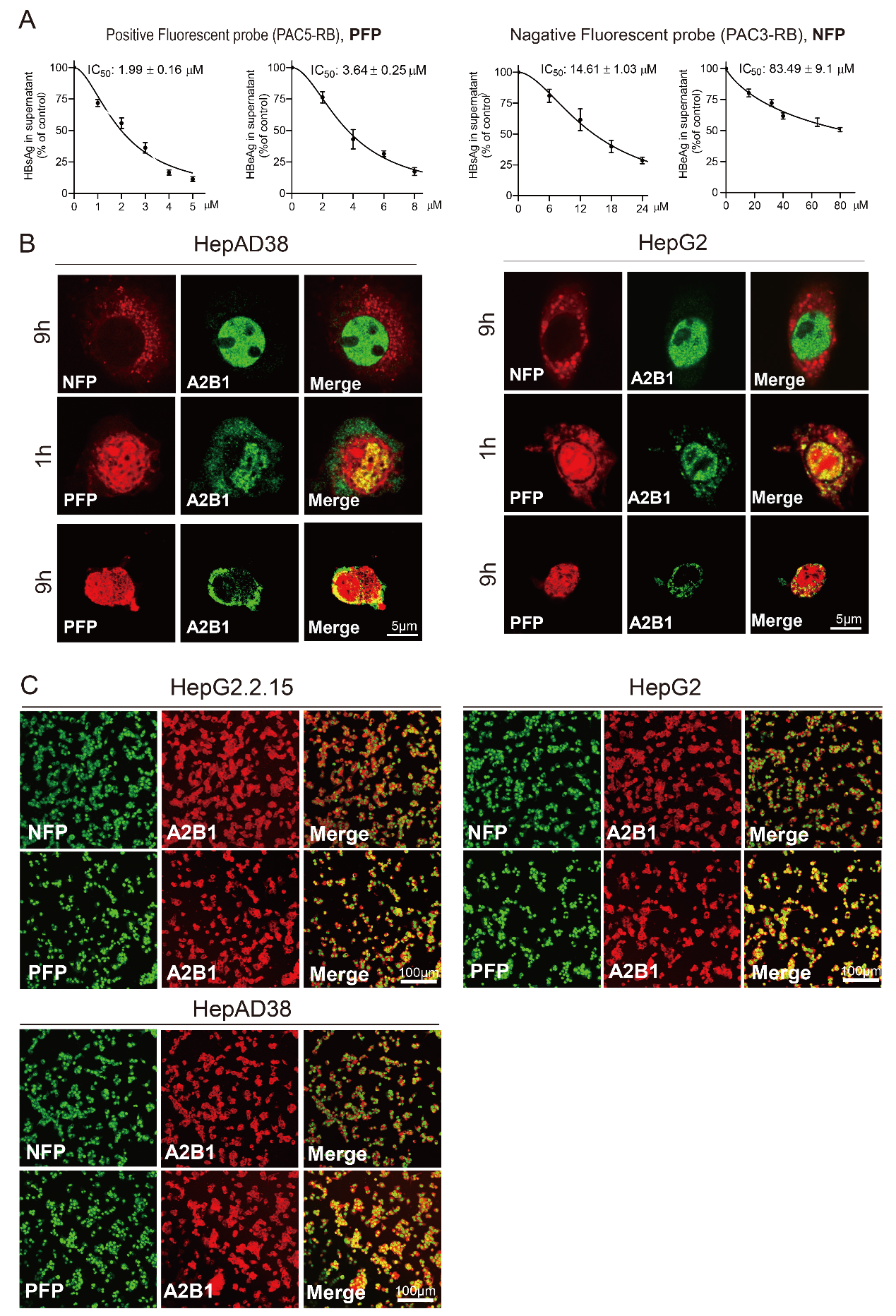


**Supplementary figure 5. Anti-HBV activity of Fluorescent Probes and the colocalization of PAC5 and hnRNPA2B1**

**(A)** The levels of HBsAg and HBeAg in the supernatant of HepG2.2.15 cells cultured with positive fluorescent probe PAC5-RB (PFP) and negative fluorescent probe PAC3-RB (NFP) were measured by CMIA. **(B)** The colocalization of PFP (red) and hnRNPA2B1 (green) was determined by confocal assay in HepG2 and HepAD38 cells. One representative experiment was showed. **(C)** The colocalization of PFP (red) and hnRNPA2B1 (green) in the three cell lines was shown with Multicellular view field. Three independent experiments were performed. All data were presented as means ± SD. One representative experiment was showed.


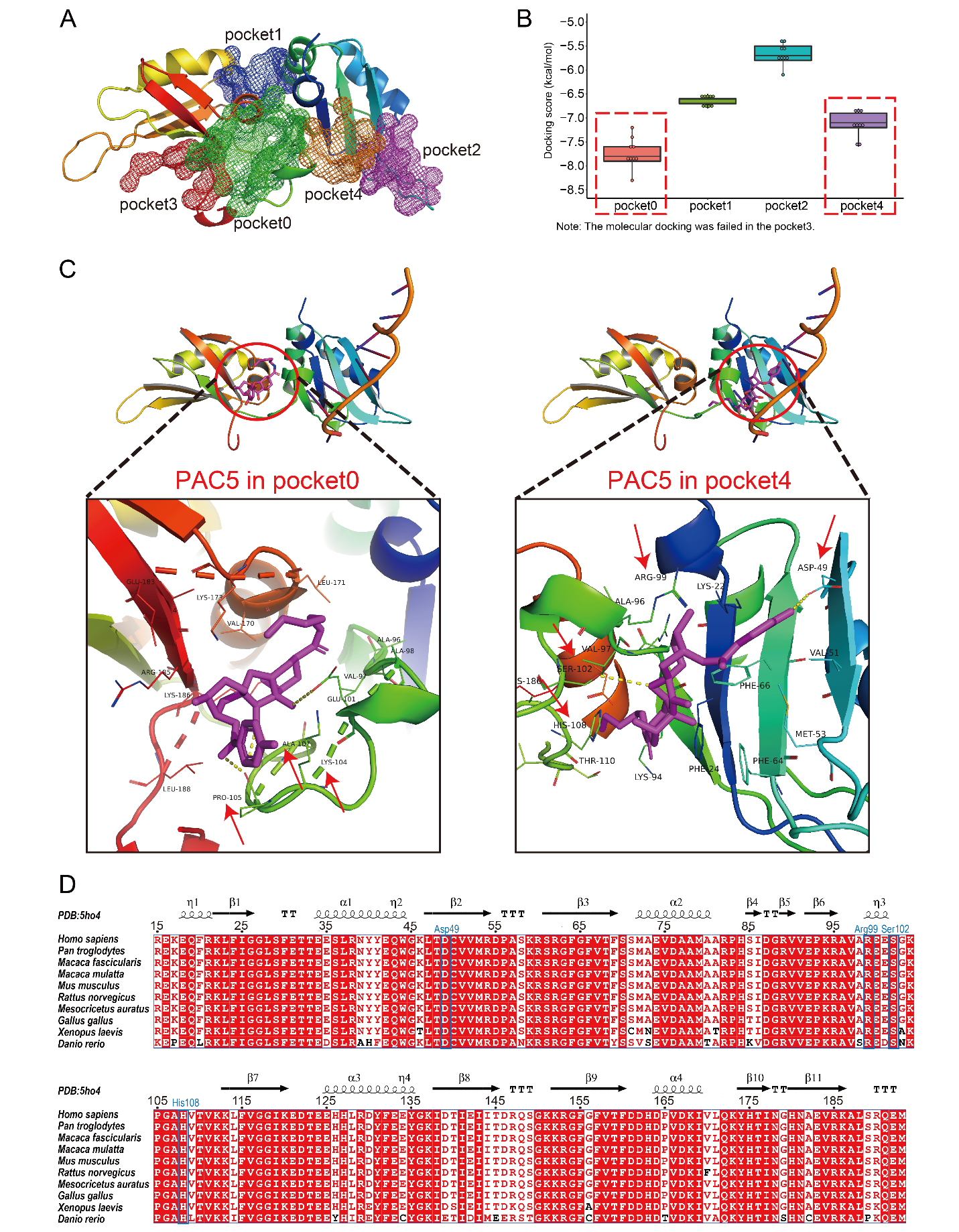


**Supplementary figure 6. Pocket identification, molecular docking, the binding of PAC to pocket 0 and 4, and sequence alignment of hnRNPA2B1**

**(A)** The potential pockets identified by Fpocket were distributed at the structure of hnRNPA2B1. Pockets were shown as mesh with different color. Structure of hnRNPA2B1 was shown as cartoon. **(B)** the boxplot showed the docking score of PAC5 to different pockets. The pocket 0 and pocket 4 are highlighted as relative favorable pocket for PAC5. **(C)** (Top panel) the overview of Compound PAC5 binding to the pocket 0 and 4, respectively. (Bottom panel) Close-up view of the binding of compound PAC5 to the pocket 0 and 4 of hnRNPA2B1, respectively. The side chains of the residues involved in binding are labeled and shown as sticks. The compound PAC5 was shown as magenta sticks. Secondary structural elements were colored from blue (N-terminus) to red (C-terminus). The residues involved point mutation experiment were marked with arrows. The pocket 0 and 4 were highlighted with red circles. **(D)** The high conservation of hnRNPA2B1 among mammals revealed by sequence alignment. The aligned hnRNPA2B1 sequences are from species include *Homo sapiens*, the experimental animals *Mus musculus* (mouse) and *Mesocricetus auratus* (golden Syrian hamster) in this study, and other mammals. The hnRNPA2B1 sequences from *Gallus gallus, Xenopus laevis, Danio rerio* are used outgroup. The structure of hnRNPA2B1 (PDB:5ho4) was used to show secondary structure generated by ESPript (<https://espript.ibcp.fr>). The binding site of PAC5 were highlighted with blue rectangle.


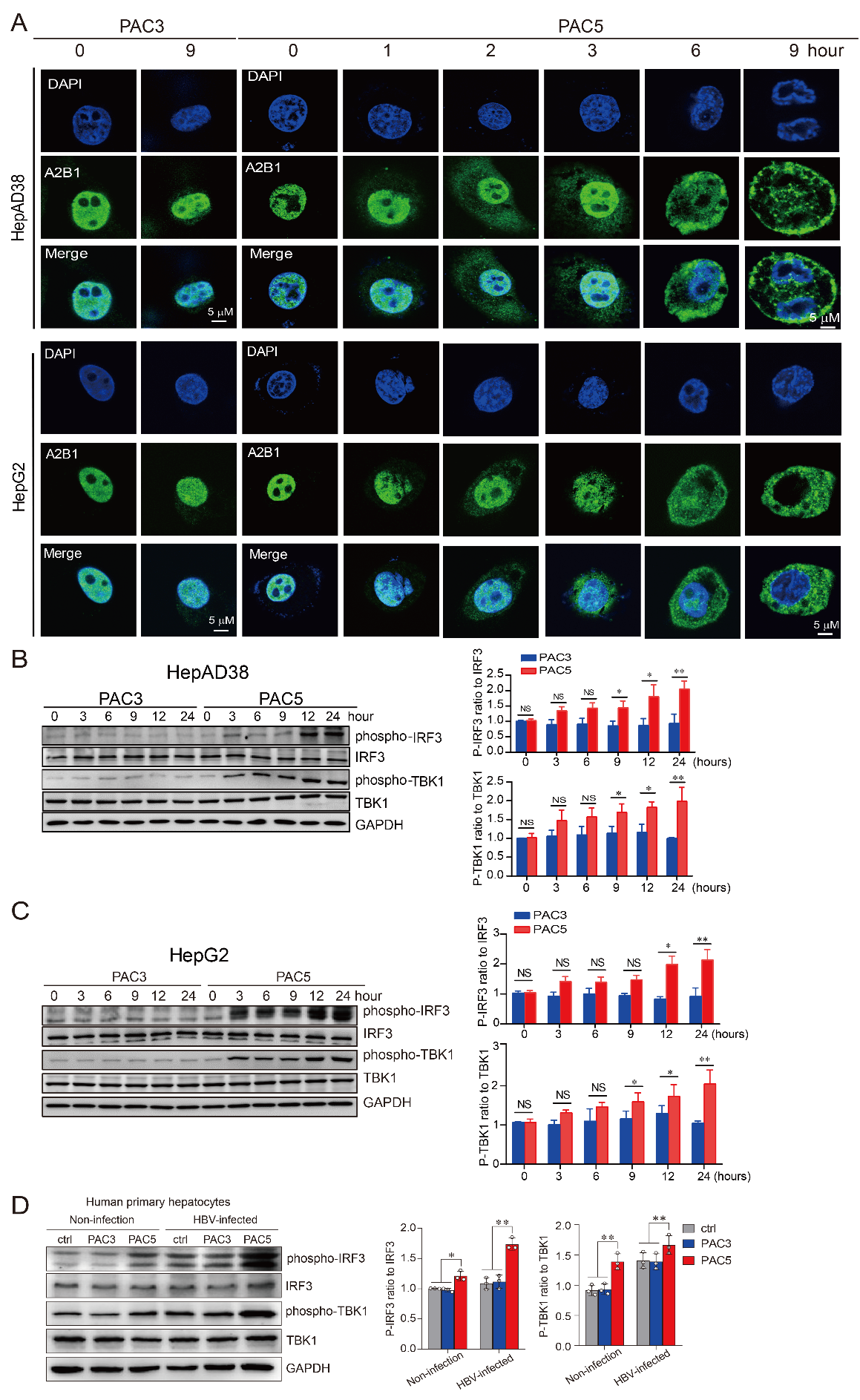


**Supplementary figure 7. Intracellular localization of hnRNPA2B1 and phospho-TBK1 and IRF3 in HepG2 and HepAD38 cells**

**(A)** HepG2 and HepAD38 cells were treated with PAC3 (2 μM) or PAC5 (2 μM) for indicated time points. The intracellular localization of hnRNPA2B1 (green) was determined by confocal microscopy. The nuclei are stained with DAPI (blue). **(B)** HepAD38 cells were treated with PAC5 (2 μM) or PAC3 (2 μM) for the indicated time. Phosphorylated (phospho-) TBK1 and IRF3 were detected by immunoblotting assay. **(C)** HepG2 cells were treated with PAC5 (2 μM) or PAC3 (2 μM) for the indicated time. Phosphorylated (phospho-) TBK1 and IRF3 were detected by immunoblotting assay. **(D)** Primary human hepatocytes were treated with PAC5 (2 μM) or PAC3 (2 μM) in the presence or absence of HBV virus for 12 hours. Phosphorylated (phospho-) TBK1 and IRF3 were detected by immunoblotting assay. Three independent experiments were performed. All data were presented as means ± SD. *p< 0.05, **p<0.01, NS, not significant, calculated by two-way ANOVA with Bonferroni post hoc test (B-D). One representative experiment was shown.


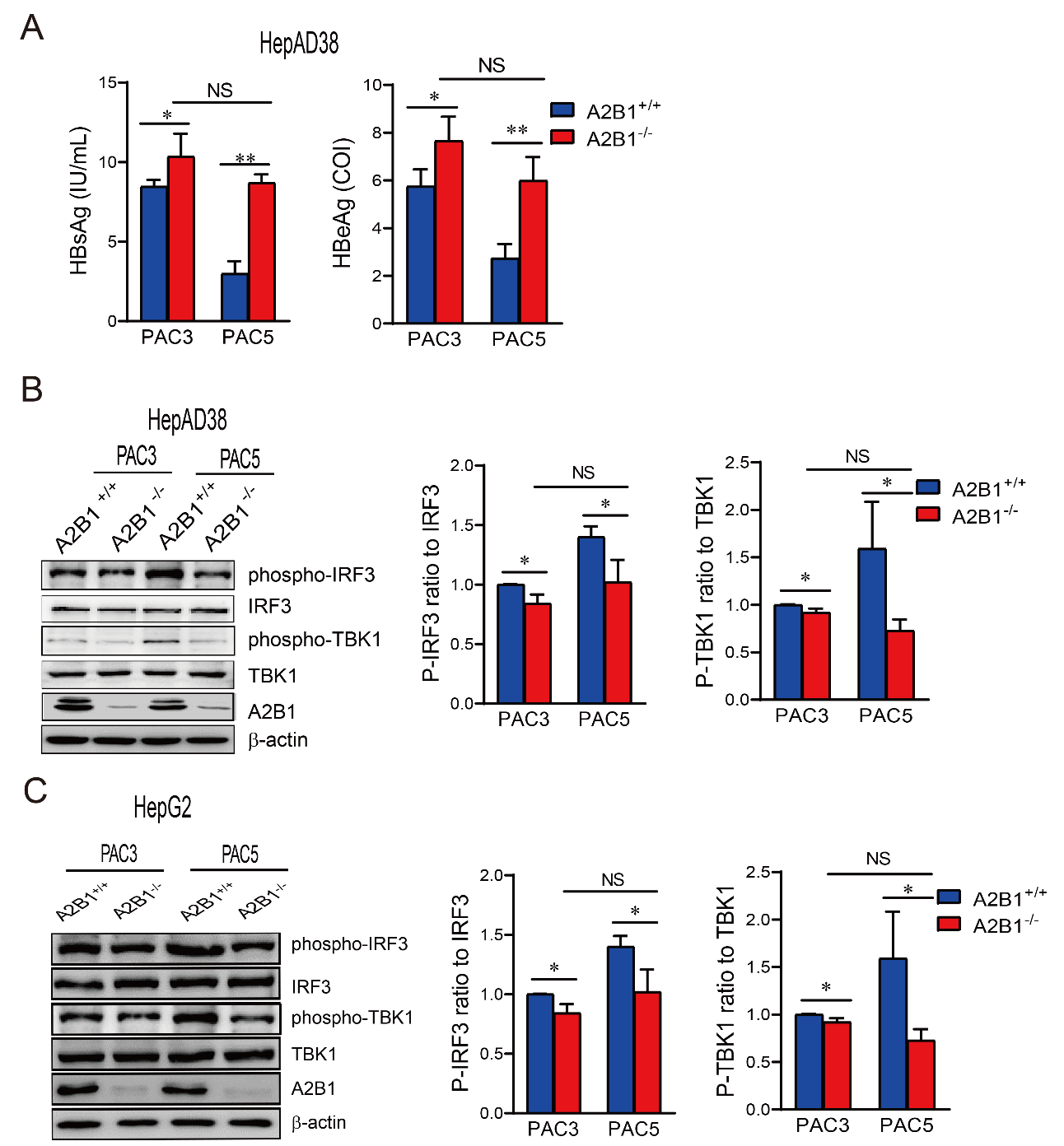


**Supplementary figure 8. TBK1-IRF3 signaling activation attributes to PAC5-bound hnRNPA2B1**

**(A)** hnRNPA2B1^+/+^ and hnRNPA2B1^-/-^ HepAD38 cells were treated with PAC5 (2 μM) or PAC3 (2 μM) for 24 hours. The productions of HBsAg and HBeAg were detected in the cell culture supernatants via CMIA. **(B)** hnRNPA2B1^+/+^ and hnRNPA2B1^-/-^ HepAD38 cells were treated with PAC5 (2 μM) or PAC3 (2 μM) for 9 hours, and the phosphorylations of TBK1 and IRF3 were determined by western blotting analysis. **(C)** hnRNPA2B1^+/+^ and hnRNPA2B1^-/-^ HepG2 cells were treated with PAC5 (2 μM) or PAC3 (2 μM) for 9 hours, and the phosphorylations of TBK1 and IRF3 proteins were determined by western blotting analysis. Three independent experiments were performed. All data were presented as means ± SD. *p< 0.05, **p<0.01, NS, not significant, calculated by two-way ANOVA with Bonferroni post hoc test (A-C). One representative experiment was shown.


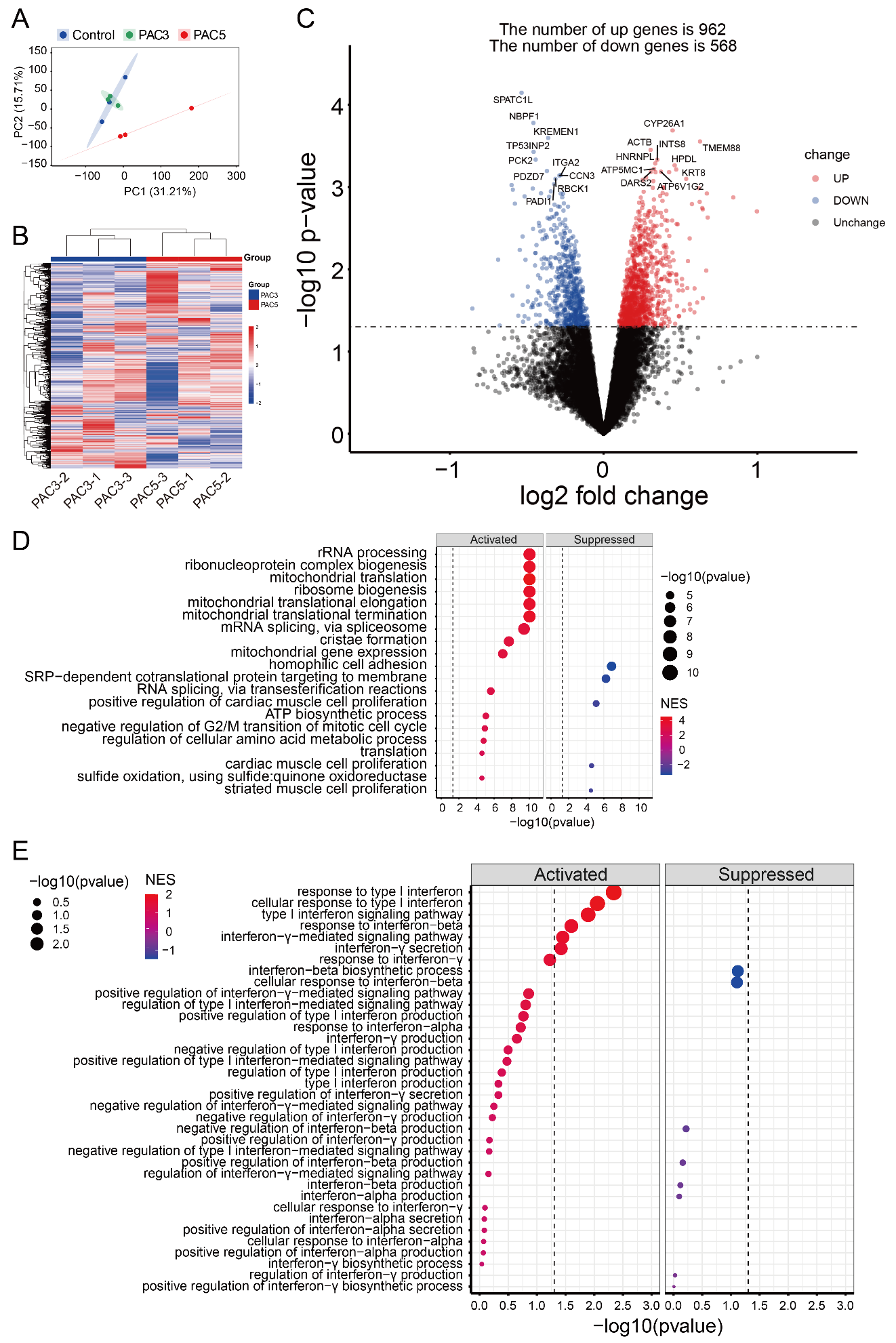


**Supplementary figure 9. Transcriptional characteristics of HepG2.2.15 cells treated with the compound PAC5**

**(A)** Principal component analysis (PCA) showing the distribution of blank control, negative control (NC, PAC3) and PAC5-treated samples based on overall of gene expression. **(B)** Heatmap showing the gene expression of negative control (NC) and PAC5-treated samples. The expression values (TPM) were log2 transformed. A gradient of blue, white, and red indicates low to high expression levels. **(C)** The volcano plot displaying the differential expressed genes in HepG2.2.15 cell treated with the compound PAC5. The dotted line indicates the P value of 0.05, a threshold to identify differential expressed genes (DEG). **(D**-**E)** Bubble chart showing the enrichment result of biological process obtained by Gene set enrichment analysis (GSEA). In **(D)** the top 20 most affected biological processes were shown with activated and suppressed states. In **(E)** all “interferon” related biological processes were shown. The normalized enrichment score (NES) was shown with a gradient of blue and red. The size of bubble corresponds to the significance of biological process. The dotted line indicates the P value of 0.05 used to determine the significance of enrichment.


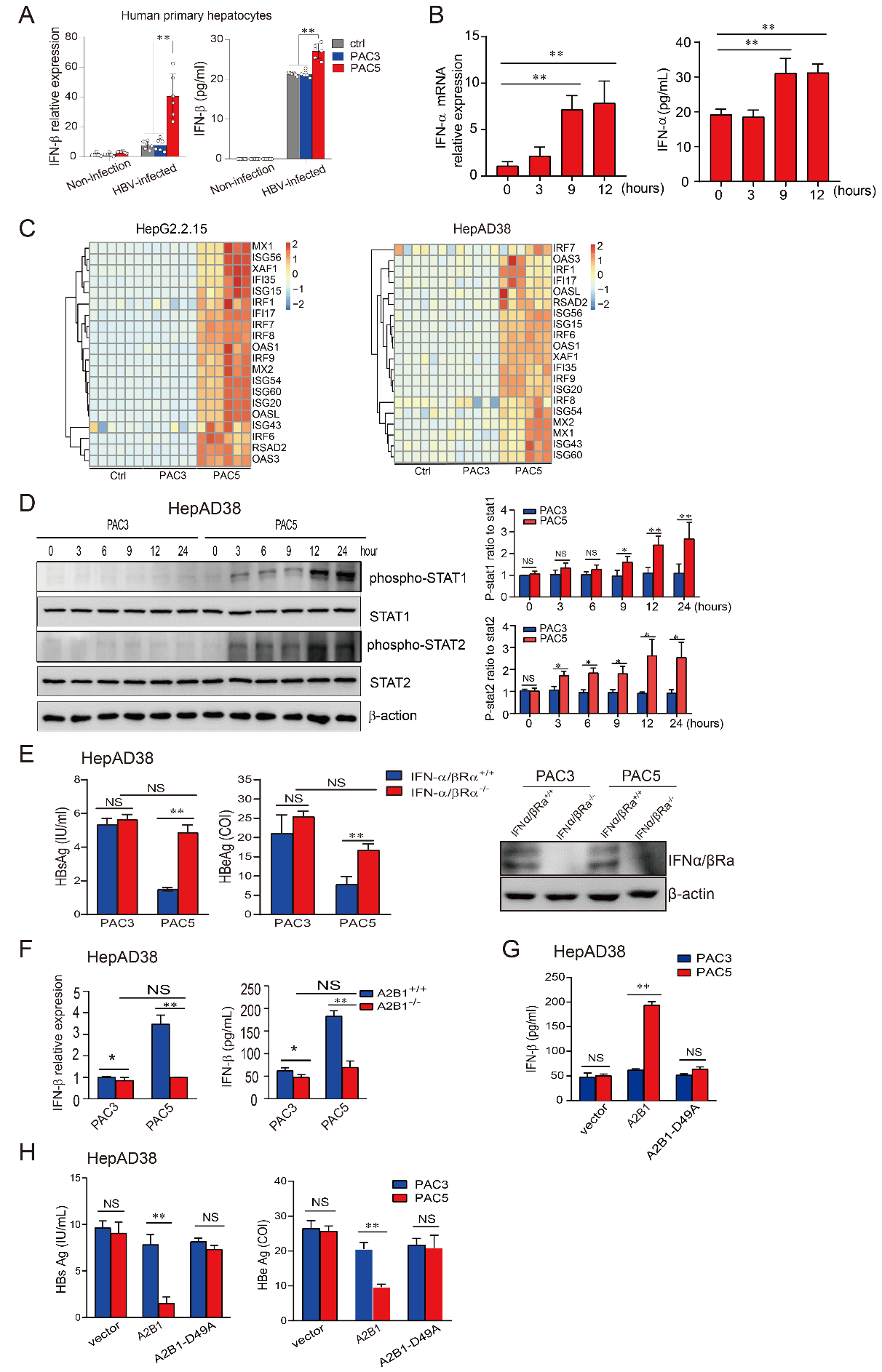


**Supplementary figure 10. Transcriptional characteristics of HepG2.2.15 cells treated with the compound PAC5**

**(A)** Primary human hepatocytes were treated with PAC5 (2 μM) or PAC3 (2 μM) in the presence or absence of HBV virus for 12 hours, and the production of IFN-β was determined at both transcriptional and translational levels. **(B)** HepG2.2.15 cells were treated with PAC5 (2 μM) for indicated time points, and the production of IFN-α was determined at both transcriptional and translational levels. **(C)** HepG2.2.15 and HepAD38 cells were treated with or without PAC5 (2 μM) for 9 hours. mRNA levels of the indicated genes in the HBV-infected cells were detected by quantitative RT-PCR analysis. **(D)** HepAD38 cells were treated with PAC5 (2 μM) or PAC3 (2 μM) for the indicated time. Phosphorylated and total stat1, stat2 were detected by immunoblotting. **(E)** IFN-α/βRα^+/+^ and IFN-α/βRα^-/-^ HepG2.2.15 cells were treatment with PAC3 (2 μM) and PAC5 (2 μM) for 24 hours, and the levels of HBsAg and HBeAg in the cell culture supernatants were analyzed via CMIA. **(F)** hnRNPA2B1^+/+^ and hnRNPA2B1^-/-^ HepAD38 cells were treated with PAC3 (2 μM) or PAC5 (2 μM) for 9 hours, and the mRNA level of IFN-β was detected by quantitative RT-PCR analysis. The culture supernatants were collected at 24 hours after PAC5 stimulation, and IFN-β concentrations were assayed by ELISA. **(G**-**H)** hnRNPA2B1^-/-^ HepAD38 cells were transfected with vector, hnRNPA2B1 or hnRNPA2B1-D49A, then treated with PAC3 (2 μM) or PAC5 (2 μM) for 24 hours. The amount of IFN-β protein in the cell culture supernatants was evaluated via ELISA (G). and the productions of HBsAg and HBeAg were detected by CMIA (H). Three independent experiments were performed. All data were presented as means ± SD. *p< 0.05,**p<0.01, NS, not significant, calculated by one-way ANOVA with Bonferroni post hoc test (B) and two-way ANOVA with Bonferroni post hoc test (A, D, E-H), respectively. One representative experiment showed.

Supplementary Text

**Preparation and characterization of PAC5, PAC3, probes and PAC5 derivatives**

**General information**

All solvents and reagents were purchased from Energy Chemical, Adamas, J&K Scientific and Sigma Aldrich, and used as received. ESI data was recorded on an Agilent 1290 UPLC/6540 Q-TOF mass spectrometer. ^1^H and ^13^C NMR spectra were measured on a Bruker AV 600 MHz spectrometer. Unless otherwise stated, the sample was dissolved in CD_3_OD with TMS as an internal standard. The silica gel used for column chromatography was Fuji NH amino silicone filler (500 mesh). MeOH/DCM (0-10% gradient) was used as the mobile phase. HPLC analysis was performed using a Waters system (5 μM particle size, 4.6 mm × 250 mm) with a PDA detector and a GL InertSustain C18 column. A linear gradient of 5% to 100% water in acetonitrile was used in HPLC analysis at a flow rate of 1 mL/min. The purity of all test compounds was determined by HPLC analysis to be 95% or higher.

**Isolation of phyllanthacidoid A (PA)**

The air-dried stems of *P. acidus* (500 kg) were extracted with methanol solution at room temperature for three times to give the extract 4.21 kg. The methanol extract was resuspended in MeOH (5.0 L) and loaded onto a polyamide gel column (5200 g bed volume, pre-equilibrated with MeOH) to remove pigments and polyphenols. The column was eluted with MeOH (100.5 L), and the eluent evaporated to obtain a crude extract. The crude extract was subjected to passage over a silica gel column (200-300 mesh), eluting with (CHCl_3_-MeOH-H_2_O, 9:1:0-7:3:0.5), to afford five major fractions. Fraction 3 (700 g) was chromatographed on Sephadex LH20 (MeOH 0-100%) to afford five fractions. The first two fractions (400.3 g) were combined and chromatographed over MCI gel CHP-20P (MeOH/H_2_O, 20-100%) to give seven sub-fractions (Fr. A-G). Fr. A was subjected to RP-8 (MeOH 30-80%), and preparative-HPLC with an isocratic flow of CH_3_CN/H_2_O (10 mL/min, CH_3_CN/H_2_O 18:82, COSMOSIL Cholester, 21.1 × 250 mm) to afford phyllanthacidoid A (50.2 g, Rt = 21 min). The NMR data was reported in our previous publication.

**Preparation and characterization of PAC5 derivatives**

**(2S,3R,3aS,4S,4'S,5'R,6S,7aR)-3,3a,4-Trihydroxy-4'-((4-hydroxybenzoyl)oxy)-5'-methyldecahydro-3H-spiro[benzofuran-2,2'-pyran]-6-carboxylic acid (1a)**

Phyllanthuol A (PA, 50 mg, 6.68×10^-5^ mol) was dissolved in K_2_CO_3_ aqueous solution (10%, 2.0 mL) and stirred at 60 ^o^C for about two hours until a complete conversion was detected. The reaction solution was cooled to room temperature and acidified by adding 1 M hydrochloric acid to pH 2. The mixture was combined with brine (2.5 mL) and extracted with EtOAc (4.5 mL × 3). The organic layers were combined and concentrated under reduced pressure. The crude product was purified by silica gel column chromatography to obtain the titled compound as a white solid (27 mg, 92%).

^1^H NMR (600 MHz, CD_3_OD) δ 7.99 (ddd, *J* = 8.8, 2.6, 1.8 Hz, 2H, H-17, H-21), 6.84 (ddd, *J* = 8.8, 2.6, 1.8 Hz, 2H, H-18, H-20), 5.25 (br d, *J* = 2.6 Hz, 1H, H-10), 4.09 (t, *J* = 3.3 Hz, 1H, H-5), 4.04 (t, *J* = 11.4 Hz, 1H, H-12), 3.85 (dd, *J* = 10.8, 4.9 Hz, 1H, H-1), 3.82 (s, 1H, H-7), 3.61 (dd, *J* = 11.1, 4.5 Hz, 1H, H-12), 2.56 (br s, 1H, H-3), 2.20 – 2.16 (m, 1H, H-9), 2.14 –2.06 (m, 2H, H-11, H-9), 2.03 – 1.87 (m, 3H, H-2, H-4), 1.62 (dt, *J* = 13.9, 10.1 Hz, 1H, H-2), 0.90 (d, J = 6.9 Hz, 3H, H-13).

^13^C NMR (150 MHz, CD_3_OD) δ 166.67 (C-22), 162.05 (C-19), 131.57 (C-17, C-21), 121.62 (C-16), 114.63 (C-18, C-20), 101.00 (C-8), 81.01 (C-5), 75.31 (C-6), 74.50 (C-7), 70.84 (C-1), 69.95 (C-10), 61.62 (C-12), 34.72 (C-3), 33.26 (C-9), 32.81 (C-11), 27.86 (C-2), 26.50 (C-4), 11.65 (C-13).

MS (ESI) *m/z*: 477 [M + K]^+^ for C_21_H_26_O_10_.

**(2S,3R,3aS,4S,4'S,5'R,6S,7aR)-3,3a,4,4'-tetrahydroxy-5'-methyldecahydro-3H-spiro[benzofuran-2,2'-pyran]-6-carboxylic acid (1b)**

PA (30 mg, 4.01×10^-5^ mol) was dissolved in NaOH aqueous solution (2.0 M, 1.0 mL) and stirred at 70 ^o^C for about one hour until a complete conversion was detected. The reaction solution was cooled to room temperature and acidified by adding 1 M hydrochloric acid to pH 1-2. The solution of crude product was purified by large pore adsorption resin column chromagraphy (Diaion HP20SS, water to methanol) to obtain the titled compound as a white solid (11 mg, 85%).

^1^H NMR (600 MHz, CD_3_OD) δ 4.07 (t, *J* = 3.6 Hz, 1H, H-5), 3.89 (br d, *J* = 2.7 Hz, 1H, H-10), 3.86 (dd, *J* = 10.5, 5.0 Hz, 1H, H-12), 3.83 (s, 1H, H-7), 3.81 (t, *J* = 11.6 Hz, 1H, H-1), 3.49 (dd, *J* = 11.2, 4.6 Hz, 1H, H-12), 2.86 (ddd, *J* = 15.1, 9.4, 3.8 Hz, 1H, H-3), 2.14 - 2.05 (m, 3H, H-9, H-2), 1.99 - 1.90 (m, 2H, H-4), 1.85 (dddd, *J* = 11.4, 8.9, 5.8, 3.4 Hz, 1H, H-11), 1.73 (ddd, *J* = 14.1, 10.2, 9.3 Hz, 1H, H-2), 0.91 (d, *J* = 7.0 Hz, 3H, H-13).

MS (ESI) *m/z*: 341 [M + Na]^+^ for *C_14_H_22_O_8_.*

1. **Bromobenzyl (2S,3R,3aS,4S,4'S,5’R,6S,7aR)-4'-((4-((4b-bromobenzyl)oxy)benzoyl)oxy)-3,3a,4-trihydroxy-5'-methyldecahydro-3H-spiro[benzofuran-2,2'-pyran]-6-carboxylate (1c)**

Compound **1a** (16 mg, 3.64×10^-5^ mol) was stirred with potassium carbonate (5.0 equiv, 25.4 mg) and 4-bromobenzyl bromide (2.0 eq, 18.2 mg) in dry DMF (1 mL) until a complete conversion was detected. The reacted mixture was diluted with water. The resulted precipitates were collected by centrifugation, washed with water, and dried to give the titled compound as a white solid (23.0 mg, 81%).

^1^H NMR (600 MHz, CDCl_3_) δ 8.04 (d, 2H *J* = 8.9, H-17, H-21), 7.51 (d, 2H, *J* = 8.3, H-4', H-6'), 7.43 (d, 2H, *J* = 8.4, H-4'', H-6''), 7.28 (d, 2H, *J* = 8.4, H-3'', H-7''), 7.12 (d, 2H, *J* = 8.3, H-3', H-7'), 6.96 (d, 2H, *J* = 8.9, H-18, H-20), 5.27 (d, 1H, *J* = 2.2, H-10), 5.05-4.97 (m, 4H, H-1', H-1''), 4.12 (br d, 1H, *J* = 3.7, H-5), 4.06 (br d, 1H, *J* = 11.5, H-12), 3.96 (s, 1H, H-7), 3.93 (dd, 1H, *J* = 9.9, 5.28, H-1), 3.64 (dd, 1H, *J* = 11.08, 4.40, H-12), 2.62 (ddd, 1H, *J* = 16.98, 13.44, 6.84, H-3), 2.18 - 2.06 (m, 4H, H-11, H-9, H-2), 1.95 (ddd, 1H, *J* = 14.58, 10.8, 3.78, H-4), 1.87 (ddd, 1H, *J* = 14.22, 9.30, 8.70, H-4), 1.73 – 1.65 (m, 1H, H-2), 0.88 (d, 3H, *J* = 6.90, H-13).

MS (ESI) *m/z*: 799 [M + Na]^+^ for C_35_H_36_Br_2_O_10_.

**Methyl(2S,3R,3aR,4S,4'S,5'R,6S,7aR)-3,3a,4-trimethoxy-4'-((4-methoxybenzoyl)oxy)-5'- methyldecahydro-3H-spiro[benzofuran-2,2'-pyran]-6-carboxylate (1d)**

Compound **1a** (16 mg, 3.64×10^-5^ mol) was stirred with sodium hydride (60% in mineral oil, 10.0 equiv, 14.6 mg) in dry DMF (1 mL) at room temperature for 2 hours. The mixture was cooled to -40 ^o^C, and methyl iodide (8.0 equiv) was added. The reaction was stirred at room temperature until a complete conversion was detected. The reaction was neutralized by adding 10% citric acid aqueous solution, and extracted with EtOAc (5.0 ml x 3). The combined organic layers were concentrated under reduced pressure. The crude product was purified by thin layer preparative chromatography to give the titled compound as a white solid (7.4 mg, 40%).

^1^H NMR (600 MHz, CD_3_OD) δ 8.03 (d, 2H, *J* = 8.9, H-17, H-21), 6.97 (d, 2H, *J* = 8.9, H-18, H-20), 5.15 (br d, 1H, *J* = 2.1, H-10), 4.18 (br d, 1H, *J* = 2.6, H-5), 4.04 (br d, 1H, *J* = 11.5, H-12), 3.85 (s, 3H, H-5'), 3.60 (s, 3H, H-1'), 3.58 (m, 1H, H-1, overlap), 3.56 (s, 1H, H-7), 3.52 (dd, 1H, *J* = 10.2, 5.1, H-12), 3.49 (s, 3H, H-2'), 3.38 (s, 3H, H-3'), 3.31 (s, 3H, H-4', overlap), 2.26 (m, 2H, H-11, H-3), 2.12 (m, 1H, H-9), 2.04 (m, 1H, H-9), 1.99 (br dd, 1H, *J* = 14.8, 2.9, H-2), 1.77 (ddd, 1H, *J* = 14.0, 6.6, 3.5, H-4), 1.68 (ddd, 1H, *J* = 14.8, 12.2, 2.8, H-4), 1.60 (m, 1H, H-2), 0.90 (d, 3H, *J* = 6.9, H-13).

MS (ESI) *m/z*: 531 [M + Na]^+^ for C_26_H_36_O_10_.

**Methyl (2S,3R,3aR,4S,4'S,5'R,6S,7aR)-3,3a,4,4'-tetramethoxy-5'-methyldecahydro-3H-spi- ro[benzofuran-2,2'-pyran]-6-carboxylate (1e)**

Compound **1b** (10 mg, 3.14 ×10^-5^ mol) was stirred with sodium hydride (60% in mineral oil, 10.0 equiv, 12.6 mg) in dry DMF (1 mL) at room temperature for 2 hours. The mixture was cooled to -40 ^o^C, and methyl iodide (8.0 equiv) was added. The reaction was stirred at room temperature until a complete conversion was detected. The reaction was stirred at room temperature until a complete conversion was detected. The reaction was neutralized by adding 10% citric acid aqueous solution, and extracted with EtOAc (5.0 ml x 3). The combined organic layers were concentrated under reduced pressure. The crude product was purified by thin layer preparative chromatography to give the titled compound as a white solid (**1e**, 6.0 mg, 49%).

^1^H NMR (600 MHz, CD_3_OD) δ 4.17 (br d, 1H, *J* = 2.8, H-5), 3.78 (br d, 1H, *J* = 11.3 Hz, H-12), 3.69 (s, 3H, H-1'), 3.59 (dd, 1H, *J* = 9.7, 3.6, H-12), 3.55 (s, 1H, H-7), 3.49 (s, 3H, H-2'), 3.46 (br d, 1H, *J* = 2.63, H-1), 3.40 (s, 3H, H-3'), 3.39 (s, 3H, H-4'), 3.32 (s, 3H, H-5'), 2.82 (td, 1H, *J* = 12.5, 12.4, 6.3, H-3), 2.26 (dd, 1H, *J* = 14.7, 2.5 Hz, H-11), 2.16 (m, 1H, H-9, overlap), 1.96 (ddd, 1H, *J* = 14.1, 5.4, 3.9, H-9), 1.91 (ddd, 1H, *J* = 14.4, 7.2, 4.5, H-2), 1.79-1.70 (m, 3H, H-4, H-4, H-2), 0.87 (d, 3H, *J* = 7.0, H-13).

MS (ESI) *m/z*: 388 [M + Na]^+^ for C_19_H_32_O_8_.

**General procedure A: Preparation of ester derivatives of compound 1a**

Compound **1a** (6 mg, 1.37×10^-5^ mol) was stirred with EDCI (10.0 equiv), DMAP (2.0 equiv) and the corresponding alcohol (10 equiv) in dry DMF (1.0 mL) at room temperature until a complete conversion was detected. The mixture was partitioned between EtOAc and brine, and the organic layer was washed with brine and concentrated. The crude product was purified by silica gel column chromatography to obtain the corresponding titled compound as a white solid.

Compounds prepared with this procedure: **2a**, 22%; **2b**, 43%; **2c**, 49%; **2d**, 40%.

**Methyl (2S,3R,3aS,4S,4'S,5'R,6S,7aR)-3,3a,4-trihydroxy-4'-((4-hydroxybenzoyl)oxy)-5'-me- thyldecahydro-3H-spiro[benzofuran-2,2'-pyran]-6-carboxylate (2a)**

^1^H NMR (600 MHz, CD_3_OD) δ 7.97 (br dd, *J* = 6.8, 1.9 Hz, 2H, H-17, H-21 ) ,6.83 (ddd, *J* = 6.8, 1.9 Hz, 2H, H-18, H-20), 5.22 (br d, *J* = 2.5 Hz, 1H, H-10), 4.06 – 3.99 (m, 2H, H-5, H-12), 3.82 – 3.75 (m, 2H, H-1, H-7), 3.61 (m, 4H, H-12, H-1'), 2.54 (br q, *J* = 5.6, 3.5 Hz, 1H, H-3), 2.17 – 2.07 (m, 3H, H-11, H-9), 1.98 – 1.90 (m, 2H, H-2, H-4), 1.85 – 1.78 (m, 1H, H-4), 1.61 – 1.52 (m, 1H, H-2), 0.87 (d, *J* = 6.91 Hz, 3H, H-13).

MS (ESI) *m/z*: 375 [M + Na]^+^ for C_22_H_28_O_10_.

**Propyl (2S,3R,3aS,4S,4'S,5'R,6S,7aR)-3,3a,4-trihydroxy-4'-((4-hydroxybenzoyl)oxy)-5'-me- thyldecahydro-3H-spiro[benzofuran-2,2'-pyran]-6-carboxylate (2b)**

^1^H NMR (600 MHz, CD_3_OD) δ 7.97 (ddd, *J* = 8.82, 2.64, 1.98 Hz, 2H, H-17, H-21), 6.83 (ddd, *J* = 8.82, 2.67, 2.00 Hz, 2H, H-18, H-20), 5.22 (br d, *J* = 2.6 Hz, 1H, H-10), 4.08 – 4.03 (m, 2H, H-5, H-12), 4.03 – 3.96 (m, 2H, H-1'), 3.85 – 3.78 (m, 2H, H-1, H-7), 3.62 (dd, *J* = 11.0, 4.5 Hz, 1H, H-12), 2.54 (tt, *J* = 9.3, 5.7 Hz, 1H, H-3), 2.17 – 2.07 (m, 3H, H-11, H-9), 2.00 – 1.93 (m, 2H, H-2, H-4), 1.86 (ddd, *J* = 14.5, 11.5, 3.1 Hz, 1H, H-2), 1.67 – 1.52 (m, 2H, H-2'), 0.92 (t, *J* = 7.43 Hz, 3H, H-3'), 0.91 (d, *J* = 6.91 Hz, 3H, H-13).

MS (ESI) *m/z*: 503 [M + Na]^+^ for C_24_H_32_O_10_.

**Butyl (2S,3R,3aS,4S,4'S,5'R,6S,7aR)-3,3a,4-trihydroxy-4'-((4-hydroxybenzoyl)oxy)-5'-met- hyldecahydro-3H-spiro[benzofuran-2,2'-pyran]-6-carboxylate (2c)**

^1^H NMR (600 MHz, CD_3_OD) δ 7.97 (br d, *J* = 8.7 Hz, 2H, H-17, H-21), 6.83 (br d, *J* = 8.7 Hz, 2H, H-18, H-20), 5.22 (br d, *J* = 2.5 Hz, 1H, H-10), 4.11 – 3.97 (m, 4H, H-5, H-12, H-1'), 3.81 (dd, *J* = 11.0, 4.5 Hz, 1H, H-1), 3.81 (s, 7-H), 3.62 (dd, *J* = 11.1, 4.6 Hz, 1H, H-12), 2.54 (qd, *J* = 11.3, 5.7 Hz, 1H, H-3), 2.17 – 2.07 (m, 3H, H-11, H-9), 2.01 – 1.91 (m, 2H, H-2, H-4), 1.86 (ddd, *J* = 14.5, 11.5, 3.1 Hz, 1H ,H-4), 1.60 – 1.55 (m, 3H, H-2, H-2'), 1.39 – 1.32 (m, 2H, H-3'), 0.95 (t, *J* = 7.41 Hz, 3H, H-4'), 0.91 (t, *J* = 6.93 Hz, 3H, H-13).

MS (ESI) *m/z*: 517 [M + Na]^+^ for C_25_H_34_O_10_.

**Pentyl (2S,3R,3aS,4S,4'S,5'R,6S,7aR)-3,3a,4-trihydroxy-4'-((4-hydroxybenzoyl)oxy)-5'-met- hyldecahydro-3H-spiro[benzofuran-2,2'-pyran]-6-carboxylate (2d)**

^1^H NMR (600 MHz, CD_3_OD) δ 7.98 (ddd, *J* = 8.73, 2.63, 1.88 Hz, 2H, H-17, H-21), 6.83 (ddd, *J* = 8.72, 2.63, 1.85 Hz, 2H, H-18, H-20), 5.23 (br d, *J* = 2.6 Hz, 1H, H-10), 4.09 – 3.96 (m, 4H, H-5, H-12, H-1'), 3.82 (dd, *J* = 10.6 , 5.0 Hz, 1H, H-1), 3.82 (s, 1H, H-7), 3.62 (dd, *J* = 11.1, 4.5 Hz, 1H, H-12), 2.54 (qd, *J* = 11.3, 5.7 Hz, 1H, H-3), 2.18 – 2.08 (m, 3H, H-11, H-9), 2.00 – 1.94 (m, 2H, H-2, H-4), 1.86 (ddd, *J* = 14.5, 11.5, 3.1 Hz, 1H, H-4), 1.64 – 1.55 (m, 3H, H-2, H-2'), 1.40 – 1.27 (m, 4H, H-3', H-4'), 0.93 (t, *J* = 7.2 Hz, 3H, H-5'), 0.91 (d, *J* = 6.9 Hz, 3H, H-13).

MS (ESI) *m/z*: 531 [M + Na]^+^ for C_26_H_36_O_10_.

**3General procedure B: Preparation of ester derivatives of compound 1a**

Compound **1a** (6 mg, 1.37×10^-5^ mol) was stirred with potassium carbonate (5.0 equiv) and the corresponding alkyl bromide (2-5 equiv) in dry DMF (1.0 mL) at room temperature until a complete conversion was detected. The mixture was partitioned between EtOAc and brine, and the organic layer was washed with brine and concentrated. The crude product was purified by silica gel column chromatography to obtain the corresponding titled compound as a white solid.

Compounds prepared with this procedure: **2e**, 49%; **2f**, 56%; **2g**, 83%.

**3-Fluoropropyl (2S,3R,3aS,4S,4'S,5'R,6S,7aR)-4'-((4-(3-fluoropropoxy)benzoyl)oxy)-3,3a,4- trihydroxy-5'-methyldecahydro-3H-spiro[benzofuran-2,2'-pyran]-6-carboxylate (2e)**

^1^H NMR (600 MHz, CD_3_OD) δ 8.08 (br d, *J* = 8.8 Hz, 2H, H-17, H-21), 7.03 (br d, *J* = 8.8 Hz, 2H, H-18, H-20), 5.24 (br d, *J* = 2.4 Hz, 1H, H-10), 4.40 (t, *J* = 5.8 Hz, 4H, H-3', H-3''), 4.19 (t, *J* = 6.2 Hz, 2H, H-1''), 4.17 – 4.10 (m, 2H, H-1'), 4.09 – 4.03 (m, 2H, H-5, H-12), 3.84 – 3.76 (m, 2H, H-1, H-7), 3.63 (dd, *J* = 11.1, 4.6 Hz, 1H, H-12), 2.53 (qd, *J* = 11.4, 5.7 Hz, 1H, H-3), 2.25 – 2.06 (m, 5H, H-11, H-9, H-2''), 2.01 – 1.90 (m, 4H, H-2, H-4, H-2'), 1.86 (ddd, *J* = 14.5, 11.6, 3.1 Hz, 1H, H-4), 1.58 (dt, *J* = 14.1, 9.7 Hz, 1H, H-2), 0.92 (d, *J* = 6.9 Hz, 3H, H-13).

MS (ESI) *m/z*: 581 [M + Na]^+^ for C_27_H_36_F_2_O_10_.

**4-Fluorobutyl (2S,3R,3aS,4S,4'S,5'R,6S,7aR)-4'-((4-(4-fluorobutoxy)benzoyl)oxy)-3,3a,4-tr- ihydroxy-5'-methyldecahydro-3H-spiro[benzofuran-2,2'-pyran]-6-carboxylate (2f)**

^1^H NMR (600 MHz, CD_3_OD) δ 8.08 (br d, *J* = 9.0 Hz, 2.7 Hz, 2.0 Hz, 2H, H-17, H-21), 7.01 (br d, *J* = 9.0 Hz, 2.7 Hz, 2.0 Hz, 2H, H-18, H-20), 5.24 (d, *J* = 2.6 Hz, 1H, H-10), 4.57 – 4.40 (4H, H-4', H-4''), 4.15 – 4.03 (m, 6H, H-5, H-12, H-1', H-1''), 3.84 – 3.76 (m, 2H, H-1, H-7), 3.66 – 3.59 (m, 1H, H-12), 2.58 – 2.50 (m, 1H, H-3), 2.22 – 2.09 (m, 3H, H-11, H-9, overlap with acetone), 2.01 – 1.83 (m, 7H, H-2, H-4, H-4, H-2', H-2''), 1.77 – 1.64 (m, 4H, H-3', H-3''), 1.59 (ddd, *J* = 14.1, 10.3, 9.2 Hz, 1H, H-2), 0.91 (d, *J* = 6.9 Hz, 2H, H-13).

MS (ESI) *m/z*: 609 [M + Na]^+^ for C_29_H_40_F_2_O_10_.

**5-Fluoropentyl (2S,3R,3aS,4S,4'-S,5'R,6S,7aR)-4'-((4-((5-fluoropentyl)oxy)benzoyl)oxy)-3, 3a,4-trihydroxy-5'-methyldecahydro-3H-spiro[benzofuran-2,2'-pyran]-6-carboxylate (2g)**

^1^H NMR (600 MHz, CD_3_OD) δ 8.07 (br d, *J* = 8.8 Hz, 2H, H-17, H-21), 7.00 (br d, *J* = 8.8 Hz, 2H, H-18, H-20), 5.24 (br d, *J* = 2.2 Hz, 1H, H-10), 4.39 (t, *J* = 6.0 Hz, 4H, H-5', H-5''), 4.14 – 3.98 (m, 6H, H-5, H-12, H-1', H-1'), 3.86 – 3.77 (m, 2H, H-1, H-7), 3.62 (dd, *J* = 11.1, 4.6 Hz, 1H, H-12), 2.54 (qd, *J* = 11.4, 5.7 Hz, 1H, H-3), 2.19 – 2.07 (m, 3H, H-11, H-9), 2.02 – 1.93 (m, 2H, H-2''), 1.91 – 1.39 (m, 14H, H-2, H-4, H-2', H-3', H-4', H-3'', H-4''), 0.91 (d, *J* = 6.9 Hz, 3H, H-13).

MS (ESI) *m/z*: 637 [M + Na]^+^ for C_31_H_44_F_2_O_10_.

**General procedure C: Preparation of amide derivatives of compound 1a**

Compound **1a** (6 mg, 1.37×10^-5^ mol) was stirred with EDCI (10.0 equiv), DMAP (2.0 equiv), HOSu (5.0 equiv) and the corresponding amine (5.0 equiv) in dry DMF (1.0 mL) at room temperature until a complete conversion was detected. The mixture was partitioned between EtOAc and diluted hydrochloric acid, and the organic layer was washed with brine and concentrated. The crude product was purified by silica gel column chromatography to obtain the corresponding titled compound as a white solid.

Compounds prepared with this procedure: **3a**, 22%; **3b**, 43%; **3c**, 49%; **3d**, 7.5%; **3e**, 49%; **3f**, 69%; **3g**, 59%; **3h**, 66%; **3i**, 74%; **3j**, 47%; **4a**, 53%; **4b**, 54%; **4c**, 56%; **4d**, 54%; **4e**, 66%; **4f**, 72%; **4g**, 44%; **4h**, 27%; **4i**, 74%; **4j**, 76%; **4k**, 82%; **4l**, 59%; **4m**, 48%; **4n**, 30%; **4o**, 27%; **4p**, 22%.

**2S,3R,3aS,4S,4'S,5'R,6S,7aR)-6-(ethylcarbamoyl)-3,3a,4-trihydroxy-5'-methyldecahydro-3H-spiro[benzofuran-2,2'-pyran]-4'-yl 4-hydroxybenzoate (3a)**

^1^H NMR (600 MHz, CD_3_OD) δ 7.98 (br d, *J* = 8.7 Hz, 2H, H-17, H-21), 6.84 (br d, *J* = 8.7 Hz, 2H, H-18, H-20), 5.24 (br d, *J* = 2.2 Hz, 1H, H-10), 4.12 (t, *J* = 3.1 Hz, 1H, H-5), 4.05 (t, *J* = 11.4 Hz, 1H, H-12), 3.90 (dd, *J* = 10.5, 5.5 Hz, 1H, H-1), 3.76 (s, 1H, H-7), 3.61 (dd, *J* = 11.1, 4.5 Hz, 1H, H-12), 3.16 – 3.08 (m, 2H, H-1'), 2.41 (qd, *J* = 11.2, 5.6 Hz, 1H, H-3), 2.17 (dd, *J* = 14.8, 3.1 Hz, 1H, H-11), 2.14 – 2.07 (m, 2H, H-9), 1.99 (ddd, *J* = 14.6, 11.8, 3.1 Hz, 1H, H-2), 1.80 (dt, *J* = 13.9, 5.6 Hz, 1H, H-4), 1.71 (ddd, *J* = 14.4, 5.2, 3.7 Hz, 1H, H-4), 1.57 (dt, *J* = 13.9, 9.9 Hz, 1H, H-2), 1.05 (t, *J* = 7.3 Hz, 3H, H-2'), 0.90 (d, *J* = 6.9 Hz, 3H, H-13).

MS (ESI) *m/z*: 488 [M + Na]^+^ for C_23_H_31_NO_9_.

**(2S,3R,3aS,4S,4'S,5'R,6S,7aR)-3,3a,4-trihydroxy-5'-methyl-6-(propylcarbamoyl)decahydro-3H-spiro[benzofuran-2,2'-pyran]-4'-yl 4-hydroxybenzoate (3b)**

^1^H NMR (600 MHz, CD_3_OD) δ 7.97 (br d, *J* = 8.7 Hz, 2H, H-17, H-21), 6.83 (br d, *J* = 8.7 Hz, 2H, H-18, H-20), 5.24 (br d, *J* = 2.3 Hz, 1H, H-10), 4.12 (t, *J* = 3.1 Hz, 1H, H-5), 4.05 (t, *J* = 11.4 Hz, 1H, H-12), 3.91 (dd, *J* = 10.5, 5.6 Hz, 1H, H-1), 3.76 (s, 1H, H-7), 3.62 (dd, *J* = 11.1, 4.5 Hz, 1H, H-12), 3.10 – 3.00 (m, 2H, H-1'), 2.48 – 2.40 (m, 1H, H-3), 2.17 (dd, *J* = 14.8, 3.1 Hz, 1H, H-11), 2.14 – 2.06 (m, 2H, H-9), 2.00 (ddd, *J* = 14.6, 11.8, 3.1 Hz, 1H, H-2), 1.81 (dt, *J* = 13.9, 5.7 Hz, 1H, H-4), 1.74 – 1.67 (m, 1H, H-4), 1.58 (dt, *J* = 14.0, 9.9 Hz, 1H, H-2), 1.48 – 1.40 (m, 2H, H-2'), 0.90 (d, *J* = 6.9 Hz, 1H, H-13), 0.87 (t, *J* = 7.4 Hz, 1H, H-3').

MS (ESI) *m/z*: 502 [M + Na]^+^ for C_24_H_33_NO_9_.

**(2S,3R,3aS,4S,4'S,5'R,6S,7aR)-6-(butylcarbamoyl)-3,3a,4-trihydroxy-5'-methyldecahydro-3H-spiro[benzofuran-2,2'-pyran]-4'-yl 4-hydroxybenzoate (3c)**

^1^H NMR (600 MHz, CD_3_OD) δ 7.97 (ddd, *J* = 8.82, 2.64, 2.04 Hz, 2H, H-17, H-21), 6.84 (ddd, *J* = 8.82, 2.70, 1.98 Hz, 2H, H-18, H-20) ,5.24 (br d, *J* = 2.5 Hz, 1H, H-10), 4.12 (t, *J* = 3.2 Hz, 1H, H-5), 4.05 (t, *J* = 11.4 Hz, 1H, H-12), 3.91 (dd, *J* = 10.5, 5.6 Hz, 1H, H-1), 3.76 (s, 1H, H-7), 3.61 (dd, *J* = 11.0, 4.4 Hz, 1H, H-12), 3.09 (br ddd, *J* = 13.3, 7.1, Hz, 2H, H-1'), 2.44 (ddd, *J* = 15.0, 11.3, 5.6 Hz, 1H, H-3), 2.21 – 2.04 (m, 3H, H-11, H-9), 2.00 (ddd, *J* = 14.7, 9.4, 3.2 Hz, 1H, H-2), 1.81 (dt, *J* = 14.0, 5.7 Hz, 1H, H-4), 1.70 (ddd, *J* = 14.4, 5.3, 3.5 Hz, 1H, H-4), 1.58 (dt, *J* = 14.0, 9.8 Hz, 1H, H-2), 1.43 – 1.36 (m, 2H, H-2'), 1.33 – 1.25 (m, 2H, H-3'), 0.92 (t, *J* = 7.4 Hz, 3H, H-4'), 0.90 (d, *J* = 6.9 Hz, 3H, H-13).

MS (ESI) *m/z*: 516 [M + Na]^+^ for C_25_H_35_NO_9_.

**(2S,3R,3aS,4S,4'-S,5'R,6S,7a-R)-3,3a,4-trihydroxy-5'-methyl-6-(pentylcarbamoyl)decahydro-3H-spiro[benzofuran-2,2'-pyran]-4'-yl 4-hydroxybenzoate (3d)**

^1^H NMR (600 MHz, CD_3_OD) δ 7.97 (ddd, *J* = 8.8, 2.6, 2.0 Hz, 2H, H-17, H-21), 6.84 (ddd, *J* = 8.8, 2.7, 2.0 Hz, 2H, H-18, H-20), 5.24 (br d, *J* = 2.5 Hz, 1H, H-10), 4.12 (t, *J* = 3.2 Hz, 1H, H-5), 4.05 (t, *J* = 11.4 Hz, 1H, H-12), 3.91 (dd, *J* = 10.5, 5.6 Hz, 1H, H-1), 3.76 (s, 1H, H-7), 3.61 (dd, *J* = 11.0, 4.4 Hz, 1H, H-12), 3.09 (br ddd, *J* = 13.3, 7.1, Hz, 2H, H-1'), 2.44 (ddd, *J* = 15.0, 11.3, 5.6 Hz, 1H, H-3), 2.21 – 2.04 (m, 3H, H-11, H-9), 2.00 (ddd, *J* = 14.7, 9.4, 3.2 Hz, 1H, H-2), 1.81 (dt, *J* = 14.0, 5.7 Hz, 1H, H-4), 1.70 (ddd, *J* = 14.4, 5.3, 3.5 Hz, 1H, H-4), 1.58 (dt, *J* = 14.0, 9.8 Hz, 1H, H-2), 1.43 – 1.36 (m, 2H, H-2'), 1.33 – 1.25 (m, 2H, H-3'), 0.92 (t, *J* = 7.4 Hz, 3H, H-4'), 0.90 (d, *J* = 6.9 Hz, 3H, H-13).

^13^C NMR (150 MHz, CD_3_OD) δ 177.01 (C-15), 166.71 (C-22), 162.14 (C-19), 131.67(C-17, C-21), 121.62 (C-16), 114.61 (C-18, C-20), 100.96 (C-8), 81.29 (C-5), 75.12 (C-6), 74.67 (C-7), 70.84 (C-1), 70.08 (C-10), 61.61 (C-12), 38.88 (C-1'), 34.51 (C-2'), 34.16 (C-3), 32.83 (C-9), 29.12 (C-11), 28.72 (C-3'), 28.61 (C-4'), 26.64 (C-2), 21.96(C-5'), 12.96 (C-5'), 11.67 (C-13).

MS (ESI) *m/z*: 530 [M+Na]^+^ for C_26_H_37_NO_9_.

**(2S,3R,3aS,4S,4'S,5'R,6S,7aR)-6-(hexylcarbamoyl)-3,3a,4-trihydroxy-5'-methyldecahydro-3H-spiro[benzofuran-2,2'-pyran]-4'-yl 4-hydroxybenzoate (3e)**

^1^H NMR (600 MHz, CD_3_OD) δ 7.94 (br d, *J* = 8.7 Hz, 2H, H-17, H-21), 6.81 (br d, *J* = 8.7 Hz, 2H, H-18, H-20), 5.21 (br d, *J* = 1.9 Hz, 1H, H-10), 4.09 (t, *J* =2.9 Hz, 1H, H-5), 4.02 (t, *J* = 11.4 Hz, 1H, H-12), 3.88 (dd, *J* = 10.5, 5.6 Hz, 1H, H-1), 3.73 (s, 1H, H-7), 3.59 (dd, *J* = 11.2, 4.5 Hz, 1H, H-12), 3.05 (td, *J* = 7.1, 2.6 Hz, 2H, H-1'), 2.37 – 2.29 (m, 1H, H-3), 2.15 (dd, *J* = 14.8, 3.0 Hz, 1H, H-9), 2.11 – 2.02 (m, 2H, H-11, H-9), 2.00 – 1.93 (m, 1H, H-2), 1.78 (dt, *J* = 13.8, 5.7 Hz, 1H, H-4), 1.71 –1.64 (m, 1H, H-4), 1.55 (dt, *J* = 13.9, 10.0 Hz, 1H, H-2), 1.42 – 1.36 (m, 2H, H-2'), 1.35 – 1.21 (m, 6H, H-3', H-4', H-5'), 0.92 – 0.84 (m, 6H, H-6', H-13).

^13^C NMR (150 MHz, CD_3_OD) δ 178.41 (C-15), 168.14 (C-22), 163.53 (C-19), 133.07 (C-17, C-21), 123.03 (C-16), 116.02 (C-18, C-20), 102.37 (C-8), 82.70 (C-5), 76.54 (C-6), 76.07 (C-7), 72.23 (C-1), 71.50 (C-10), 63.01 (C-12), 40.31 (C-1'), 36.92 (C-2'), 35.91 (C-3), 35.58 (C-9), 34.23 (C-11), 32.59 (C-3'), 30.51 (C-4'), 30.28 (C-2), 27.58 (C-5'), 23.62 (C-4), 14.38 (C-6'), 13.05 (C-13).

MS (ESI) *m/z*: 544 [M+Na]^+^ for C_27_H_39_NO_9_.

**(2S,3R,3aS,4S,4'S,5'R,6S,7aR)-6-(heptylcarbamoyl)-3,3a,4-trihydroxy-5'-methyldecahydro-3H-spiro[benzofuran-2,2'-pyran]-4'-yl 4-hydroxybenzoate (3f)**

^1^H NMR (600 MHz, CD_3_OD) δ 7.93 (br d, *J* = 8.8 Hz, 2H, H-17, H-21), 6.79 (br d, *J* = 8.8 Hz, 2H, H-18, H-20), 5.21 (br d, *J* = 2.2 Hz, 1H, H-10), 4.09 (t, *J* =3.1 Hz, 1H, H-5), 4.02 (t, *J* = 11.4 Hz, 1H, H-12), 3.88 (dd, *J* = 10.5, 5.6 Hz, 1H, H-1),3.73 (s, 1H, H-7), 3.58 (dd, *J* = 11.0, 4.5 Hz, 1H, H-12), 3.05 (t, *J* = 7.1 Hz, 1H, H-1'), 2.48 – 2.37 (m, 1H, H-3), 2.19 – 2.02 (m, 3H, H-9, H-11), 2.00 – 1.93 (m, 1H, H-2), 1.78 (dt, *J* = 13.9, 5.6 Hz, 1H, H-4), 1.68 (ddd, *J* = 14.4, 5.2, 3.6 Hz, 1H, H-4), 1.61 – 1.50 (m, 1H, H-2), 1.44 – 1.19 (m, 10H, H-2', H-3', H-4', H-5', H-6'), 0.93 – 0.82 (m, 6H, H-13, H-7').

^13^C NMR (150 MHz, CD_3_OD) δ 177.01 (C-15), 166.71 (C-22), 162.13 (C-19), 131.67 (C-17, C-21), 121.63 (C-16), 114.60 (C-18, C-20), 100.96 (C-8), 81.29 (C-5), 75.11 (C-6), 74.68 (C-7), 70.84 (C-1), 70.07 (C-10), 61.61 (C-12), 38.90 (C-1'), 34.52 (C-3), 34.17 (C-2'), 32.84 (C-9), 31.53 (C-11), 29.13 (C-3'), 28.93 (C-4'), 28.64 (C-5'), 26.65 (C-6'), 26.48 (C-2), 22.28 (C-4), 13.03 (C-7'), 11.65 (C-13).

MS (ESI) *m/z*: 558 [M+Na]^+^ for C_28_H_41_NO_9_.

**(2S,3R,3aS,4S,4'S,5'R,6S,7aR)-3,3a,4-trihydroxy-5'-methyl-6-(octylcarbamoyl)decahydro-3H-spiro[benzofuran-2,2'-pyran]-4'-yl 4-hydroxybenzoate (3g)**

^1^H NMR (600 MHz, CD_3_OD) δ 7.94 (br d, *J* = 8.7 Hz, 2H, H-17, H-21), 6.80 (br d, *J* = 8.7 Hz, 2H, H-18, H-20), 5.21 (br s, 1H, H-10), 4.09 (br s, 1H, H-5), 4.02 (t, *J* = 11.4 Hz, 1H, H-12), 3.88 (dd, *J* = 10.4, 5.6 Hz, 1H, H-1), 3.73 (s, 1H, H-7), 3.59 (dd, *J* = 11.1, 4.2 Hz, 1H, H-12), 3.05 (t, *J* = 7.0 Hz, 1H, H-1'), 2.41 (dq, *J* = 16.2, 5.5 Hz, 1H, H-3), 2.15 (dd, *J* = 14.7, 2.9 Hz, 1H, H-9), 2.07 (dd, *J* = 14.8, 2.4 Hz, 2H, H-11, H-9), 2.00 – 1.92 (m, 1H, H-2), 1.78 (dt, *J* = 13.7, 5.6 Hz, 1H, H-4), 1.72 – 1.64 (m, 1H, H-4), 1.55 (dt, *J* = 13.8, 10.0 Hz, 1H, H-2), 1.40 (dd, *J* = 14.0, 7.0 Hz, 2H, H-2'), 1.35 – 1.20 (m, 10H, H-3', H-4', H-5', H-6', H-7'), 0.95 – 0.83 (m, 6H, H-13, H-8').

^13^C NMR (150 MHz, CD_3_OD) δ 177.01 (C-15), 166.71 (C-22), 162.13 (C-19), 131.67 (C-17, C-21), 121.64 (C-16), 114.60 (C-18, C-20), 100.96 (C-8), 81.28 (C-5), 75.11 (C-6), 74.68 (C-7), 70.84 (C-1), 70.08 (C-10), 61.61 (C-12), 38.90 (C-1'), 34.52 (C-2'), 34.17 (C-3), 32.84 (C-9), 31.60 (C-3'), 29.13 (C-11), 28.96 (C-4'), 28.92 (C-5', C-6'), 26.66 (C-2), 26.51 (C-7'), 22.32 (C-4), 13.05 (C-8'), 11.65 (C-13).

MS (ESI) *m/z*: 572 [M + Na]^+^ for C_29_H_43_NO_9_.

**(2S,3R,3aS,4S,4'S,5'R,6S,7aR)-6-((3-ethoxypropyl)carbamoyl)-3,3a,4-trihydroxy-5'-methyldecahydro-3H-spiro[benzofuran-2,2'-pyran]-4'-yl 4-hydroxybenzoate (3h)**

^1^H NMR (600 MHz, CD_3_OD) δ 7.95 (br d, *J* = 8.7 Hz, 2H, H-17, H-21), 6.81 (br d, *J* = 8.7 Hz, 2H, H-18, H-20), 5.21 (br s, 1H, H-10), 4.10 (br s, 1H, H-5), 4.02 (t, *J* = 11.4 Hz, 1H, H-12), 3.88 (dd, *J* = 10.4, 5.6 Hz, 1H, H-1), 3.74 (s, 1H, H-7), 3.59 (dd, *J* = 10.9, 4.2 Hz, 1H, H-12), 3.43 (q, *J* = 7.0 Hz, 2H, H-4'), 3.38 (t, *J* = 6.2 Hz, 1H, H-3'), 3.15 (ddq, *J* = 20.3, 13.5, 6.9 Hz, 2H, H-1'), 2.41 (tt, *J* = 11.1, 5.5 Hz, 1H, H-3), 2.14 (dt, *J* = 10.5, 5.3 Hz, 1H, H-11), 2.11 – 2.03 (m, 2H, H-11, H-9), 2.00 – 1.93 (m, 1H, H-2), 1.79 (dt, *J* = 13.7, 5.6 Hz, 1H, H-4), 1.72 – 1.62 (m, 3H, H-4, H-2'), 1.56 (dt, *J* = 13.8, 10.0 Hz, 1H, H-2), 1.14 (t, *J* = 7.0 Hz, 3H, H-5'), 0.87 (d, *J* = 6.9 Hz, 3H, H-13).

MS (ESI) *m/z*: 546 [M + Na]^+^ for C_26_H_37_NO_10_.

**(2S,3R,3aS,4S,4'S,5'R,6S,7aR)-3,3a,4-trihydroxy-5'-methyl-6-(phenethylcarbamoyl)decahydro-3H-spiro[benzofuran-2,2'-pyran]-4'-yl 4-hydroxybenzoate (3i)**

^1^H NMR (600 MHz, CD_3_OD) δ 7.94 (br d, *J* = 8.7 Hz, 2H, H-17, H-21),7.22 (t, *J* = 7.4 Hz, 2H, H-3', H-5'), 7.18 – 7.12 (m, 3H, H-2', H-4', H-6'), 6.82 (br d, *J*= 8.7 Hz, 2H, H-18, H-20), 5.20 (br s, 1H, H-10), 4.08 (br s, 1H, H-5), 4.01 (t, *J* =11.4 Hz, 1H, H-12), 3.86 (dd, *J* = 10.5, 5.5 Hz, 1H, H-1), 3.72 (s, 1H, H-7), 3.58 (dd,*J* = 11.0, 4.2 Hz, 1H, H-12), 3.36 – 3.24 (m, 4H, H-8', overlap), 2.69 (t, *J* = 7.3 Hz, 2H, H-7'), 2.37 (dq, *J* = 16.2, 5.4 Hz, 1H, H-3), 2.14 (dd, *J* = 14.7, 2.9 Hz, 1H, H-9), 2.11 – 2.01 (m, 2H, H-11, H-9), 1.96 – 1.87 (m, 1H, H-2), 1.75 (dt, *J* = 13.7, 5.5 Hz, 1H, H-4), 1.68 – 1.62 (m, 1H, H-4), 1.51 (dt, *J* = 13.8, 10.1 Hz, 1H, H-2), 0.87 (d, *J* =6.9 Hz, 3H, H-13).

^13^C NMR (150 MHz, CD_3_OD) δ 177.06 (C-15), 166.66 (C-22), 162.11 (C-19), 139.02 (C-3'), 131.68 (C-17, C-21), 128.41 (C-5’, C-7’), 128.04 (C-4', C-8’), 125.92 (C-6’), 121.65 (C-16), 114.64 (C-18, C-20), 100.95 (C-8), 81.25 (C-5), 75.09 (C-6), 74.65 (C-7), 70.81 (C-1), 70.07 (C-10), 61.60 (C-12), 40.49 (C-2'), 35.11 (C-1'), 34.48 (C-3), 34.15 (C-9), 32.84 (C-11), 28.99 (C-2), 26.61 (C-4), 11.65 (C-13).

MS (ESI) *m/z*: 564 [M + Na]^+^ for C_29_H_35_NO_9_.

**(2S,3R,3aS,4S,4'S,5'R,6S,7aR)-6-((3,4-dimethoxyphenethyl)carbamoyl)-3,3a,4-trihydroxy-5'-methyldecahydro-3H-spiro[benzofuran-2,2'-pyran]-4'-yl 4-hydroxybenzoate (3j)**

^1^H NMR (800 MHz, CD_3_OD) δ 7.94 (d, *J* = 8.7 Hz, 2H, H-17, H-21), 6.83 – 6.81 (m, 3H, H-18, H-20, H-7'), 6.79 (d, *J* = 1.8 Hz, 1H, H-4'), 6.68 (dd, *J* = 8.1, 1.8 Hz, 1H, H-8'), 5.20 (d, *J* = 2.5 Hz, 1H, H-10), 4.08 (t, *J* = 3.2 Hz, 1H, H-5), 4.01 (t, *J* = 11.4 Hz, 1H, H-12), 3.86 (dd, *J* = 10.4, 5.5 Hz, 1H, H-1), 3.80 (s, 3H, H-10'), 3.78 (s, 3H, H-9'), 3.73 (s, 1H, H-7), 3.60 – 3.57 (m, 1H, H-12), 3.30 – 3.26 (m, 2H, H-2'), 2.67 – 2.60 (m, 2H, H-1'), 2.37 (ddd, *J* = 15.0, 11.3, 5.6 Hz, 1H, H-3), 2.10 (m, 3H, H-9, H-11), 1.93 (ddd, *J* = 14.6, 11.7, 3.1 Hz, 1H, H-2), 1.75 (dt, *J* = 14.0, 5.6 Hz, 1H, H-4), 1.69 – 1.62 (m, 1H, H-4), 1.51 (dt, *J* = 13.9, 9.9 Hz, 1H, H-2), 0.87 (d, *J* = 6.9 Hz, 3H, H-13).

MS (ESI) *m/z*: 624 [M + Na]^+^ for C_31_H_39_NO_11_.

**(2S,3R,3aS,4S,4'S,5'R,6S,7aR)-3,3a,4-trihydroxy-6-((6-methoxy-6-oxohexyl)carbamoyl)-5'-methyldecahydro-3H-spiro[benzofuran-2,2'-pyran]-4'-yl 4-hydroxybenzoate (4a)**

^1^H NMR (600 MHz, CD_3_OD) δ 7.95 (d, *J* = 8.8 Hz, 2H, H-17, H-21), 6.84 (d, *J* = 8.8 Hz, 2H, H-18, H-20), 5.24 (br d, *J* = 2.4 Hz, 1H, H-10), 4.09 (t, *J* = 3.2 Hz, 1H, H-5), 4.02 (t, *J* = 11.2 Hz, 1H, H-12), 3.89 (dd, *J* = 10.4, 5.6 Hz, 1H, H-1), 3.74 (s, 1H, H-7), 3.64 (s, 1H, H-1''), 3.60 (dd, *J* = 11.1, 4.5 Hz, 1H, H-12), 3.10 – 3.02 (m, 2H, H-1'), 2.43 – 2.38 (m, 1H, H-3), 2.30 (t, *J* = 7.4 Hz, 2H, H-5'), 2.16 – 2.06 (m, 3H, H-9, H-11), 1.98 – 1.93 (m, 1H, H-2), 1.81 (dt, *J* = 13.7, 5.5 Hz, 1H, H-4), 1.70 – 1.66 (m, 1H, H-4), 1.64 – 1.52 (m, 3H, H-2, H-2'), 1.43 – 1.38 (m, 2H, H-4'), 1.31 – 1.25 (m, 2H, H-3'), 0.90 (d, *J* = 6.9 Hz, 2H, H-13).

MS (ESI) *m/z*: 588 [M + Na]^+^ for C_28_H_39_NO_11_.

**(2S,3R,3aS,4S,4'S,5'R,6S,7aR)-3,3a,4-trihydroxy-6-((6-isopropoxy-6-oxohexyl)carbamoyl)-5'-methyldecahydro-3H-spiro[benzofuran-2,2'-pyran]-4'-yl 4-hydroxybenzoate (4b)**

^1^H NMR (600 MHz, CD_3_OD) δ 7.95 (br d, *J* = 8.8 Hz, 2H, H-17, H-21), 6.81 (br d, *J* = 8.8 Hz, 2H, H-18, H-20), 5.21 (br d, *J* = 2.2 Hz, 1H, H-10), 4.95 (tt, *J* = 12.6, 6.3 Hz, 1H, H-1''), 4.09 (t, *J* = 3.1 Hz, 1H, H-5), 4.02 (t, *J* = 11.4 Hz, 1H, H-12), 3.88 (dd, *J* = 10.5, 5.6 Hz, 1H, H-1), 3.73 (s, 1H, H-7), 3.59 (dd, *J* = 11.1, 4.7 Hz, 1H, H-12), 3.09 – 3.03 (m, 2H, H-1'), 2.44 – 2.37 (m, 1H, H-3), 2.25 (t, *J* = 7.4 Hz, 2H, H-5'), 2.18 – 2.04 (m, 3H, H-9, H-11), 2.00 – 1.92 (m, 1H, H-2), 1.78 (dt, *J* = 13.9, 5.7 Hz, 1H, H-4), 1.68 (ddd, *J* = 14.4, 5.1, 3.7 Hz, 1H, H-4), 1.61 – 1.50 (m, 3H, H-2, H-2’), 1.45 – 1.37 (m, 2H, H-3'), 1.31 – 1.24 (m, 2H, H-4'), 1.21 (d, *J* = 6.3 Hz, 6H, H-2'', H-3''), 0.87 (d, *J* = 6.9 Hz, 2H, H-13).

MS (ESI) *m/z*: 616 [M + Na]^+^ for C_30_H_43_NO_11_.

**(2S,3R,3aS,4S,4'S,5'R,6S,7aR)-6-((6-butoxy-6-oxohexyl)carbamoyl)-3,3a,4-trihydroxy-5'-methyldecahydro-3H-spiro[benzofuran-2,2'-pyran]-4'-yl 4-hydroxybenzoate (4c)**

^1^H NMR (600 MHz, CD_3_OD) δ 7.94 (d, *J* = 8.8 Hz, 2H, H-17, H-21), 6.81 (d, *J* = 8.8 Hz, 2H, H-18, H-20), 5.21 (br d, *J* = 2.2 Hz, 1H, H-10), 4.09 (t, *J* = 3.1 Hz, 1H, H-5), 4.06 (t, *J* = 6.6 Hz, 2H, H-1''), 4.02 (t, *J* = 11.4 Hz, 1H, H-12), 3.88 (dd, *J* = 10.5, 5.6 Hz, 1H, H-1), 3.73 (s, 1H, H-7), 3.60 – 3.56 (m, 1H, H-12), 3.09 – 3.03 (m, 1H, H-1'), 2.40 (ddd, *J* = 17.9, 8.9, 4.1 Hz, 1H, H-3), 2.29 (t, *J* = 7.4 Hz, 2H, H-5'), 2.17 – 2.04 (m, 3H, H-9, H-11), 1.99 – 1.93 (m, 1H, H-2), 1.78 (tt, *J* = 11.8, 5.9 Hz, 1H, H-4), 1.68 (ddd, *J* = 12.0, 7.9, 3.5 Hz, 1H, H-4), 1.63 – 1.50 (m, 5H, H-2, H-2', H-2''), 1.44 – 1.33 (m, 2H, H-3', H-3''), 1.30 – 1.24 (m, 1H, H-4'), 0.94 (t, *J* = 7.4 Hz, 3H, H-4''), 0.87 (d, *J* = 6.9 Hz, 3H, H-13).

MS (ESI) *m/z*: 630 [M + Na]^+^ for C_31_H_45_NO_11_.

**(2S,3R,3aS,4S,4'S,5'R,6S,7aR)-3,3a,4-trihydroxy-5'-methyl-6-((6-oxo-6-(pentan-2-yloxy)hexyl)carbamoyl)decahydro-3H-spiro[benzofuran-2,2'-pyran]-4'-yl 4-hydroxybenzoate (4d)**

^1^H NMR (600 MHz, CD_3_OD) δ 7.98 (ddd, *J* = 8.8, 2.6, 2.0 Hz, 2H, H-17, H-21), 6.84 (ddd, *J* = 8.8, 2.7, 1.9Hz, 2H, H-18, H-20), 5.24 (br d, *J* = 2.6 Hz, 1H, H-10), 4.94 – 4.91(m, 1H, H-2'' overlap), 4.12 (t, *J* = 3.2 Hz, 1H, H-5), 4.05 (t, *J* = 11.4 Hz, 1H, H-12), 3.90 (dd, *J* = 10.5, 5.6 Hz, 1H, H-1), 3.76 (s, 1H, H-7), 3.61 (dd, *J* = 11.1, 4.4 Hz, 1H, H-12), 3.13 – 3.05 (m, 2H, H-1'), 2.47 – 2.39 (m, 1H, H-3), 2.30 (t, *J* = 7.4 Hz, 2H, H-5'), 2.17 (dd, *J* = 14.8, 3.2 Hz, 1H, H-11), 2.14 – 2.06 (m, 2H, H-9), 1.99 (ddd, *J* = 14.6, 11.7, 3.1 Hz, 1H, H-2), 1.81 (dt, *J* = 13.9, 5.6 Hz, 1H, H-4), 1.72 (ddd, *J* = 14.4, 5.3, 3.6 Hz, 1H, H-4), 1.66 – 1.54 (m, 5H, H-2, H-2', H-3''), 1.52 – 1.26 (m, 8H, H-2', H-3', H-4', H-4''), 1.21 (d, *J* = 6.2 Hz, 3H, H-1''), 0.94 (t, *J* = 7.4 Hz, 3H, H-5''), 0.90 (d, *J* = 6.9 Hz, 3H, H-13).

MS (ESI) *m/z*: 644 [M + Na]^+^ for C_32_H_47_NO_11_.

**(2S,3R,3aS,4S,4'S,5'R,6S,7aR)-3,3a,4-trihydroxy-5'-methyl-6-((6-((4-methylpentan-2-yl)oxy)-6-oxohexyl)carbamoyl)decahydro-3H-spiro[benzofuran-2,2'-pyran]-4'-yl 4-hydroxybenz- oate (4e)**

^1^H NMR (600 MHz, CD_3_OD) δ 7.98 (br d, *J* = 8.8 Hz, 2H, H-17, H-21), 6.84 (br d, *J* = 8.8 Hz, 1H, H-18, H-20), 5.24 (br d, *J* = 2.6 Hz, 1H, H-10), 5.04 – 4.97 (m, 1H, H-2''), 4.12 (t, *J* = 3.2 Hz, 1H, H-5), 4.05 (t, *J* = 11.4 Hz, 1H, H-12), 3.90 (dd, *J* = 10.5, 5.6 Hz, 1H, H-1), 3.76 (s, 1H, H-7), 3.61 (dd, *J* = 11.1, 4.5 Hz, 1H, H-12), 3.13 – 3.05 (m, 1H, H-1'), 2.43 (ddd, *J* = 15.0, 11.3, 5.5 Hz, 1H, H-3), 2.30 (t, *J* = 7.4 Hz, 2H, ), 2.20 – 2.06 (m, 2H), 2.04 – 1.95 (m, 1H), 1.81 (dt, *J* = 13.9, 5.6 Hz, 1H), 1.72 (ddd, *J* = 14.4, 5.3, 3.6 Hz, 1H), 1.68 – 1.53 (m, 3H), 1.47 – 1.41 (m, 1H), 1.33 – 1.26 (m, 2H), 1.21 (d, *J* = 6.2 Hz, 2H), 0.96 – 0.87 (m, 5H).

MS (ESI) *m/z*: 644 [M + H]^+^ for C_33_H_49_NO_11_.

**(2S,3R,3aS,4S,4'S,5'R,6S,7aR)-6-((7-ethoxy-7-oxoheptyl)carbamoyl)-3,3a,4-trihydroxy-5'-methyldecahydro-3H-spiro[benzofuran-2,2'-pyran]-4'-yl 4-hydroxybenzoate (4f)**

^1^H NMR (600 MHz, CD_3_OD) δ 7.97 (br d, *J* = 8.7 Hz, 2H, H-17, H-21), 6.84 (br d, *J* = 8.7 Hz, 2H, H-18, H-20), 5.24 (br d, *J* = 2.1 Hz, 1H, H-10), 4.16 – 4.10 (m, 3H, H-5, H-1''), 4.05 (t, *J* = 11.4 Hz, 1H, H-12), 3.91 (dd, *J* = 10.5, 5.6 Hz, 1H, H-1), 3.76 (s, 1H, H-7), 3.61 (dd, *J* = 11.1, 4.5 Hz, 1H, H-12), 3.13 – 3.04 (m, 2H, H-1'), 2.50 – 2.38 (m, 1H, H-3), 2.32 (t, *J* = 7.4 Hz, 1H, H-6'), 2.17 (dd, *J* = 14.8, 3.1 Hz, 1H, H-11), 2.14 – 2.06 (m, 2H, H-9), 2.03 – 1.96 (m, 1H, H-2), 1.81 (dt, *J* = 13.8, 5.7 Hz, 1H, H-4), 1.74 – 1.68 (m, 1H, H-4), 1.64 – 1.53 (m, 3H, H-2, H-5'), 1.47 – 1.39 (m, 2H, H-2'), 1.37 – 1.22 (m, 7H, H-3', H-4', H-2''), 0.90 (d, *J* = 6.9 Hz, 2H, H-13).

MS (ESI) m/z: 616 [M + Na]^+^ for C_30_H_43_NO_11_.

**(2S,3R,3aS,4S,4'S,5'R,6S,7aR)-6-((7-butoxy-7-oxoheptyl)carbamoyl)-3,3a,4-trihydroxy-5'-methyldecahydro-3H-spiro[benzofuran-2,2'-pyran]-4'-yl 4-hydroxybenzoate (4g)**

^1^H NMR (600 MHz, CD_3_OD) δ 7.98 (ddd, *J* = 8.8, 2.7, 2.0 Hz, 2H, H-17, H-21), 6.85 (ddd, *J* = 8.88, 2.7, 2.0 Hz, 2H, H-18, H-20), 5.24 (br d, *J* = 2.6 Hz, 1H, H-10), 4.12 (t, *J* = 3.2 Hz, 1H, H-5), 4.09 (t, *J* = 6.6 Hz, 2H, H-1''), 4.05 (t, *J* = 11.4 Hz, 1H, H-12), 3.91 (dd, *J* = 10.5, 5.6 Hz, 1H, H-1), 3.76 (s, 1H, H-7), 3.61 (dd, *J* = 11.1, 4.4 Hz, 1H, H-12), 3.14 – 3.04 (m, 2H, H-1'), 2.44 (br ddd, *J* = 15.0, 11.3, 5.6 Hz, 1H, H-3), 2.32 (t, *J* = 7.4 Hz, 1H, H-6'), 2.17 (dd, *J* = 14.8, 3.2 Hz, 1H, H-11), 2.14 – 2.07 (m, 2H, H-9), 2.00 (ddd, *J* = 14.7, 11.7, 3.2 Hz, 1H, H-2), 1.81 (dt, *J* = 14.0, 5.7 Hz, 1H, H-4), 1.71 (ddd, *J* = 14.4, 5.3, 3.5 Hz, 1H, H-4), 1.66 – 1.56 (m, 5H, H-2, H-2', H-2''), 1.46 – 1.25 (m, 8H, H-3', H-3'', H-4', H-5'), 0.97 (t, *J* = 7.4 Hz, 3H, H-4''), 0.90 (d, *J* = 6.9 Hz, 3H, H-13).

MS (ESI) *m/z*: 644 [M + Na]^+^ for C_32_H_47_NO_11_.

**(2S,3R,3aS,4S,4'S,5'R,6S,7aR)-3,3a,4-trihydroxy-6-((8-methoxy-8-oxooctyl)carbamoyl)-5'-methyldecahydro-3H-spiro[benzofuran-2,2'-pyran]-4'-yl 4-hydroxybenzoate (4h)**

^1^H NMR (600 MHz, CD_3_OD) δ 7.97 (d, *J* = 8.7 Hz, 2H, H-17, H-21), 6.84 (d, *J* = 8.7 Hz, 2H, H-18, H-20), 5.24 (d, *J* = 2.2 Hz, 1H, H-10), 4.12 (t, *J* = 3.1 Hz, 1H, H-5), 4.05 (t, *J* = 11.4 Hz, 1H, H-12), 3.91 (dd, *J* = 10.5, 5.6 Hz, 1H, H-1), 3.76 (s, 1H, H-7), 3.67 (s, 3H, H-1''), 3.61 (dd, *J* = 11.1, 4.5 Hz, 1H, H-12), 3.15 – 3.02 (m, 1H, H-1'), 2.47 – 2.40 (m, 1H, H-3), 2.34 (t, *J* = 7.4 Hz, 2H, H-7'), 2.21 – 2.06 (m, 3H, H-9, H-11), 2.03 – 1.96 (m, 1H, H-2), 1.81 (dt, *J* = 13.9, 5.7 Hz, 1H, H-4), 1.74 – 1.68 (m, 1H, H-4), 1.60 – 1.50 (m, 3H, H-2, H-2'), 1.49 – 1.20 (m, 8H, H-3', H-4', H-5', H-6'), 0.90 (d, *J* = 6.9 Hz, 3H, H-13).

MS (ESI) *m/z*: 616 [M + Na]^+^ for C_30_H_43_NO_11_.

**(2S,3R,3aS,4S,4'S,5'R,6S,7aR)-6-((8-ethoxy-8-oxooctyl)carbamoyl)-3,3a,4-trihydroxy-5'-methyldecahydro-3H-spiro[benzofuran-2,2'-pyran]-4'-yl 4-hydroxybenzoate (4i)**

^1^H NMR (600 MHz, CD_3_OD) δ 7.97 (br d, *J* = 8.7 Hz, 2H, H-17, H-21), 6.84 (br d, *J* = 8.7 Hz, 2H, H-18, H-20), 5.24 (br d, *J* = 2.1 Hz, 1H, H-10), 4.18 – 4.09 (m, 3H, H-5, H-1''), 4.05 (t, *J* = 11.4 Hz, 1H, H-12), 3.91 (dd, *J* = 10.5, 5.6 Hz, 1H, H-1), 3.76 (s, 1H, H-7), 3.61 (dd, *J* = 11.1, 4.5 Hz, 1H, H-12), 3.13 – 3.04 (m, 2H, H-1'), 2.50 – 2.40 (m, 1H, H-3), 2.32 (t, *J* = 7.4 Hz, 2H, H-7'), 2.17 (dd, *J* = 14.8, 3.1 Hz, 1H, H-11), 2.14 – 2.05 (m, 2H, H-9), 2.03 – 1.96 (m, 1H, H-2), 1.81 (dt, *J* = 13.9, 5.7 Hz, 1H, H-4), 1.74 – 1.69 (m, 1H, H-4), 1.65 – 1.54 (m, 3H, H-2, H-2'), 1.46 – 1.38 (m, 2H, H-3'), 1.46 – 1.18 (m, 9H, H-4', H-5', H-6', H-2''), 0.90 (d, *J* = 6.9 Hz, 2H, H-13).

MS (ESI) m/z: 630 [M + Na]^+^ for C_31_H_45_NO_11_.

**(2S,3R,3aS,4S,4'S,5'R,6S,7aR)-3,3a,4-trihydroxy-6-((8-isopropoxy-8-oxooctyl)carbamoyl)-5'-methyldecahydro-3H-spiro[benzofuran-2,2'-pyran]-4'-yl 4-hydroxybenzoate (4j)**

^1^H NMR (600 MHz, CD_3_OD) δ 7.86 (ddd, *J* = 8.8, 2.7, 2.0 Hz, 2H, H-17, H-21), 6.73 (ddd, *J* = 8.88, 2.7, 2.0 Hz, 2H, H-18, H-20), 5.12 (br d, *J* = 2.6 Hz, 1H, H-10), 4.86 (dp, *J* = 12.5, 6.3 Hz, 1H, H-1''), 4.00 (t, *J* = 3.2 Hz, 1H, H-5), 3.93 (t, *J* = 11.4 Hz, 1H, H-12), 3.79 (dd, *J* = 10.5, 5.6 Hz, 1H, H-1), 3.64 (s, 1H, H-7), 3.49 (dd, *J* = 11.1, 4.4 Hz, 1H, H-12), 3.02 – 2.92 (m, 2H, H-1'), 2.32 (br ddd, *J* = 15.0, 11.3, 5.6 Hz, 1H, H-3), 2.17 (t, *J* = 7.4 Hz, 2H, H-7''), 2.09 – 1.94 (m, 3H, H-11, H-9, overlap),1.88 (ddd, *J* = 14.6, 9.8, 3.2 Hz, 1H, H-2), 1.69 (dt, *J* = 14.0, 5.7 Hz, 1H, H-4), 1.59 (ddd, *J* = 14.4, 5.3, 3.5 Hz, 1H, H-4), 1.52 – 1.41 (m, 3H, H-2, H-2'), 1.35 – 1.26 (m, 2H, H-3'), 1.24 – 1.05 (m, 12H, H-4', H-5', H-6', H-2'', H-3'', overlap), 0.78 (d, *J* = 6.9 Hz, 2H, H-13).

MS (ESI) *m/z*: 644 [M + Na]^+^ for C_32_H_47_NO_11_.

**(2S,3R,3aS,4S,4'S,5'R,6S,7aR)-6-((8-butoxy-8-oxooctyl)carbamoyl)-3,3a,4-trihydroxy-5'-methyldecahydro-3H-spiro[benzofuran-2,2'-pyran]-4'-yl 4-hydroxybenzoate (4k)**

^1^H NMR (600 MHz, CD_3_OD) δ 7.98 (ddd, *J* = 8.8, 2.7, 2.0 Hz, 2H, H-17, H-21), 6.85 (ddd, *J* = 8.8, 2.7, 2.0 Hz, 2H, H-18, H-20), 5.24 (d, *J* = 2.6 Hz, 1H, H-10), 4.12 (t, *J* = 3.2 Hz, 1H, H-5), 4.09 (t, *J* = 6.6 Hz, 2H, H-1''), 4.05 (t, *J* = 11.4 Hz, 1H, H-12), 3.91 (dd, *J* = 10.5, 5.6 Hz, 1H, H-1), 3.76 (s, 1H, H-7), 3.61 (dd, *J* = 11.1, 4.4 Hz, 1H, H-12), 3.14 – 3.03 (m, 2H, H-1'), 2.44 (ddd, *J* = 15.0, 11.3, 5.6 Hz, 1H, H-3), 2.33 (t, *J* = 7.4 Hz, 2H, H-7'), 2.20 – 2.06 (m, 3H, H-11, H-9, overlap), 2.00 (ddd, *J* = 14.6, 11.7, 3.2 Hz, 1H, H-2), 1.81 (dt, *J* = 14.0, 5.7 Hz, 1H, H-4), 1.71 (ddd, *J* = 14.4, 5.3, 3.5 Hz, 1H, H-4), 1.66 – 1.54 (m, 5H, H-2, H-6', H-2''), 1.48 – 1.21 (m, 10H, H-2', H-3', H-4', H-5', H-3''), 0.97 (t, *J* = 7.4 Hz, 3H, H-4''), 0.90 (d, *J* = 6.9 Hz, 3H, H-13).

MS (ESI) *m/z*: 658 [M + Na]^+^ for C_33_H_49_NO_11_.

**(2S,3R,3aS,4S,4'S,5'R,6S,7aR)-3,3a,4-trihydroxy-5'-methyl-6-((7-oxo-7-(pentan-2-yloxy)heptyl)carbamoyl)decahydro-3H-spiro[benzofuran-2,2'-pyran]-4'-yl 4-hydroxybenzoate (4l)**

^1^H NMR (600 MHz, CD_3_OD) δ 7.97 (ddd, *J* = 8.8, 2.7, 2.0 Hz, 2H, H-17, H-21), 6.84 (ddd, *J* = 8.8, 2.7, 2.0 Hz, 2H, H-18, H-20), 5.24 (br d, *J* = 2.5 Hz, 1H, H-10), 4.92 (brq, *J* = 6.4 Hz, 1H, H-2''), 4.12 (t, *J* = 3.2 Hz, 1H, H-5), 4.05 (t, *J* = 11.4 Hz, 1H, H-12), 3.91 (dd, *J* = 10.5, 5.6 Hz, 1H, H-1), 3.76 (s, 1H, H-7), 3.61 (dd, *J* = 11.1, 4.4 Hz, 1H, H-12), 3.13 – 3.04 (m, 2H, H-1'), 2.44 (ddd, *J* = 15.0, 11.3, 5.6 Hz, 1H, H-3), 2.30 (t, *J* = 7.4 Hz, 2H, H-6'), 2.20 – 2.06 (m, 3H, H-11, H-9), 2.00 (ddd, *J* = 14.6, 9.4, 3.2 Hz, 1H, H-2), 1.81 (dt, *J* = 14.0, 5.7 Hz, 1H, H-4), 1.71 (ddd, *J* = 14.4, 5.3, 3.5 Hz, 1H, H-4), 1.64 – 1.25 (m, 13H, H-12, H-2', H-3', H-4', H-5', H-3'', H-4''), 1.22 (d, *J* = 6.3 Hz, 3H, H-1''), 0.94 (t, *J* = 7.4 Hz, 3H, H-5''), 0.90 (d, *J* = 6.9 Hz, 3H, H-13).

MS (ESI) *m/z*: 636 [M + H]^+^ for C_33_H_49_NO_11_.

**(2S,3R,3aS,4S,4'S,5'R,6S,7aR)-3,3a,4-trihydroxy-5'-methyl-6-((7-((4-methylpentan-2-yl)oxy)-7-oxoheptyl)carbamoyl)decahydro-3H-spiro[benzofuran-2,2'-pyran]-4'-yl 4-hydroxybenz- ate (4m)**

^1^H NMR (600 MHz, CD_3_OD) δ 7.97 (ddd, *J* = 8.8, 2.7, 2.0 Hz, 2H, H-17, H-21), 6.84 (ddd, *J* = 8.8, 2.7, 2.0Hz, 2H, H-18, H-20), 5.24 (br d, *J* = 2.6 Hz, 1H, H-10), 5.04 – 4.98 (m, 1H, H-2'), 4.12 (t, *J* = 3.2 Hz, 1H, H-5), 4.05 (t, *J* = 11.4 Hz, 1H, H-12), 3.91 (dd, *J* = 10.5, 5.6 Hz, 1H, H-1), 3.76 (s, 1H, H-7), 3.61 (dd, *J* = 11.1, 4.4 Hz, 1H, H-12), 3.13 – 3.04 (m, 1H, H-1'), 2.44 (ddd, *J* = 15.0, 11.3, 5.6 Hz, 1H, H-3), 2.30 (t, *J* = 7.4 Hz, 1H, H-7'), 2.17 (dd, *J* = 14.8, 3.2 Hz, 1H, H-11), 2.14 – 2.07 (m, 2H, H-9), 2.00 (ddd, *J* = 14.6, 9.3, 3.2 Hz, 1H, H-2), 1.81 (dt, *J* = 14.0, 5.7 Hz, 1H, H-4), 1.71 (ddd, *J* = 14.4, 5.3, 3.5 Hz, 1H, H-4), 1.68 – 1.54 (m, 5H, H-2, H-2', H-3''), 1.46 – 1.40 (m, 2H, H-3'), 1.37 – 1.26 (m, 5H, H-4', H-5', H-4''), 1.21 (d, *J* = 6.2 Hz, 3H, H-1''), 0.93 (d, *J* = 6.7 Hz, 3H, H-5''), 0.92 (d, *J* = 6.6 Hz, 3H, H-6''), 0.90 (d, *J* = 6.9 Hz, 3H, H-13).

MS (ESI) *m/z*: 650 [M + H]^+^ for C_34_H_51_NO_11_.

**(2S,3R,3aS,4S,4'S,5'R,6S,7aR)-3,3a,4-trihydroxy-6-((6-((1-methoxy-3-methyl-1-oxopentan-2-yl)amino)-6-oxohexyl)carbamoyl)-5'-methyldecahydro-3H-spiro[benzofuran-2,2'-pyran]-4'-yl 4-hydroxybenzoate (4n)**

^1^H NMR (600 MHz, CD_3_OD) δ 7.94 (br d, *J* = 8.7 Hz, 2H, H-17, H-21), 6.81 (br d, *J* = 8.7 Hz, 2H, H-18, H-20), 5.21 (br d, *J* = 2.1 Hz, 1H, H-10), 4.36 (d, *J* = 6.2 Hz, 1H, H-2''), 4.09 (t, *J* = 3.1 Hz, 1H, H-5), 4.02 (t, *J* = 11.4 Hz, 1H, H-12), 3.88 (dd, *J* = 10.5, 5.5 Hz, 1H, H-1), 3.73 (s, 1H, H-7), 3.69 (s, 1H, H-1'''), 3.59 (dd, *J* = 11.1, 4.5 Hz, 1H, H-12), 3.11 – 3.00 (m, 2H, H-1'), 2.40 (tt, *J* = 11.2, 5.6 Hz, 1H, H-3), 2.27 – 2.20 (t, 2H, H-5'), 2.18 – 2.12 (m, 1H, H-9), 2.11 – 2.04 (m, 2H, H-11, H-9), 1.99 – 1.93 (m, 1H, H-2), 1.85 (br qt, *J* = 13.4, 6.8 Hz, 1H, H-3''), 1.78 (dt, *J* = 13.9, 5.6 Hz, 1H, H-4),1.72 – 1.66 (m, 1H, H-4), 1.62 – 1.20 (m, 9H, H-2), 0.93 – 0.89 (m, 6H, H-5', H-6'), 0.87 (d, *J* = 6.9 Hz, 1H, H-3).

MS (ESI) *m/z*: 701 [M + Na]^+^ for C_34_H_50_N_2_O_12_.

**(2S,3-R,3aS,4S,4'S,5'R,6S,7aR)-3,3a,4-trihydroxy-6-((6-((1-methoxy-3-methyl-1-oxobutan-2-yl)amino)-6-oxohexyl)carbamoyl)-5'-methyldecahydro-3H-spiro[benzofuran-2,2'-pyran]-** **4'-yl 4-hydroxybenzoate (4o)**

^1^H NMR (600 MHz, CD_3_OD) δ 7.95 (br d, *J* = 8.7 Hz, 2H, H-17, H-21),6.82 (br d, *J* = 8.7 Hz, 2H, H-18, H-20), 5.21 (br d, *J* = 2.1 Hz, 1H),4.30 (d, *J* = 6.2 Hz, 1H), 4.09 (t, *J* = 3.0 Hz, 1H, H-5), 4.02 (t, *J* = 11.4 Hz, 1H, H-12), 3.88 (dd, *J* = 10.5, 5.5 Hz, 1H, H-1), 3.74 (s, 1H, H-7), 3.70 (s, 3H), 3.59 (dd, *J* = 11.0, 4.9 Hz, 1H, H-12), 3.12 –3.02 (m, 2H), 2.40 (tt, *J* = 11.3, 5.6 Hz, 1H, H-3), 2.25 (t, *J* =7.5 Hz, 2H), 2.18 – 2.04 (m, 4H, H-9, H-11), 1.99 – 1.92 (m, 2H, H-2), 1.78 (dt, *J* = 13.9, 5.6 Hz, 1H, H-4), 1.72– 1.66 (m, 2H, H-4), 1.63 – 1.50 (m, 3H, H-2), 1.47 – 1.38 (m, 2H), 1.32 – 1.24 (m, 2H), 0.94 (dd, *J* = 6.8, 2.8 Hz, 6H), 0.87 (d, *J* = 6.9 Hz, 3H).

MS (ESI) *m/z*: 687 [M + Na]^+^ for C_33_H_48_N_2_O_12_.

**(2S,3R,3aS,4S,4'S,5'R,6S,7aR)-3,3a,4-trihydroxy-6-((8-((1-methoxy-1-oxo-3-phenylpropan-2-yl)amino)-8-oxooctyl)carbamoyl)-5'-methyldecahydro-3H-spiro[benzofuran-2,2'-pyran]-4'-yl 4-hydroxybenzoate (4p)**

^1^H NMR (600 MHz, CD_3_OD) δ 7.85 (d, *J* = 8.7 Hz, 2H, H-17, H-21), 7.19 – 7.15 (m, 2H, H-6', H-8'), 7.11 (t, *J* = 6.6 Hz, 3H, H-5', H-7', H-9'), 6.72 (d, *J* = 8.7 Hz, 2H, H-18, H-20), 5.12 (d, *J* = 2.4 Hz, 1H, H-10), 4.58 (dd, *J* = 9.4, 5.5 Hz, 1H, H-2''), 4.01 (t, *J* = 3.2 Hz, 1H, H-5), 3.93 (t, *J* = 11.4 Hz, 1H, H-12), 3.79 (dd, *J* =10.5, 5.6 Hz, 1H, H-1), 3.65 (s, 1H, H-7), 3.59 (s, 3H, H-1'''), 3.50 (dd, *J* = 11.1, 4.6Hz, 1H, H-12), 3.06 (dd, *J* = 13.9, 5.5 Hz, 1H, H-3''), 3.00 – 2.91 (m, 2H, H-1'), 2.83 (dd, *J* = 13.9, 9.5 Hz, 1H, H-3''), 2.32 (qd, *J* = 11.3, 5.6 Hz, 1H, H-3), 2.09 – 1.95 (m, 5H, H-9, H-11, H-7'), 1.88 (ddd, *J* = 14.6, 11.8, 3.1 Hz, 1H, H-2), 1.70 (dt, *J* = 13.9, 5.7 Hz, 1H, H-4), 1.62 – 1.56 (m, 1H, H-4), 1.46 (dt, *J* = 13.9, 9.9 Hz, 1H, H-2), 1.42– 1.35 (m, 2H, H-2'), 1.33 – 1.03 (m, 8H, H-3', H-4', H-5', H-6'), 0.78 (d, *J* = 6.9 Hz, 3H, H-13).

MS (ESI) *m/z*: 763 [M + Na]^+^ for C_39_H_52_N_2_O_12_.

**Preparation of biotin probes**

**Biotin linker (5, blank control**)

Biotin (10 mg, 4.1×10^-5^ mol) was stirred with 1,4-diiodobutane (27 μL, 2.1×10^-4^ mol) and potassium carbonate (28.3 mg, 2.1×10^-4^ mol) in DMF (1.0 mL) at room temperature until a complete conversion was detected. The mixture was concentrated. The crude product was purified by silica gel column chromatography to obtain the corresponding titled compound as a white solid (14.0 mg, 80.5%).

^1^H NMR (600 MHz, CD_3_OD) δ 4.48 (dd, *J* = 7.7, 4.9 Hz, 1H), 4.30 (dd, *J* = 7.8, 4.5 Hz, 1H), 4.10 (t, *J* = 6.3 Hz, 2H), 3.31 – 3.29 (m, 2H), 3.26 (t, *J* = 6.8 Hz, 2H), 3.23 – 3.18 (m, 1H), 2.93 (dd, *J* = 12.8, 5.0 Hz, 1H), 2.70 (d, *J* = 12.7 Hz, 1H), 2.35 (t, *J* = 7.3 Hz, 2H), 1.91 – 1.85 (m, 2H), 1.78 – 1.71 (m, 3H), 1.66 (dd, *J* = 16.2, 7.8 Hz, 2H), 1.60 (d, *J* = 8.4 Hz, 1H), 1.45 (dd, *J* = 15.5, 7.7 Hz, 2H).

MS (ESI) *m*/*z*: 427 [M+H]^+^ for C_14_H_23_INO_3_S.

**PAC5-biotin probe (positive probe)**

Compound **3d** (10 mg, 1.97×10^-5^ mol) was stirred with biotin linker **5** (3.28×10^-5^ mol) and potassium carbonate (11.3 mg, 8.2×10^-5^ mol) in dry DMF (1.0 mL) at room temperature until a complete conversion was detected. The mixture was concentrated. The crude product was purified by silica gel column chromatography to obtain the corresponding titled compound as a white solid (13.5 mg, 84.8%).

^1^H NMR (600 MHz, CD_3_OD) δ 7.94 (d, *J* = 8.9 Hz, 2H), 6.88 (d, *J* = 8.9 Hz, 2H), 5.14 (d, *J* = 2.5 Hz, 1H), 4.38 (dd, *J* = 7.8, 4.6 Hz, 1H), 4.19 (dd, *J* = 7.9, 4.5 Hz, 1H), 4.06 (t, *J* = 6.1 Hz, 2H), 4.01 (t, *J* = 3.3 Hz, 1H), 3.98 (t, *J* = 6.0 Hz, 2H), 3.93 (t, *J* = 11.4 Hz, 1H), 3.79 (dd, *J* = 10.4, 5.6 Hz, 1H), 3.65 (s, 1H), 3.50 (dd, *J* = 11.1, 4.5 Hz, 1H), 3.11 – 3.06 (m, 1H), 3.00 – 2.92 (m, 2H), 2.81 (dd, *J* = 12.8, 5.0 Hz, 1H), 2.60 (d, *J* = 12.7 Hz, 1H), 2.38 – 2.30 (m, 1H), 2.24 (t, *J* = 7.3 Hz, 2H), 2.06 (dd, *J* = 14.8, 3.2 Hz, 1H), 1.99 (dd, *J* = 14.8, 2.8 Hz, 2H), 1.88 (ddd, *J* = 14.6, 11.7, 3.2 Hz, 1H), 1.81 – 1.72 (m, 4H), 1.70 – 1.66 (m, 1H), 1.58 (dddd, *J* = 22.9, 19.9, 9.9, 5.1 Hz, 4H), 1.48 (ddd, *J* = 14.1, 10.3, 6.5 Hz, 2H), 1.32 (ddt, *J* = 22.2, 14.7, 7.4 Hz, 4H), 1.25 – 1.18 (m, 2H), 1.14 (ddd, *J* = 11.8, 7.1, 2.1 Hz, 2H), 0.82 – 0.74 (m, 6H).

MS (ESI) m/z: 828 [M+Na]^+^ for C_40_H_59_N_3_O_12_S.

**PAC3-biotin probe (negative probe)**

Compound **3b** (5.3 mg, 1.10×10^-5^ mol) was stirred with biotin linker **5** (9.4 mg, 2.2×10^-5^ mol) and potassium carbonate (7.7 mg, 5.5×10^-5^ mol) in dry DMF (1.0 mL) at room temperature until a complete conversion was detected. The mixture was concentrated. The crude product was purified by silica gel column chromatography to obtain the corresponding titled compound as a white solid (1.3 mg, 15.1%).

^1^H NMR (800 MHz, CD_3_OD) δ 8.03 (d, *J* = 8.9 Hz, 2H), 6.97 (d, *J* = 6.1 Hz, 2H), 5.23 (d, *J* = 2.7 Hz, 1H), 4.61 – 4.57 (m, 3H), 4.48 (dd, *J* = 7.9, 4.5 Hz,1H), 4.29 (dd, *J* = 7.9, 4.5 Hz, 1H), 4.16 (td, *J* = 6.3, 3.2 Hz, 2H), 4.12 (t, *J* = 3.2 Hz, 1H), 4.08 (t, *J* = 5.9 Hz, 2H), 4.04 (t, *J* = 11.5 Hz, 1H), 3.89 (dd, *J* = 10.3, 5.6 Hz, 1H), 3.76 (s, 1H), 3.60 (dd, *J* = 11.5, 4.6 Hz, 1H), 3.34 (s, 1H), 3.21 – 3.16 (m, 1H), 3.08 –2.97 (m, 1H), 2.90 (dd, *J* = 12.7, 4.9 Hz, 1H), 2.69 (d, *J* = 12.7 Hz, 1H), 2.46 – 2.39 (m, 1H), 2.32 (td, *J* = 7.4, 1.9 Hz, 1H), 2.16 (dd, *J* = 14.8, 3.2 Hz, 1H), 2.09 (dd, *J* = 14.8, 3.2 Hz, 2H), 1.97 (td, *J* = 14.8, 3.4 Hz, 1H), 1.89 – 1.85 (m, 2H), 1.85 – 1.78 (m, 3H), 1.74 – 1.63 (m, 4H), 1.61 – 1.55 (m, 2H), 1.46 – 1.39 (m, 4H), 1.30 (d, 1H), 0.88 (d, *J* = 6.9 Hz, 3H), 0.83 (t, *J* = 7.4 Hz, 3H).

MS (ESI) m/z: 800 [M+Na]^+^ for C_38_H_55_N_3_O_12_S.

**Preparation of rhodamine B probes**

**Rhodamine B linker (6, blank control)**

Rhodamine B (100 mg, 2.25×10^-4^ mol) was stirred with EDCI (80 mg, 4.18×10^-4^ mol), DMAP (25.5 mg, 2.09×10^-4^ mol) and 2-bromoethanol (45 μL, 4.18×10^-4^mol) in dichloromethane (2.0 mL) under reflux until a complete conversion was detected. The mixture was concentrated. The crude product was purified by silica gel column chromatography to obtain the linker as a red solid (115 mg, 92.6%).

^1^H NMR (600 MHz, CDCl_3_) δ 8.34 (dd, *J* = 8.0, 0.9 Hz, 1H), 7.85 (td, *J* = 7.6, 1.2 Hz, 1H), 7.77 (td, *J* = 7.8, 1.2 Hz, 1H), 7.34 (dd, *J* = 7.6, 0.8 Hz, 1H), 7.09 (dd, *J* = 9.5, 4.8 Hz, 2H), 6.94 (dd, *J* = 9.5, 2.4 Hz, 2H), 6.83 (d, *J* = 2.4 Hz, 2H), 5.31 (s, 1H), 4.31 (d, *J* = 5.3 Hz, 2H), 3.66 (dt, *J* = 14.5, 7.3 Hz, 8H), 3.53 (t, *J* = 5.3 Hz, 2H), 1.34 (t, *J* = 7.1 Hz, 12H).

MS (ESI) m/z 573: [M+H]^+^ for C_30_H_34_BrN_2_O_3_.

**PAC5-RB probe (positive probe)**

Compound **3d** (20 mg, 3.94×10^-5^ mol) was stirred with rhodamine B linker **6** (65.1 mg, 1.18×10^-4^ mol) and potassium carbonate (27.3 mg, 1.97×10^-4^ mol) in dry DMF (1.0 mL) at room temperature until a complete conversion was detected. The mixture was concentrated. The crude product was purified by silica gel column chromatography to obtain the corresponding titled compound as a red solid (12.4 mg, 32.2%).

^1^H NMR (600 MHz, CD_3_OD) δ 8.49 (dd, *J* = 7.9, 1.1 Hz, 1H), 7.87 (ddd, *J* = 10.5, 7.2, 1.7 Hz, 3H), 7.82 (dd, *J* = 7.8, 1.2 Hz, 1H), 7.42 (dd, *J* = 7.5, 0.9 Hz, 1H), 7.17 (d, *J* = 9.5 Hz, 1H), 7.14 (d, *J* = 9.5 Hz, 1H), 7.06 (dd, *J* = 9.5, 2.4 Hz, 2H), 7.00 (dd, *J* = 10.4, 2.4 Hz, 2H), 6.76 (dd, *J* = 6.9, 1.9 Hz, 2H), 3.75 – 3.72 (m, 2H), 3.69 (dt, *J* = 14.8, 7.3 Hz, 9H), 3.62 (d, *J* = 4.8 Hz, 1H), 3.03 (dd, *J* = 7.3, 3.1 Hz, 2H), 2.40 – 2.29 (m, 1H), 2.23 (dd, *J* = 14.7, 2.9 Hz, 1H), 1.77 (d, *J* = 14.9 Hz, 3H), 1.70 – 1.65 (m, 2H), 1.59 – 1.48 (m, 3H), 1.40 – 1.35 (m, 3H), 1.31 (dt, *J* = 7.1, 6.1 Hz, 18H), 1.24 – 1.19 (m, 3H), 0.91 – 0.87 (m, 5H), 0.74 (d, *J* = 6.9 Hz, 3H).

MS (ESI) m/z: 999 [M+H]^+^ for C_56_H_70_N_3_O_12_^＋^.

**PAC3-RB probe (negative probe)**

Compound **3b** (6.0 mg, 1.25×10^-5^ mol) was stirred with rhodamine B linker **6** (20.7 mg, 3.75×10^-5^ mol) and potassium carbonate (8.7 mg, 6.26×10^-4^ mol) in dry DMF (0.7 mL) at room temperature until a complete conversion was detected. The mixture was concentrated. The crude product was purified by silica gel column chromatography to obtain the corresponding titled compound as a red solid (2.5 mg, 21.2%).

^1^H NMR (600 MHz, CD_3_OD) δ 8.49 (dd, *J* = 7.9, 0.8 Hz, 1H), 7.87 (dd, *J* = 6.6, 5.0 Hz, 2H), 7.81 (td,, *J* = 7.9, 1.1 Hz, 1H), 7.42 (d,, *J* = 7.4 Hz, 1H),7.17 (d, *J* = 9.5 Hz, 1H), 7.14 (dd,, *J* = 9.5 Hz, 1H), 7.06 (dd, *J* = 9.5, 2.4 Hz, 2H), 7.00 (dd, *J* = 10.4, 2.4 Hz, 2H), 6.76 (d, *J* = 8.8 Hz, 2H), 3.83 (t,, *J* = 11.4 Hz, 1H), 3.73 (d, *J* = 4.9 Hz, 1H), 3.69 (dd, *J* = 14.5, 7.3 Hz, 6H), 3.62 (t,, *J* = 4.9 Hz, 1H), 3.03 – 2.98 (m, 2H), 2.38 – 2.31 (m, 1H), 2.24 (dd, *J* = 14.7, 1.5 Hz, 1H), 1.83 – 1.72 (m, 3H), 1.67 (d, *J* = 14.7 Hz, 1H), 1.59 – 1.49 (m, 2H), 1.42 – 1.36 (m, 2H), 1.31 (dt, *J* = 17.3, 8.6 Hz, 12H), 0.92 – 0.86 (m, 1H), 0.83 (t, *J* = 7.4 Hz, 3H), 0.74 (d, *J* = 6.9 Hz, 3H).

MS (ESI) *m/z*: 957 [M+H]^+^ for C_54_H_66_N_3_O_12_.
